# Characterizing bidirectional transitions in mild cognitive impairment and post‐reversion based on longitudinal neuroimaging and cognitive assessments

**DOI:** 10.1002/alz.70263

**Published:** 2025-05-19

**Authors:** Yao Qin, Jing Cui, Durong Chen, Hongjuan Han, Hongyan Cao, Rong Zhang, Hao Zhu, Meiling Zhang, Hongmei Yu

**Affiliations:** ^1^ Department of Health Statistics School of Public Health, Shanxi Medical University Taiyuan Shanxi China; ^2^ MOE Key Laboratory of Coal Environmental Pathogenicity and Prevention, Shanxi Medical University Taiyuan Shanxi China; ^3^ Division of Health Statistics Shanxi Provincial Key Laboratory of Major Diseases Risk Assessment, Shanxi Medical University Taiyuan Shanxi China

**Keywords:** Alzheimer's disease, mild cognitive impairment, multistate, reversion, transition probability

## Abstract

**INTRODUCTION:**

Characterizing transitions of cognitive state, including reversion from mild cognitive impairment (MCI) to normal cognition (NC) and subsequent cognitive stability or deterioration for post‐reversion, has so far remained limited.

**METHODS:**

Using a retrospective cohort of subjects with an MCI diagnosis at study entry and at least two follow‐up visits between 2005 and December 2022, we developed a functional multistate model framework to estimate longitudinal patterns of transition probabilities between different cognitive states.

**RESULTS:**

The probability of reversion increased from 2% at baseline to a maximum of 8% by year 10 before gradual decline thereafter. For post‐reversion, the probability of progression to MCI rose from 8% to 35.71% at Year 10 and subsequently stabilized.

**DISCUSSION:**

The instantaneous risk of MCI progressing was similar to the risk of re‐progression to MCI for post‐reversion. Post‐reversion subjects remained at an increased risk of cognitive deterioration.

**Highlights:**

- The instantaneous risk of MCI progressing to AD is similar to the risk of re‐progression to MCI for post‐reversion.
- The FMSM we developed effectively utilizes multiple longitudinal markers to reveal variable transition patterns between different cognitive states.
- Considering both spatiotemporal dimensions and sparse irregularities from longitudinal neuroimaging and neuropsychological scales, fMLFPCA and MVFPCA help to extract variation patterns, to capture detailed changes characterizing the multidimensional evolution patterns of MCI.

## 1 BACKGROUND

**FIGURE 1:**
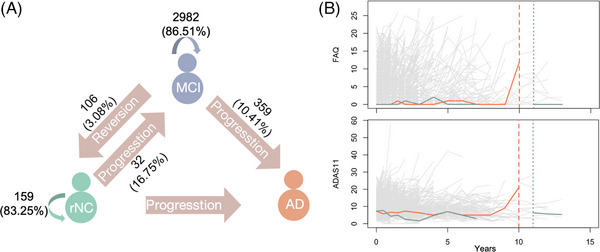
Dynamic cognitive trajectories in mild cognitive impairment (MCI). (A) Multidimensional evolutionary patterns of MCI and the number of transitions between cognitive states, with circles representing cognitive states and arrows indicating possible transitions between states. (B) Longitudinal observations and cognitive outcomes for two individuals, where the orange solid line is the observations of ADAS11 and FAQ for Individual 1, the long dashed line represents the time to progression to AD, the green solid line is the observations of another individual, and the short dashed line represents the time to reversion to NC.

Within the continuum of Alzheimer's disease (AD), mild cognitive impairment (MCI) represents a pivotal intermediate cognitive state between normal cognition (NC) and AD. Given the dynamic and complex interplay between biological mechanisms and psychological factors underlying cognitive aging,^1^ the evolution of cognitive status in MCI, whether it remains stable or undergoes transitions, and the timing of such changes encompasses a spectrum of multidimensional patterns, such as progression to AD in the absence of effective interventions or reversion to NC, as shown in Figure 1A. Yu identified associated factors of MCI reversion and constructed an analytical framework integrating baseline clinical data, magnetic resonance imaging (MRI) scans, and neuropsychological scales over a 2‐year follow‐up period.^2^ Several reviews have examined the reversion rates, associated factors, and prognostic models from diverse perspectives.^3^, ^4^, ^5^, ^6^, ^7^ Our previous work characterized the bidirectional transitions of MCI and explored cognitive trajectories of post‐reversion using longitudinal cognitive assessments.^8^ Based on these findings, we propose that MCI may exhibit a dual nature, characterized by being either “benign” with a substantial potential of reversion or “malignant” with a high risk of dementia. Indeed, the temporal dynamics of MCI reversion and subsequent cognitive stability or deterioration for post‐reversion has so far remained limited.^5^, ^9^

The continuous advancement of diagnostic technologies has been paralleled by the formidable challenge of managing an exponential growth of brain structural information from high‐resolution, three‐dimensional neuroimaging such as MRI. In recent decades, numerous studies have made remarkable improvements in the characterization of AD progression patterns using neuroimaging‐based feature extraction techniques to leverage volumetric information embedded in specific brain structures at a single time point through statistics or machine learning.^10^, ^11^, ^12^ However, it is crucial to recognize that the pathological progression of AD is a long‐term process, analogous to an accelerated aging trajectory accompanied by structural changes. Therefore, the temporal features, absent in single‐time‐point analyses, constitute a crucial information source to be considered. The complex spatial location and physiological mechanisms of brain structures inherently endow neuroimaging with two‐dimensional characteristics in both time and space, underscoring the urgent need for appropriate methodologies capable of comprehensively capturing and integrating spatiotemporal information that is often overlooked in current research.

Moreover, in existing studies on the reversion from MCI to NC, massive neuropsychological scales were extensively used, covering multiple cognitive domains such as memory, language, attention, executive function, visuospatial ability, and anxiety and depression.^13^, ^14^, ^15^, ^16^, ^17^, ^18^, ^19^, ^20^ These studies, however, did not consider potential synergistic effects among multiple longitudinal markers and were limited to baseline measurements. In practice, longitudinal data are discretely collected at unequal intervals and with limited observation sessions (Figure 1B), inadvertently leading to a neglect of the dynamic and continuous underlying processes that remain uncaptured or unattainable. Developing powerful effective modeling techniques is essential for handling such complex longitudinal data.

This study aimed to explore two fundamental issues: (1) How do we characterize the dynamic trajectories of underlying cognitive processes and brain structures, given neuroimaging resources and longitudinal neuropsychological scales across various cognitive domains? (2) How do we establish a quantitative framework for modeling the temporal dynamics of the bidirectional transitions in MCI and quantifying covariate effects, including reversion to NC and progression to AD, as well as post‐reversion?

## 2 METHODS

### 2.1 Samples

The data in this study were obtained from the Alzheimer's Disease Neuroimaging Initiative (ADNI) database, a multicenter and longitudinal observational study aiming to integrate extensive data from neuroimaging, clinical, and neuropsychological assessments to track AD progression. Written informed consent was obtained for all participants. Detailed information on the study protocols, including inclusion and exclusion criteria, is available at http://www.adni‐info.org/. We constructed a retrospective cohort by reviewing subjects from 2005 to December 2022. Subjects were included if they (1) were diagnosed with MCI at study entry and (2) underwent at least two visits during the study period until AD diagnosis or until December 2022, whichever came first. Cognitive states were systematically reassessed approximately every 6 months or annually, defined as any of the following: rNC, MCI, or AD. Notably, rNC refers to subjects who reverted to NC after an initial MCI diagnosis. We further divided subjects with MCI into three subgroups: rMCI (those who experienced at least one cognitive reversion), sMCI (those who remained stable), and pMCI (those who eventually progressed to AD). We excluded observations after an AD diagnosis to render the data compatible with the subsequent multistate modeling framework. Detailed diagnostic criteria for assessing cognitive status are provided in the Supplementary material.

### 2.2 Variables

Global memory performance was assessed using the Alzheimer's Disease Assessment Scale‐Cognitive subscale (ADAS), specifically including ADAS11, ADAS13, and ADASQ4.^21^ Episodic memory function was measured by the Rey Auditory Verbal Learning Test (RAVLT), encompassing immediate recall, learning across trials, forgetting, and percentage of forgetting.^22^ The Trail Making Test Part B (TMT‐B) served as a comprehensive measure of executive function.^23^ The Functional Assessment Questionnaire (FAQ) was administered to quantify daily living abilities.^24^ Additionally, we applied standardized scores for ADNI modified Preclinical Alzheimer's Cognitive Composite (PACC) with Digit Symbol Substitution (denoted as mPACCdigit) and Trails B (referred to as mPACCtrailsB).^21^ Detailed descriptions and calculation methods regarding cognitive assessments are available in the supplementary material. It is worth noting that diagnostic criteria in the ADNI database encompassed both the Mini‐Mental State Examination and Clinical Dementia Rating, hence we excluded these two scales from our study during the subsequent model‐building process.

All structural MRI scans were mapped to a whole‐brain gray matter density space, encompassing 116 brain regions defined by the automated anatomical labeling atlas and segmenting the brain into 90 cerebral subregions and 26 cerebellar subregions.^25^ Due to the long time span of the ADNI study, variations in MRI equipment manufacturers, system configurations, and multicenter data acquisition, sequence parameters of neuroimaging may differ. To enhance the availability and mitigate the impact of interscanner variability, we implemented a standardization procedure, specifically, calculating the gray matter density percentage for each brain region relative to the entire brain tissue. Additionally, relevant demographic and genetic variables (i.e., age, sex, educational attainment, marital status, and APOE ε4 genotype) were incorporated as covariates given their potential effects.

**RESEARCH IN CONTEXT**

1. **Systematic review**: Mild cognitive impairment (MCI) may exhibit a dual nature, characterized by being either “benign” with a substantial potential of reversion or “malignant” with a high risk of AD. However, exploration of the exact timing of MCI reversion to NC and the likelihood of cognitive stability or deterioration for post‐reversion has so far remained limited.
2. **Interpretation**: The instantaneous risk of MCI progressing to AD was similar to the risk of re‐progression to MCI for post‐reversion. Subjects for post‐reversion remained at an increased risk of cognitive deterioration.
3. **Future directions**: Future work should incorporate modifiable factors such as lifestyle and dietary habits to improve individualized prediction models for bidirectional transitions and post‐reversion.

### 2.3 Statistical analysis

The theoretical foundation of this study posits that longitudinal neuropsychological scales and neuroimaging can be conceptualized as functions within continuous temporal or spatial domains but sampled on a discrete grid, a concept referred to as functional data in the statistical literature.^26^, ^27^ In this section, we outline the methodological strategies employed to achieve our dual objectives: dimensionality reduction and dynamic modeling. As shown in the graphical abstract, for the first objective, we utilized multivariate functional principal component analysis (MVFPCA) to capture the various patterns and temporal dynamics of multiple longitudinal cognitive assessments. We then applied multilevel functional principal component analysis (MLFPCA) to extract the spatiotemporal information related to gray matter volume ratios. Also, we performed sensitivity analyses using univariate FPCA for single longitudinal neuropsychological scale and gray matter volume ratios. To accomplish the second objective, we developed a functional multistate model (FMSM) to incorporate the aforementioned functional longitudinal markers and characterize observed transition patterns between different cognitive states following an initial diagnosis of MCI.

### 2.4 Multivariate FPCA

Core functional data analysis (FDA) techniques may be powerful tools to address issues often overlooked by traditional methods, which typically focus on simplistic summary statistics. Assuming that observations of longitudinal markers are derived from a latent longitudinal process, FPCA extracts the overall trends of cognitive trajectories embedded within these longitudinal markers across the entire sample, projecting the infinite‐dimensional functional curves onto a set of lower‐dimensional FPCs through a linear combination of a limited number of basis functions, thereby realizing dimensionality reduction much lower than the entire longitudinal history.^28^ The resulting uncorrelated FPCs then serve as key variables in subsequent statistical modeling. Given the substantial correlations among longitudinal markers, the multivariate FPCA (MVFPCA) approach assumes that all relevant marker trajectories follow a latent multidimensional stochastic process and summarizes into a set of MVFPCs, thereby effectively addressing issues of multicollinearity and interpretability in regression analyses.

### 2.5 Multilevel FPCA

Neuroimaging can be conceptualized as a rich source of multilevel functional data, where spatial organization represents one level and temporal dynamics another.

Traditional multilevel FPCA (tMLFPCA) extends multilevel models and measurement errors to the case where functions serve as the basic units of measurement, ultimately extracting two sets of independent FPCs, namely, subject‐specific FPCs (Level 1) and visit‐specific FPCs (Level 2). Specifically, tMLFPCA employs functional analysis of variance (FANOVA) to explicitly decompose total variation into within‐ and between‐subject variation within the functional space, facilitating FPCA at both levels. However, despite the conceptual elegance of tMLFPCA, its direct manipulation of high‐dimensional covariance matrices presents computational challenges that limit its application to large‐scale functional data. To address this, the fast MLFPCA (fMLFPCA) extends fast covariance estimation techniques to multilevel function data and achieves level‐specific eigendecomposition and FPC scores based on mixed model equations, substantially improving the processing speed by several orders of magnitude. Further technical details regarding FPCA are available in the Supplementary material.

By setting the cumulative proportion of variance explained (PVE) at a threshold of 90% and inspecting the scree plot derived from eigenvalues, we retained the MVFPCs or fMLFPCs derived from multiple longitudinal markers as a new set of derived variables.

### 2.6 FMSM

A multistate model is a continuous‐time stochastic process framework where subjects may transition between a finite set of disease states during follow‐up. We treated cognitive states, including rNC, MCI, and AD, as state space and time‐on‐study as the time scale from one state to another. MCI and rNC were defined as transient states and AD as an absorbing state. Transition intensity refers to the overall rate of transition, quantifying the instantaneous risk of, for instance, transition from MCI to NC. Transition probability provided a probabilistic view of dynamic changes between cognitive states, representing the likelihood of a certain transition at a given time. Additional subject‐related variables, such as demographic characteristics, can also be incorporated into an intensity matrix.

In this study, we extended the multistate model within the FDA framework, which enabled us to jointly model the historical observations of longitudinal markers and time‐to‐event information. Let $q_{\mathrm{rs}}^{\left(0\right)}$ be the baseline transition intensity from cognitive state r to s, and let $\gamma_{i}$ be the vector of regression coefficients. The elements of the transition intensity matrix Q in the FMSM were specified as

qrst=qrs0exp+γ5APOEε4i+∫SgisBsdsγ1agei+γ2sexi+γ3educationi+γ4marriagei+γ5APOEε4i+∫SgisBsds,

qrst=qrs0exp+γ5APOEε4i+∫Tgi,psBisdsγ1agei+γ2sexi+γ3educationi+γ4marriagei+γ5APOEε4i+∫Tgi,psBisds,

qrst=qrs0exp+γ5APOEε4i+∫∫Ωgis,tBs,tdtdsγ1agei+γ2sexi+γ3educationi+γ4marriagei+γ5APOEε4i+∫∫Ωgis,tBs,tdtds,

where $g_{i}\left(s\right)$ is the functional variable derived from the one‐dimensional domain $s\varepsilon [0,S_{\max}]=\;S$, such as the spatial structure of the brain, not consistent with the temporal domain. The coefficient function B(s) is in the same domain as $g_{i}\left(s\right)$. Let $g_{i,p}\left(s\right)$ be different longitudinal neuropsychological scales and $\int \;\;\;\int_{\Omega }g_{i}\left(s,t\right)B\left(s,t\right)\mathrm{dtds}$ be longitudinal neuroimaging in the spatiotemporal two‐dimensional domain.

We specified multiple preplanned FMSMs. Specifically, FMSM 0 included demographic characteristics and the APOE ε4 genotype as covariates. Based on FMSM 0, FMSM 1 incorporated MVFPC scores derived from 11 longitudinal neuropsychological scales, thereby capturing the temporal evolution of cognition. FMSMs 2 and 3 integrated longitudinal neuroimaging data through FPC scores in the spatial domain and fMLFPC scores in the spatiotemporal two‐dimensional domain, respectively. FMSMs 4 and 5 further combined longitudinal markers from FMSMs 1+2 and 1+3, respectively. To perform a sensitivity analysis, we also employed a conventional principal component analysis (PCA) for extracting key information from the longitudinal cognitive assessments and neuroimaging, subsequently constructing a scalar multistate model (MSM). Similarly, MSM 1 incorporated PC scores derived from the same 11 longitudinal neuropsychological scales, MSM 2 integrated longitudinal neuroimaging data through principal component (PC) scores, and MSM 3 combined longitudinal markers from MSMs 1 and 2.

Bootstrapping was employed to assess the robustness of parameter estimates based on asymptotic standard errors calculated from the Hessian, that is, by drawing random samples from the assumed multivariate normal distribution of the maximum likelihood estimates and covariance matrix and appropriate transformations. The likelihood ratio test was performed, with the likelihood values and Akaike information criterion (AIC) for model comparison. Comparison of observed and expected transition numbers and percentages over a specified time period and visualization through graphical representations are constructed for model assessment.

## 3 RESULTS

Data in this study included 4657 observations from 1019 subjects with a MCI diagnosis at study entry. The median follow‐up time was 2 years (interquartile range 1, 4 years), with total follow‐up ranging from 0.417 to 14.67 years. We identified 359 (10.41%) transitions from MCI to AD, 106 (3.08%) transitions from MCI to NC, and 2982 (86.51%) remaining in the MCI state. For rNC, we observed 32 (16.75%) transitions from rNC to MCI and 159 (83.25%) remaining in the rNC state. At the end of the study, the cohort consisted of 93 rMCI, 567 sMCI, and 359 pMCI. Descriptive statistics for key baseline variables are presented in Table S1.

### 3.1 Information extraction from temporal dimension of cognition

The importance of the FPCs obtained from univariate FPCA for each longitudinal neuropsychological scale is detailed in Table S2 in terms of PVEs. For example, the first three FPCs of ADAS11 accounted for 58.23%, 21.63%, and 10.13% of the total variation, respectively. The first FPC (FPC1) captured more than 58% of the total variation, with each subsequent FPC capturing the largest remaining portion of variation. There were significant differences in the distribution patterns of FPC1 among the different MCI subgroups. Specifically, for neuropsychological scales such as ADAS11, ADAS13, ADASQ4, FAQ, RAVLT Forgetting, RAVLT Percentage of Forgetting, and TMT‐B, the variance proportion of FPC1 adhered to a consistent order: pMCI > sMCI > rMCI. Conversely, for scales including RAVLT Immediate Recall, RAVLT Learning Across Trials, mPACCdigit, and mPACCtrailsB, the trend reversed, with rMCI > sMCI > pMCI, highlighting the distinct cognitive profiles associated with each MCI subgroup.

**FIGURE 2:**
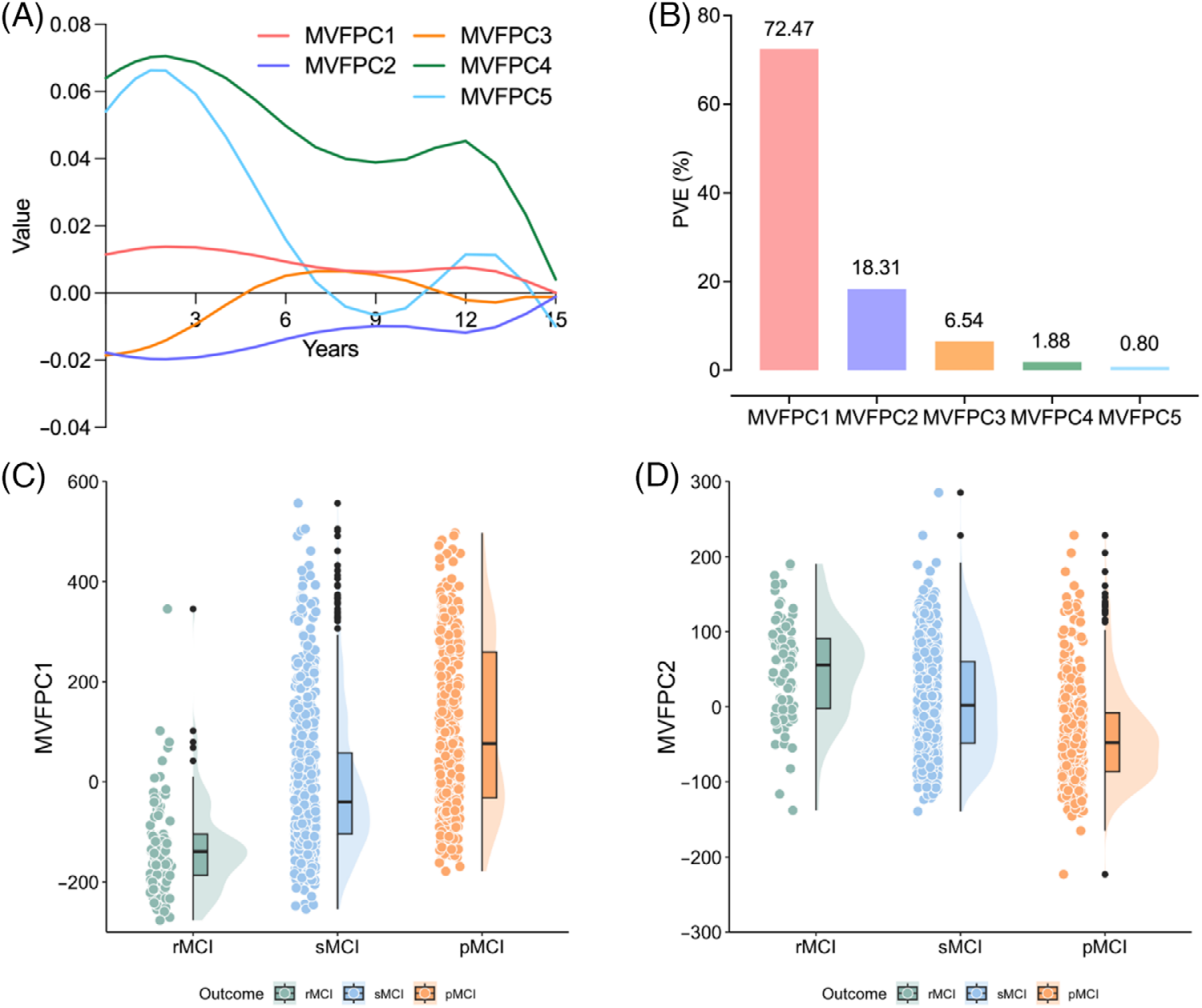
Information extracted from the temporal dimension of cognition using multivariate functional principal component analysis (MVFPCA). (A) The trajectory curves of five eigenfunctions extracted in the case of ADAS11. (B) Proportions of variance explained of these top five functional principal components. (C) The distribution patterns of MVFPC1 among the different mild cognitive impairment (MCI) subgroups. (D) The distribution patterns of MVFPC2 among the different MCI subgroups.

Given the high correlations among multiple cognitive assessments, we applied MVFPCA to further explore the in‐depth information of longitudinal cognitive trajectories embedded within disease progression and identified five primary variation patterns. Figures 2A illustrates the trajectory curves of these five eigenfunctions in the case of ADAS11, with FPC1 constantly above zero and FPC2 constantly below zero, suggesting that the main variability among cognitive assessments arises from the weighted average of their cognitive trajectories. FPC3 crossed the *x*‐axis twice and was primarily positive in [5, 11] and negative elsewhere, reflecting different cognitive trajectories during this specific period. FPC4 fluctuated above zero with a downward trend, whereas FPC5 crossed the *x*‐axis three times, remaining positive in [0, 7] and [11, 14]

As shown in Figure 2B, the first two MVFPCs collectively explained over 90% of the cognitive variance, with specific PVEs of 72.47% and 18.31%, respectively. Subjects with rMCI exhibited the lowest MVFPC1 score and the highest MVFPC2 score, while subjects with pMCI had the opposite pattern, indicating that higher MVFPC1 scores and lower MVFPC2 scores were indicative of more severe cognitive impairment (Figure 2C and D). As confirmed by the observed curves of neuropsychological scales, rMCI generally maintained better cognitive and daily function, whereas pMCI experienced a sharp decline, as illustrated in Figure S1. Thus, the first two MVFPCs emerged as robust indicator variables, capable of capturing the majority of variations associated with bidirectional transitions in MCI. Meanwhile, the remaining MVFPCs provided complementary, albeit more limited, information. For a comprehensive understanding of these findings, additional details are provided in Figures S2 to S6.

### 3.2 Information extraction from spatiotemporal two‐dimensionality of brain structures

**FIGURE 3:**
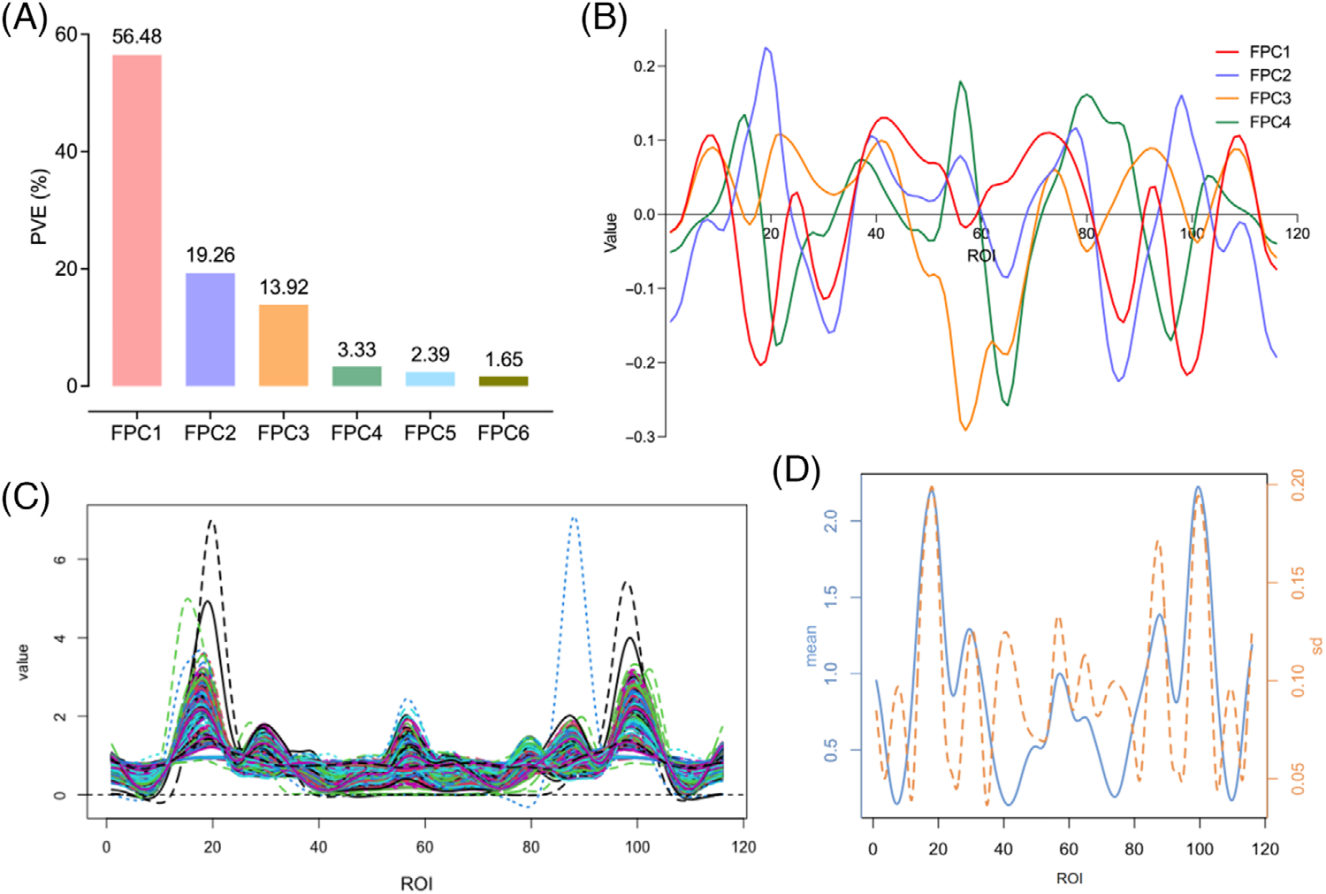
Information extracted from brain structures in spatial domain of using functional principal component analysis (FPCA). (A) The proportions of variance explained of first top six FPCs. (B) The trajectory curves of first four eigenfunctions. (C) Smooth curves of gray matter volume ratios for all subjects. (D) Mean and standard deviation curves.

In our implementation of FPCA within the brain spatial domain, we observed that the first FPC captured 56.48% of the total variation in gray matter volume ratios. The cumulative contribution of the first four FPCs reached 92.99%, highlighting their dominance in explaining underlying patterns. Figures 3A,B respectively illustrate the PVEs of these top four FPCs and their corresponding eigenfunction curves. As depicted in Figure 3C, the overall trend of gray matter volume ratios exhibited an evident regional symmetry across both hemispheres, with the gray matter volume ratios on the ordinate corresponding to different brain regions along the abscissa (Table S3). The mean and standard deviation curves in Figure 3D underscore the overall trend similarity in gray matter volume ratios across subjects, higher in the bilateral frontal lobes and lower in the bilateral occipital and temporal lobes. Additionally, we identified greater variability in specific regions, including the left limbic lobe, subcortical gray nucleus, and frontal lobe, as well as the right parietal lobe, central region, and frontal lobe, suggesting potential regional differences in gray matter plasticity during disease progression.

**FIGURE 4:**
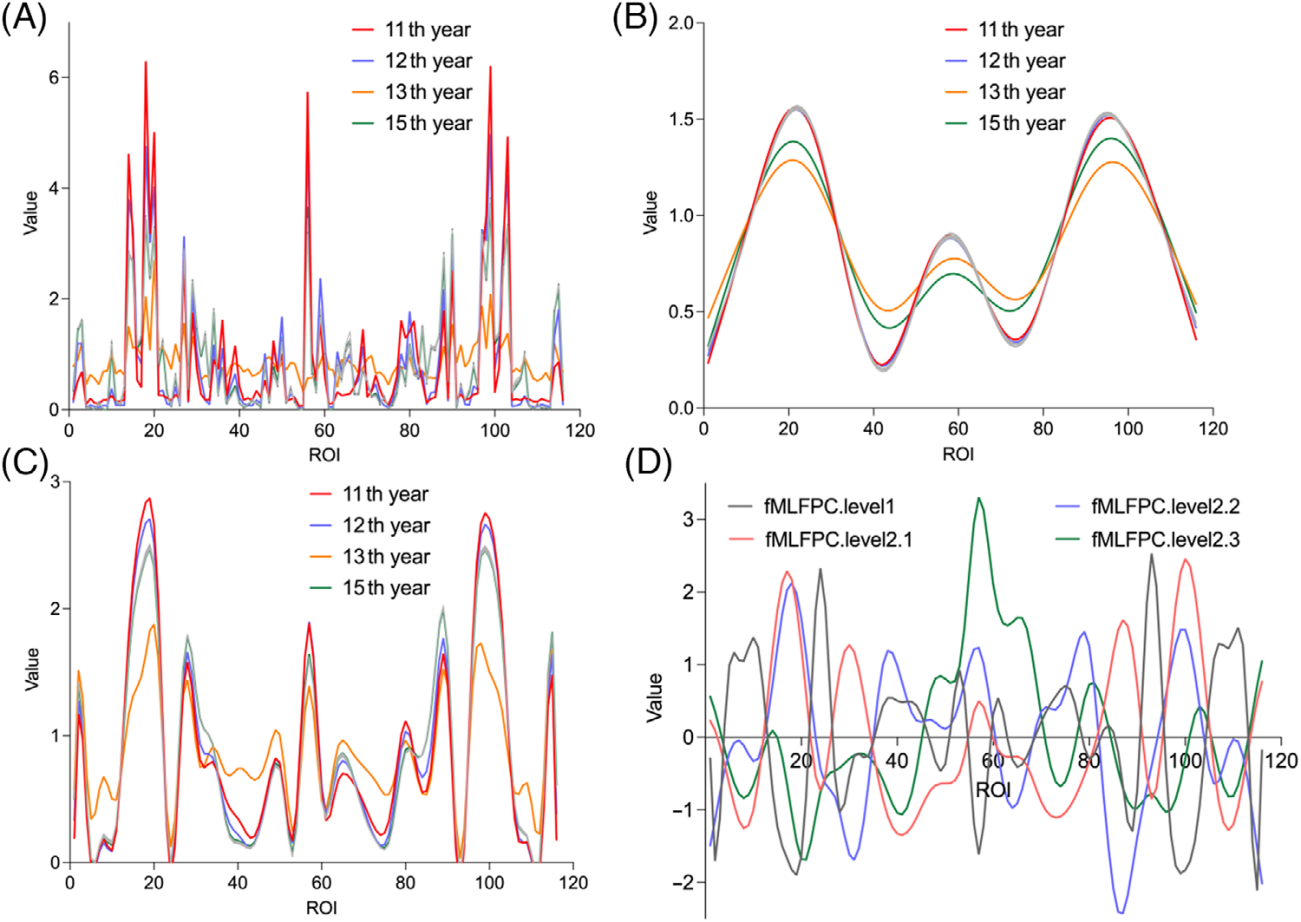
Information extracted from spatiotemporal two‐dimensionality of brain structures. (A) An individual's original gray matter volume ratios. (B) Functional curves derived from traditional multilevel functional principal component analysis (tMLFPCA). (C) Functional curves derived from fast MLFPCA (fMLFPCA). (D) Eigenfunction curves of temporal fMLFPC and three spatial fMLFPC using fMLFPCA, with the following order of abscissa: (left) insula (1) → limbic lobe (2 to 8) → subcortical gray nucleus (9 to 13) → frontal lobe (14 to 26) → central region (27 to 29) → parietal lobe (30 to 34) → occipital lobe (35 to 41) → temporal lobe (42 to 45) → cerebellum (46 to 54) → vermis (55 to 62) → (right) cerebellum (63 to 71) → temporal lobe (72 to 75) → occipital lobe (76 to 82) → parietal lobe (83 to 87) → central region (88 to 90) → frontal lobe (91 to 103) → subcortical gray nucleus (104 to 108) → limbic lobe (109 to 115) → insula (116).

We applied tMLFPCA and fMLFPCA to extract spatiotemporal two‐dimensional information on longitudinal gray matter volume ratios. For instance, consider a male individual with 18 years of education, married, and without the APOE ε4 allele, who reversed at the 11th year and maintained stable cognitive status for the following four years. A comparative analysis with this individual's original gray matter volume ratios (Figure 4A) indicated that the functional curve derived from tMLFPCA (Figure 4B) exhibited an excessively smoothed profile, tending to obscure important structural changes. In contrast, the functional curves generated by fMLFPCA (Figure 4C) provided a more accurate depiction of brain structural dynamics, revealing finer details and temporal variations. The smooth curves spanning the first decade closely aligned, consistent with the stable MCI status of this individual, followed by significant changes in gray matter volume ratios in the 11th year, peaking in the left frontal lobe, cerebellar vermis, and right frontal lobe, while showing the lowest volume ratios in the right cerebellum, related to the individual's cognitive improvement. To further illuminate these spatiotemporal patterns, we present a temporal fMLFPC and three spatial fMLFPC eigenfunction curves in Figure 4D. These curves offer insights into the underlying multilevel functional variations that contributed to the observed gray matter volume ratios. For a comprehensive explanation of our findings, including additional statistical details and visualizations, please refer to Tables S4 to S6 and Figures S7 to S10.

### 3.3 Dynamic modeling of bidirectional transitions of MCI

**FIGURE 5:**
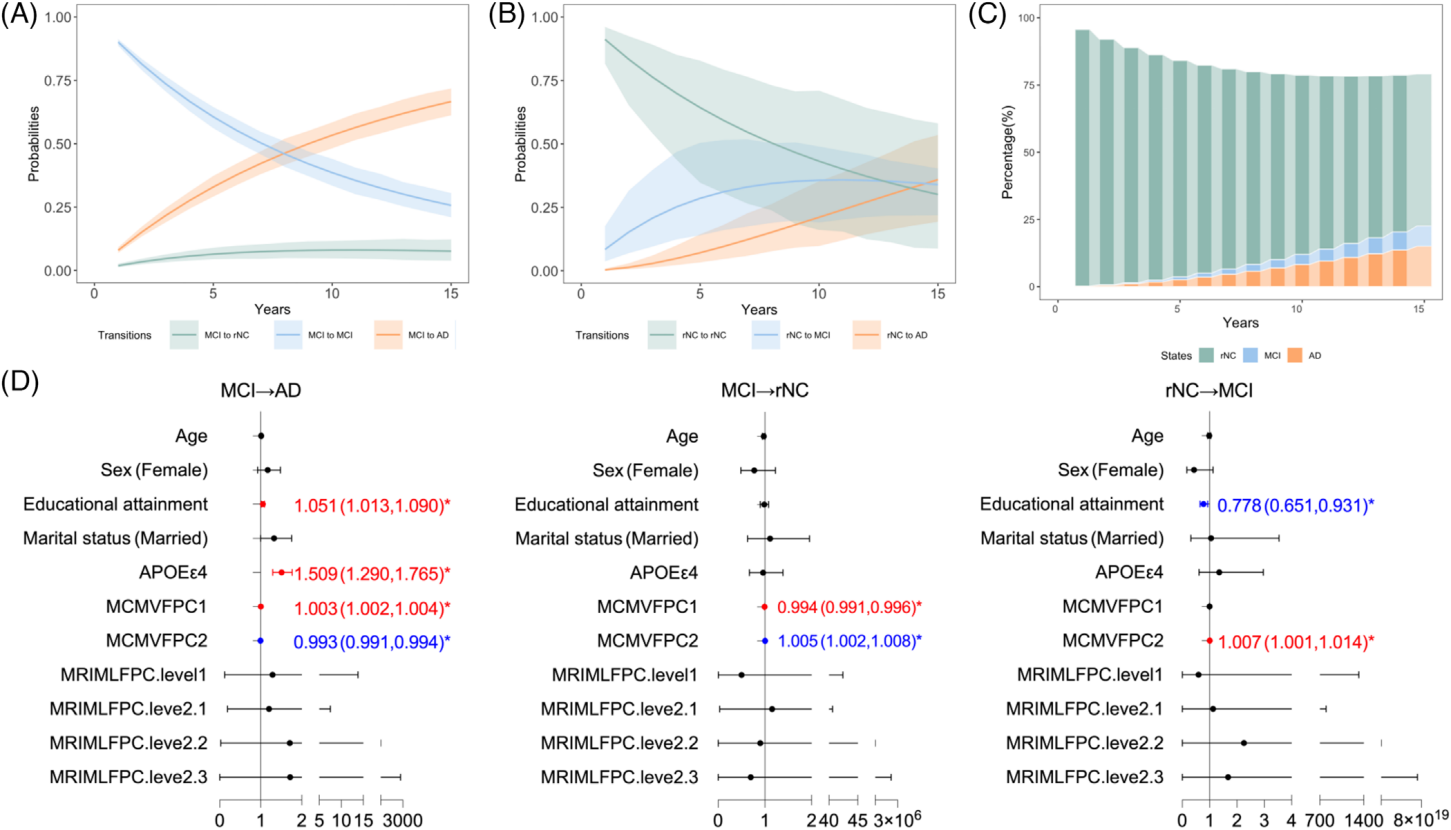
Quantitative characterization of bidirectional transitions of mild cognitive impairment (MCI). (A) Transition probability curves between different cognitive states for MCI. (B) Transition probability curves between different cognitive states for reversion to normal cognition (rNC). (C) Estimated percentage of total length of stay for three states over 15 years. (D) Effect of covariates on state transitions of MCI → AD, MCI → rNC, and rNC → MCI (left to right).

The transition intensity from MCI to NC was the smallest at 0.035, whereas the intensity of progressing to MCI for rNC was 3.34 times that of the former, which closely approximated the transition from MCI to AD at 0.120. We included the FPC scores derived from longitudinal markers as new variables to quantitatively characterize bidirectional transitions in MCI. Here, the estimated results of Model 5 are presented in this paper. In Figure 5A for MCI, the probability of remaining in the same state within 7 years (blue lines) was higher than the probability of transitioning to a subsequent state; not surprisingly, the likelihood of subsequent deterioration to AD (orange line) exceeded the other transitions. The probability of reversion increased from 2% at baseline to a maximum of 8% by Year 10 and then declined at a slower rate thereafter (green line). In Figure 5B for rNC, the probability of progression to MCI rose from 8% to 35.71% at Year 10 and subsequently stabilized (blue line). The likelihood of progression to AD increased annually, with a slope similar to the that of the transition from MCI to AD (orange line). As illustrated in Figure 5C, more than half of the time was spent in the rNC state. Figure 5D summarizes the covariate effects on different transitions. Higher education level, APOE ε4 carrier status, higher MVFPC1 score, and lower MVFPC2 score were significantly associated with MCI progressing to AD. A lower MVFPC1 score and higher MVFPC2 score were protective factors for reversion. Interestingly, lower education level and higher MVFPC2 score were identified as risk factors for rNC progressing to MCI. Details on the estimation results of all models constructed in this study are presented in Tables S7 to S37.

The observed and expected transition percentage curves for rNC closely aligned, showing a high degree of agreement at each observation point. For MCI and AD, the percentage curves matched well during the first 5 years but started to diverge thereafter, with an overestimation for MCI and an underestimation for AD. This discrepancy may be due to the decreasing number of older individuals available for follow‐up over time, while the risk of dementia tends to increase with longer follow‐up periods (Figures S11 to S26).

## 4 DISCUSSION

From the perspective of FDA, we introduced a novel framework utilizing cutting‐edge FDA methodologies to reduce the dimensionality of neuroimaging and cognitive assessments collected at multiple time points and capture both temporal and spatial dynamics and facilitating subsequent dynamic modeling based on all historical data. We observed significant variations across several brain regions, including the bilateral frontal lobe, left limbic lobe, subcortical gray nucleus, right parietal lobe, and central region. The first FPC of longitudinal cognitive assessments differed significantly among MCI subgroups. Additionally, we developed a FMSM framework that integrated the spatiotemporal variation patterns mentioned above to characterize the bidirectional transition dynamics in MCI. We found that the instantaneous risk of MCI progressing to AD was similar to the risk of re‐progression to MCI for rNC. Subjects with rNC remained at an increased risk of progression to MCI, consistent with our previous study.^8^

Due to the high heterogeneity of diagnostic criteria, follow‐up duration, geographic regions, and demographic characteristics, existing estimates of reversion rate exhibited substantial heterogeneity.^16^ A meta‐analysis in 2016 revealed an overall reversion rate of approximately 24%, with notable deference between clinical (14%) and community‐based samples (31%).^4^ However, a surprising observation from the ADNI cohort, where our estimated maximum reversion rate over a 15‐year period was at a mere 8%, accompanied by a wide confidence interval for cognitive trajectories of rNC in Figure 5B. Recent studies applied neuropsychological (NP) criteria, encompassing domains such as memory, language, attention, and executive functions, to reclassify MCI and NC, and have been confined to the baseline‐to‐Year 1 interval, potentially overlooking long‐term cognitive fluctuations.^20^, ^29^, ^30^ Our study had a relatively long follow‐up period of up to 15 years, enabling a more thorough assessment of cognitive stability.

Cognitive changes during the disease progression are not static but dynamic and continuous. Considering the cost‐effective and ease of use of neuropsychological scales, cognitive cohort studies typically indirectly approximate underlying cognitive changes through long sequences of observations.^31^ The high correlation and potential non‐linearity among cognitive assessments induce multicollinearity, ultimately yielding biased parameter estimates and reduced statistical power. Consequently, traditional linear models prove inadequate for capturing the intricate underlying structures. Furthermore, in real‐world studies like ADNI, only a few repeated measures are available per subject by intermittent collection. FDA methodology offers a flexible alternative to traditional statistical analysis by treating discrete, repeated observations as smooth curves or continuous functions. Instead of analyzing a simple sequence of individual observations, FDA considers the continuous curve trajectories as the primary research unit. The latent trajectory of each marker is modeled as a smoothing process, addressing challenges such as missed visits and irregular observations and reducing the reliance on strict data acquisition schedules and fixed time intervals.^32^, ^33^, ^34^

In our study, we summarized multiple longitudinal cognitive assessments into a set of uncorrelated FPC scores. While FPC scores were derived from implicit functions of multivariate marker trajectories and may lack direct clinical interpretability, we conducted additional analyses using ADNI samples to better understand the FPCs from each neuropsychological scale. Our findings, presented in Figure S3, indicated that the majority of variability in cognition stemmed from directions closely aligned with the overall mean estimates. Furthermore, contrasts between early, mid, and later time points significantly contributed to the variability in cognitive trajectories. This suggested that the successful capture of critical FPC information, reflecting additive effects from dynamic changes in cognitive assessments that were less straightforward in parametric models, favored the application of FPCA.^35^ Moreover, FDA reduces the dimensionality of longitudinal markers, often using FPC scores to enhance the ability to characterize the temporal dynamics of neurodegenerative disease progression using survival models. For instance, Li applied MVFPCA to summarize multiple longitudinal neuropsychological scales into a set of MVFPC scores, which were then used as explanatory variables in a Cox regression model to construct a functional two‐stage prognostic model.^36^, ^37^ Similarly, Lee used hippocampal surface data as functional covariates and brain volumes as scalar covariates to model the progression of AD.^38^ When the primary focus is on understanding the temporal evolution and interdependence among longitudinal markers, such as cognitive assessments, MVFPCA can identify common patterns of change across subjects and reveal how these patterns relate to outcomes of interest, such as transitions in cognitive status. MLFPCA extends this capability to handle more complex nested structures such as longitudinal gray matter volume data where multiple MRI scans are nested within an individual. By incorporating the hierarchical nature of data, MLFPCA captures not only within‐individual temporal variations but also spatial variability that reflects individual differences and common trends in brain structures. Hence, MLFPCA offers a more nuanced understanding of brain structural changes, potentially identifying more subtle relationships that may be overlooked by MVFPCA. When combined, these functional PCA methods play a unique but complementary role in modeling multivariate temporal trajectories in multilevel functional data and can leverage their respective strengths to improve longitudinal pattern resolution.

In this study, we successfully integrated FDA with a MSM to develop a novel FMSM framework. Specifically, we employed MVFPCA and fMLFPCA to extract variation patterns from longitudinal neuropsychological scales and neuroimaging, considering both spatiotemporal dimensions and sparse irregularities and enabling us to capture detailed changes in complex dynamic processes and characterize the multidimensional evolution patterns of MCI. Regular FPCA can be used to identify brain structures of interest when only baseline data are available. From Model 1 to Model 5, longitudinal cognitive assessments and neuroimaging were successively incorporated into our FMSMs. Overall, Models 4 and 5 demonstrated similar explanatory power compared to Model 1 and did not decrease the AIC, implying that adding more longitudinal neuroimaging markers may not significantly improve model discrimination when the cognitive assessments were already included in the model. Similarly, Li observed limited enhancement in explanatory capacity when neuroimaging markers were added to models already incorporating cognitive assessments.^35^ Subsequently, this time‐dependent information was then incorporated into a FMSM framework to estimate transition probabilities between cognitive states. This step‐by‐step approach not only simplified the complexity of the problem but also maintained high applicability. Therefore, the powerful combination of FDA and multistate survival modeling offers a new perspective for statistical analysis of high‐dimensional longitudinal survival data.

This study has several limitations. First, our study design focused on characterizing transition dynamics rather than predictive modeling, which inherently limited the interpretability of our results. When stratifying the total follow‐up duration across 1019 subjects, we observed that individuals with shorter follow‐up duration tended to exhibit more severe cognitive impairment (Figures S27 and S28). This is further reinforced by Figure S29, which shows that the transition intensity distribution from rNC to MCI derived through bootstrapping has a wider range and limited precision. This observation may be partly attributed to the nature of the ADNI cohort as a convenience sample, primarily consisting of voluntary participants, potentially resulting in an elevated representation of individuals with cognitive impairments and a corresponding underrepresentation of those who reverted to NC. Future studies should prioritize external cohorts to validate these non‐predictive mechanistic associations and build individualized dynamic prediction tools through distinct methodologies (e.g., joint modeling of longitudinal and time‐to‐event data), followed by model optimization and the development of user‐friendly interfaces (e.g., web‐based calculators), ultimately enhancing clinical applicability and translating analytical outputs into actionable clinical decisions. Second, although utilizing longitudinal whole‐brain structural neuroimaging, our work was confined to region‐of‐interest‐based volumetric measurements derived from prior‐knowledge‐guided brain tissue segmentation. To gain a more nuanced understanding of spatial variations and subtle patterns in brain structure, whole‐brain voxel‐wise analysis represents a promising direction for future endeavors. Third, our investigation focused solely on APOE ε4, a representative genetic variant associated with cognitive progression. Extending our FMSM framework to incorporate multiple genetic variants through the polygenic risk scores approach could offer valuable insights.

The instantaneous risk of MCI progressing to AD was similar to the risk of re‐progression to MCI for post‐reversion. Subjects with rMCI remained at an increased risk of cognitive deterioration. In summary, our study developed a FMSM framework that effectively utilized multiple longitudinal markers to characterize bidirectional transitions in MCI, revealing complex and variable transition patterns between different cognitive states and providing a theoretical foundation and statistical support for studying the natural history of other chronic diseases.

## CONFLICT OF INTEREST STATEMENT

The authors declare that they have no conflicts of interest. Author disclosures are available in supporting information.

## CONSENT STATEMENT

Written informed consent was obtained for all participants, and the study protocol was approved by the Institutional Review Board at each participating center of the ADNI database before protocol‐specific procedures were performed.

## Supporting information



Supporting Information

Supporting Information
