# Supplementary material for: Characterizing bidirectional transitions in mild cognitive impairment and post‐reversion based on longitudinal neuroimaging and cognitive assessments
Source: Alzheimers Dement. 2025 May 19;21(5):e70263. doi: 10.1002/alz.70263 (PMC12086984; doi:10.1002/alz.70263)
Supplement: Supplementary file 1 — Supporting Information [file ALZ-21-e70263-s002.docx]

**METHODS**

***Diagnostic criteria***

***NC* (*normal cognition*)**

1. Subject must be free of memory complaints, verified by a study partner, beyond what one would expect for age.

2. Normal memory function documented by scoring above education adjusted cutoffs on the Logical Memory II subscale (Delayed Paragraph Recall, Paragraph A only) from the Wechsler Memory Scale –Revised (the maximum score is 25):

a. ≥9 for 16 or more years of education

b. ≥5 for 8-15 years of education

c. ≥3 for 0-7 years of education.

3. Mini-Mental State Exam (MMSE) score between 24 and 30 (inclusive). (Exceptions may be made for subjects with less than 8 years of education at the discretion of the project director).

4. Clinical Dementia Rating (CDR) = 0. Memory Box score must be 0.

5. Cognitively normal, based on an absence of significant impairment in cognitive functions or activities of daily living.

***MCI* (*mild cognitive impairment*)**

1. Subject must have a subjective memory concern as reported by subject, study partner, or clinician.

2. Abnormal memory function documented by scoring within the education adjusted ranges on the Logical Memory II subscale (Delayed Paragraph Recall, Paragraph A only) from the Wechsler Memory Scale –Revised (the maximum score is 25):

a. ≤11 for 16 or more years of education

b. ≤9 for 8-15 years of education

c. ≤6 for 0-7 years of education.

3. MMSE score between 24 and 30 (inclusive). (Exceptions may be made for subjects with less than 8 years of education at the discretion of the project director).

4. CDR = 0.5. Memory Box score must be at least 0.5.

5. General cognition and functional performance sufficiently preserved such that a diagnosis of Alzheimer’s disease (AD) cannot be made by the site physician at the time of the screening visit.

***AD*(*Alzheimer’s disease*)**

1. Subject must have a subjective memory concern as reported by subject, study partner, or clinician.

2. Abnormal memory function documented by scoring within the education adjusted ranges on the Logical Memory II subscale (Delayed Paragraph Recall, Paragraph A only) from the Wechsler Memory Scale –Revised (the maximum score is 25):

a. ≤8 for 16 or more years of education

b. ≤4 for 8-15 years of education

c. ≤2 for 0-7 years of education.

3. MMSE score between 20 and 26 (inclusive) (Exceptions may be made for subjects with less than 8 years of education at the discretion of the project director).

4. CDR = 0.5 or 1.0.

5. The National Institute of Neurological and Communicative Disorders and Stroke-Alzheimer Disease and Related Disorders Association (NINCDS/ADRDA) criteria for probable AD.

***Cognitive assessments***

***ADAS11***

The Alzheimer's Disease Assessment Scale-Cognitive Subscale (ADAS-Cog) is specifically designed to evaluate the severity of cognitive impairment across the mild to severe spectrum of Alzheimer's Disease (AD). ADAS-Cog assesses various cognitive domains, including memory, language, and praxis. The specific tasks encompass word recall, naming objects and fingers, commands, constructional praxis, ideational praxis, orientation, word recognition, language, comprehension of spoken language, word finding difficulty, and remembering test instructions. Typically, studies incorporate all 11 tasks and score them on a single scale ranging from 0 to 70, referred to as “ADAS-Cog-11”. Higher scores indicate worse performance.

***ADAS13***

ADAS-Cog 13 includes all ADAS-Cog-11 items as well as a test of delayed word recall and a number cancellation or maze task, ranging from 0 to 85. Higher scores indicate worse performance.

***ADASQ4***

ADASQ4, often referred to as ADAS Delayed Word Recall, is a component of the ADAS-cog that assesses memory function, specifically delayed recall. ADASQ4 ranges from 0 to 12. Higher scores indicate worse performance.

***FAQ***

Functional Activities Questionnaire (FAQ) is a brief assessment tool used to evaluate an individual's ability to perform daily living activities, ranging from 0 to 30. Higher scores indicate worse performance.

***RAVLT Immediate Recall***

RAVLT Immediate Recall assesses an individual's ability to recall a list of words immediately after hearing them. RAVLT Immediate Recall provides an indication of short-term memory and immediate recall capabilities. Higher scores indicate better performance. RAVLT Immediate Recall refers to the sum of scores from 5 first trials (Trials 1 to 5).

***RAVLT Learning Across Trials***

RAVLT Learning Across Trials evaluates how well an individual learns and retains information over multiple presentations of the same word list. The word list is presented multiple times (typically five times), and after each presentation, the individual is asked to recall the words. A gradual increase in the number of correctly recalled words across trials suggests effective learning. RAVLT Learning Across Trials refers to the score of Trial 5 minus the score of Trial 1

***RAVLT Forgetting***

RAVLT Forgetting measures the extent to which an individual forgets information over a short period after learning it. After completing the learning trials, the individual is typically given a distraction task for a set period (e.g., 20-30 minutes). Following the distraction, the individual is asked to recall the words again. The number of words forgotten provides an index of forgetting. A higher number of forgotten words indicates greater forgetting. RAVLT Forgetting refers to the score of Trial 5 minus score of the delayed recall.

***RAVLT Percentage of Forgetting***

RAVLT Percentage of Forgetting is derived by dividing the number of words forgotten by the total number of words correctly recalled during the best learning trial, and then multiplying by 100. A higher percentage indicates a greater proportion of forgetting relative to what was learned. RAVLT Percentage of Forgetting refers to RAVLT Forgetting divided by the score of Trial 5.

***TMT-B***

The Trail Making Test-B (TMT-B) is a neuropsychological assessment tool used to evaluate executive function, providing information about an individual's ability to switch between tasks and process complex visual information efficiently. TMT-B ranges from 0 to 25. Higher scores indicate worse performance.

***mPACCdigit***

mPACCdigit stands for ADNI modified Preclinical Alzheimer's Cognitive Composite (PACC) with Digit Symbol Substitution. It is a cognitive assessment tool derived from the original PACC, with the inclusion of the Digit Symbol Substitution test. The mPACCdigit evaluates multiple aspects of cognitive function, including memory, attention, and processing speed. The Digit Symbol Substitution test specifically assesses processing speed and working memory by requiring the individual to match symbols to numbers as quickly as possible.

***mPACCtrailsB***

mPACCtrailsB represents ADNI modified Preclinical Alzheimer's Cognitive Composite (PACC) with Trails B. Trails B is a part of the Trails Making Test, which is a neuropsychological test used to assess cognitive flexibility and set-shifting abilities. In Trails B, the individual is required to connect a series of numbered and lettered circles, alternating between numbers and letters in ascending order. The mPACCtrailsB focuses on evaluating the ability to switch between different tasks and maintain focus.

***Statistical Analysis***

Many studies reported multiple longitudinal markers to assess disease progression, as well as time-to-event data. We adopted a novel method to analyze these data using functional principal component analysis (FPCA) methods for the longitudinal markers and a multi-state model for the survival outcomes.

***Univariate FPCA***

Consider a single longitudinal predictor $Y_{\mathrm{ij}}$ for the $\mathrm{ith}$ individual at visit time $t_{\mathrm{ij}}$, $Y_{\mathrm{ij}}=X_{i}(t_{\mathrm{ij}})+\varepsilon_{\mathrm{ij}}$, $\varepsilon_{\mathrm{ij}}\sim N(0,\sigma_{\varepsilon}^{2})$. We assumed that longitudinal observations were from a latent longitudinal process $X_{i}(s)$, with the overall mean $\mu(s)$ and the covariance function $\Sigma(s,s^{'})=cov\{X_{i}(s),X_{i}(s^{'})\}$. The spectral decomposition of the covariance function was given by $\Sigma(s,s^{'})=\sum_{l=1}^{\infty} \lambda_{l}{\phi_{l}(s)\phi}_{l}(s^{'})$, where $\lambda_{l}$ and $\phi_{l}(s)$ were the non-increasing eigenvalue and corresponding orthonormal eigenfunction, respectively. The Karhunen-Loève expansion of $X_{i}(s)$ was $X_{i}(s)=\mu(s)$+$\sum_{l=1}^{\infty} \xi_{\mathrm{il}}\phi_{l}(s)$, where the FPC scores $\xi_{\mathrm{il}}=\int[X_{i}(s)-\mu(s)]\phi_{l}(s)ds$ were independent random variables, $\xi_{\mathrm{il}}\sim N(0,\lambda_{1})$. To the extent that $\phi_{l}(s)$ was a variation pattern of the longitudinal process $X_{i}(s)$, and $\xi_{\mathrm{il}}$ described how strongly the individual $i$ followed this pattern. Assuming that the first $L$ eigenfunctions were sufficient to approximate the longitudinal process $X_{i}(s)$, a truncated form was given by $X_{i}(s)=\mu(s)$+$\sum_{l=1}^{L} \xi_{\mathrm{il}}\phi_{l}(s)$, where $L$ was determined using the proportion of variance explained (PVE), e.g., 90%.

***Multivariate FPCA***

To deal with interpretation difficulty and multicollinearity issues leading by strong correlation among multiple markers, multivariate FPC (MVFPC) was adopted, instead of the univariate FPCA for each marker individually. MVFPCA merges multiple variables by analyzing their functional data together in a unified framework, extracting shared patterns of variability, and providing a comprehensive representation of their joint dynamics.

The detailed steps are as follows:

**(1) Joint Functional Representation:** Each variable is treated as a function over a domain such as time. These multiple functional variables are represented in a common functional space, where each variable is a curve or a set of functions defined over the same or related domains.

**(2) Covariance Structure:** MVFPCA simultaneously models the covariance structure between the different variables, allowing for the identification of shared and unique sources of variability through a joint covariance operator.

**(3) Eigenfunction Decomposition:** MVFPCA performs eigenfunction decomposition on the joint covariance operator to identify FPCs for each variable, capturing shared patterns of variability across the multiple functional datasets.

**(4) MVFPC:** The resulting MVFPCs are multivariate, representing combinations of individual components from each variable, enable to capture the interrelationships and co-variations between variables.

**(5) Dimensionality Reduction and Interpretation:** MVFPCs offer a lower-dimensional data representation, facilitating the interpretation of complex multi-variable interactions, and enhance understanding of variable co-variation over time, such as cognitive assessments and neuroimaging data.

Methodological details are as follows:

For longitudinal processes $X^{(p)}(t)$ with $P(p=1,...,P)$ markers, the univariate FPCA as described above was first performed for each longitudinal marker, and we obtained estimated eigenfunctions $\hat{\phi}_{l}^{(p)}(t)$ and scores $\hat{\xi}_{\mathrm{il}}^{(p)}$ for the $\mathrm{pth}$ marker. Then we defined a $n\times L_{+}$ matrix $\Theta$, where each row $(\hat{\xi}_{i1}^{(1)},\cdots,\hat{\xi}_{iL_{1}}^{(1)},\cdots,\hat{\xi}_{i1}^{(P)},\cdots,\hat{\xi}_{iL_{P}}^{(P)})$ included all the estimated scores for individual $i$, and $L_{+}=\sum_{p=1}^{P} L_{p}$, with suitable truncation $L_{p}$. For the $L_{+}\times L_{+}$ matrix ${(n-1)}^{-1}\Theta^{T}\Theta$, the matrix eigenanalysis was performed, with eigenvalues ${\{\hat{\nu}_{k}\}}_{k=1,\cdots,L_{+}}$ and orthonormal eigenvectors ${\{\hat{c}_{k}\}}_{k=1,\cdots,L_{+}}$. The MVFPC scores were estimated via $\hat{\rho}_{\mathrm{ik}}=\sum_{p=1}^{P} \sum_{l=1}^{L_{p}} {[\hat{c}_{k}]}_{l}^{(p)}\hat{\xi}_{\mathrm{ipl}}$, where ${[\hat{c}_{k}]}^{(p)}$ was the $\mathrm{pth}$ block of the orthonormal eigenvector $\hat{c}_{k}$. The percentage of variance explained (PVE) was used to select the optimal number $L\leq L_{+}$ of MVFPC.

***Multilevel FPCA***

Multilevel functional data has become increasingly prevalent. Multilevel FPCA (MLFPCA) extends the concept of measurement error and multilevel models to the case where functions are the fundamental unit of measurement, providing an explicit decomposition of both within-subject and between-subject variation in the function space.

The detailed steps are as follows:

(1) Estimate the population mean function $\hat{\mu}(s)$ and the mean shift specific to the j-th observation $\hat{\eta}_{j}(s)$.

(2) Estimate the covariance between individuals $\hat{K}_{B}(s,t)$ using a bivariate smoothing method applied to $\{Y_{ij_{1}}(t_{ij_{1}s})-\hat{\mu}(t_{ij_{1}s})-\hat{\eta}_{j_{1}}(t_{ij_{1}s})\}\{Y_{ij_{2}}(t_{ij_{2}r})-\hat{\mu}(t_{ij_{2}r})-\hat{\eta}_{j_{2}}(t_{ij_{2}r})\}$, and similarly estimate the total covariance $\hat{K}_{T}(s,t)$ for all cross-products $\{Y_{\mathrm{ij}}(t_{\mathrm{ijs}})-\hat{\mu}(t_{\mathrm{ijs}})-\hat{\eta}_{j}(t_{\mathrm{ijs}})\}\{Y_{\mathrm{ij}}(t_{\mathrm{ijr}})-\hat{\mu}(t_{\mathrm{ijr}})-\hat{\eta}_{j}(t_{\mathrm{ijr}})$.

(3) Smooth the estimates of total covariance $\hat{K}_{T}(s,t)$ and between-individual covariance $\hat{K}_{B}(s,t)$, yielding the smoothed estimates $\tilde{K}_{T}(s,t)$ and $\tilde{K}_{B}(s,t)$, $\tilde{K}_{W}(s,t)=\tilde{K}_{T}(s,t)-\tilde{K}_{B}(s,t)$.

(4) Perform eigenanalysis on the discretized smoothed estimates of between-individual $\tilde{K}_{B}(s,t)$ and within-individual covariances $\tilde{K}_{W}(s,t)$.

(5) Estimate the error variance $\hat{\sigma}^{2}=\int_{S} \{\hat{K}_{T}(s,s)-\tilde{K}_{T}(s,s)\}ds$.

Ultimately, MLFPCA reduces the multilevel functional data into two sets of independent MLFPC scores: the level-1 MLFPC scores $\xi_{ik_{1}}$ and the level-2 MLFPC scores $\zeta_{\mathrm{ij}k_{2}}$.

Methodological details are as follows:

Let $Y_{\mathrm{ij}}(s)$ be the observations function of the subject $i$ at the visit $j$ and location $s\in\{s_{1},\cdots,s_{L}\}\in S$. The functional model with measurement errors was defined as

$$Y_{\mathrm{ij}}(s)=X_{\mathrm{ij}}(s)+\epsilon_{\mathrm{ij}}(s)=\mu(s)+\eta_{j}(s)+Z_{i}(s)+W_{\mathrm{ij}}(s)+\epsilon_{\mathrm{ij}}(s)$$

where $\mu(s)$ was the mean function, $\eta_{j}(s)$ was the $\mathrm{jth}$ visit-specific mean shift, $Z_{i}(s)$ was the $\mathrm{ith}$ subject-specific random mean deviation, and $W_{\mathrm{ij}}(s)$ was the $\mathrm{jth}$ visit-specific random mean deviation of $Z_{i}(s)$. The main idea of MLFPCA was to decompose both $Z_{i}(s)$ and $W_{\mathrm{ij}}(s)$ using the Karhunen–Loève (KL) expansions,

$Z_{i}(s)=\sum_{k_{1}}^{N_{1}} \xi_{ik_{1}}\phi_{k_{1}}(s)$, $W_{\mathrm{ij}}(s)=\sum_{k_{2}}^{N_{2}} \zeta_{\mathrm{ij}k_{2}}\psi_{k_{2}}(s)$

where $\xi_{ik_{1}}$ and $\zeta_{\mathrm{ij}k_{2}}$ were the independent FPC scores with zero mean and variance $\lambda_{k_{1}}^{(1)}$ and $\lambda_{k_{2}}^{(2)}$ respectively, and $\phi_{k_{1}}(s)$ and $\psi_{k_{2}}(s)$ were the eigenfunctions of the first level (level-1) and second level (level-2), respectively.

Let the total covariance function $K_{T}$ be $K_{T}(s,t)=cov\{X_{\mathrm{ij}}(s),X_{\mathrm{ij}}(t)\}=K_{B}(s,t)+K_{W}(s,t)$. The covariance function for level-1 processes $K_{B}$ was decomposed as $K_{B}(s,t)=cov\{Z_{i}(s),Z_{i}(t)\}=\sum_{k_{1}}^{N_{1}} \lambda_{k_{1}}^{(1)}\phi_{k_{1}}(s)\phi_{k_{1}}(t)$, called the between-subject covariance function, where $\lambda_{1}^{(1)}\geq\lambda_{2}^{(1)}\geq\cdots0$ was the eigenvalue associated with the orthonormal eigenfunction $\phi_{k_{1}}(s)$. The covariance function for level-2 processes $K_{W}$ was decomposed as $K_{W}(s,t)=cov\{W_{\mathrm{ij}}(s),W_{\mathrm{ij}}(t)\}=\sum_{k_{2}}^{N_{2}} \lambda_{k_{2}}^{(2)}\psi_{k_{2}}(s)\psi_{k_{2}}(t)$, called the within-subject covariance function, where $\lambda_{1}^{(2)}\geq\lambda_{2}^{(2)}\geq\cdots0$ was the eigenvalue associated with the orthonormal eigenfunction $\psi_{k_{2}}(s)$.

Combining the Karhunen–Loève (KL) expansions of $Z_{i}(s)$ and $W_{\mathrm{ij}}(s)$, the functional model about $Y_{\mathrm{ij}}(s)$ was transformed to:

$$Y_{\mathrm{ij}}(s)=\mu(s)+\eta_{j}(s)+\sum_{k_{1}}^{N_{1}} \xi_{ik_{1}}\phi_{k_{1}}(s)+\sum_{k_{2}}^{N_{2}} \zeta_{\mathrm{ij}k_{2}}\psi_{k_{2}}(s)+\epsilon_{\mathrm{ij}}(s)$$

***Fast Multilevel FPCA***

Multilevel functional data has become increasingly prevalent. Multilevel FPCA (MLFPCA) extends the concept of measurement error and multilevel models to the case where functions are the fundamental unit of measurement, providing an explicit decomposition of both within-subject and between-subject variation in the function space.

As the number of observations per function increases, the computational of traditional MLFPCA becomes considerably slower. This slowdown arises from the need the computation of $O(\mathrm{IL}^{2})$ for constructing the sample covariance matrix $L\times L$. The standard bivariate smoothing, eigenanalysis, and MLFPC score estimations of the sample covariance matrix $L\times L$ need the computation of $O(L^{3})$. Fast MLFPCA alleviates the computational burden associated with the traditional MLFPCA.

The detailed steps are as follows:

(1) Estimate the overall mean function $\hat{\mu}(s)$ and the mean shift specific to the j-th observation $\hat{\eta}_{j}(s)$.

(2) Apply fast covariance estimation (FACE) to the total covariance $K_{T}(s,t)$ of the transformed functional data $\{\sqrt{nw_{i}}\tilde{Y}_{\mathrm{ij}},1\leq j\leq J_{i},1\leq i\leq I\}$.

(3) Apply FACE to the within-subject covariance $K_{W}(s,t)$ of the transformed functional data $\{\sqrt{nv_{i}J_{i}}(\tilde{Y}_{\mathrm{ij}}-\bar{Y}_{i\cdot}),1\leq j\leq J_{i},1\leq i\leq I\}$ to extract the within-subject eigenfunctions/eigenvalues.

(4) Calculate the between-subject covariance $K_{B}(s,t)$ based on the difference between the total covariance $K_{T}(s,t)$ and the within-subject covariance $K_{W}(s,t)$, and extract the between-subject eigenfunctions/eigenvalues.

(5) Estimate the error variance $\sigma^{2}$, $\hat{\sigma}^{2}=\int_{S} \{\hat{K}_{T}(s,s)-\tilde{K}_{T}(s,s)\}ds$.

(6) Estimate the fMLFPC scores based on the mixed model equations.

Methodological details are as follows:

(1) FACE and eigenanalysis

Set $Y$ be a data matrix $L\times I$ at different time points. The estimator of the sample covariance matrix is $\hat{K}=I^{-1}YY^{T}$. The smooth matrix $L\times L$ is constructed as $S=B{(B^{T}B/L+\lambda P)}^{-1}B^{T}/L$, where $B$ is the cubic B-spline basis, $P$ is the $q-\mathrm{th}$ order penalty matrix of the spline curve, and $\lambda$ is the smoothing parameter. $\tilde{K}=S\hat{K}S$ is used as a smooth estimator of covariance using FACE, $\tilde{K}(s,t)=B^{T}(s)\Theta B(t)$.

$$G=\int B(s)B^{T}(s)ds$$

Take the eigendecomposition $G^{1/2}\Theta G^{1/2}=U\Lambda U^{T}$ as an example, where $U=[U_{1},\cdots,U_{c}]$ is orthonormal matrix, $\Lambda=diag(\lambda_{1},\cdots,\lambda_{c})$ is diagonal matrix. $U_{k}^{T}G^{1/2}B(s)$ is the k-th eigenfunction corresponding to the eigenvalue $\lambda_{k}$.

(2) Transformed functional data

Let $\tilde{Y}_{\mathrm{ij}}(s)=Y_{\mathrm{ij}}(s)-\mu(s)-\eta_{j}(s)$ be demeaned data, where both the population mean function $\mu(s)$ and j-th observespecific mean shifts $\eta_{j}(s)$ are replaced by estimates.

$$\tilde{Y}_{\mathrm{ij}}={[\tilde{Y}_{\mathrm{ij}}(s_{1}),\cdots,\tilde{Y}_{\mathrm{ij}}(s_{L})]}^{T}$$

$$\tilde{Y}=[\tilde{Y}_{11},\cdots,\tilde{Y}_{1J_{1}},\cdots,\tilde{Y}_{I1},\cdots,\tilde{Y}_{IJ_{I}}]\in{\mathbb{\mathbb{R}}}^{L\times n}$$

where $n=\sum_{i=1}^{I} J_{i}$ is the total number of curves, $n_{I}=\sum_{i=1}^{I} J_{i}(J_{i}-1)$。

Let the between-subject covariance be$\hat{K}_{B}={n_{I}}^{-1}\tilde{Y}H_{B}\tilde{Y}^{T}$, where $H_{B}=blockdiag(1_{J_{1}}1_{J_{1}}^{T}-I_{J_{1}},\cdots,1_{J_{I}}1_{J_{I}}^{T}-I_{J_{I}})$.

MoM estimator of the total covariance $K_{T}(s,t)$ is $\hat{K}_{T}(s,t)=\sum_{i=1}^{n} \sum_{j=1}^{J_{i}} w_{i}\tilde{Y}_{\mathrm{ij}}(s)\tilde{Y}_{\mathrm{ij}}(t)$. The within-subject covariance is $K_{W}(s,t)=E\{\tilde{Y}_{\mathrm{ij}}(s)-\tilde{Y}_{\mathrm{ik}}(s)\}{\{\tilde{Y}_{\mathrm{ij}}(t)-\tilde{Y}_{\mathrm{ik}}(t)\}}^{T}/2$. The within-subject covariance estimator of the transformed functional data $\{\sqrt{nv_{i}J_{i}}(\tilde{Y}_{\mathrm{ij}}-\bar{Y}_{i\cdot}),1\leq j\leq J_{i},1\leq i\leq I\}$ is $\hat{K}_{W}=\sum_{i=1}^{I} \sum_{j=1}^{J_{i}} v_{i}J_{i}(\tilde{Y}_{\mathrm{ij}}-\bar{Y}_{i\cdot}){(\tilde{Y}_{\mathrm{ij}}-\bar{Y}_{i\cdot})}^{T}$.

(3) Multilevel FACE

Set the smooth estimates of $K_{T}(s,t)$ is $\tilde{K}_{T}(s,t)=B^{T}(s)\Theta_{T}B(t)$, where $\Theta_{T}$ and $\Theta_{W}$ are the semidefinite matrices of FACE. Smoothed estimates of between-subject covariance $K_{B}(s,t)$ is $\tilde{K}_{B}(s,t)={B(s)}^{T}\Theta_{B}B(t)$, where $\Theta_{B}=\Theta_{T}-\Theta_{W}$. To ensure that $\tilde{K}_{B}(s,t)$ is semi-definite, the eigenvectors associated with negative eigenvalues are discarded by eigendecomposition of $\Theta_{B}$.

(4) Score Prediction via Mixed Model Equations

Assuming that the level-1 eigenfunction $\phi_{k}(s)$ and level-2 eigenfunction $\psi_{k}(s)$ are known, define a multilevel functional model:

$$\tilde{Y}_{\mathrm{ij}}(s)=\sum_{k_{1}\geq1} \xi_{ik_{1}}\phi_{k_{1}}(s)+\sum_{k_{2}\geq1} \zeta_{\mathrm{ij}k_{2}}\psi_{k_{2}}(s)+\epsilon_{\mathrm{ij}}(s)$$

With multilevel FACE, the model can be approximated as follows:

$$\tilde{Y}_{\mathrm{ij}}(s)=\sum_{k_{1}=1}^{N_{1}} \xi_{ik_{1}}\phi_{k_{1}}(s)+\sum_{k_{2}=1}^{N_{2}} \zeta_{\mathrm{ij}k_{2}}\psi_{k_{2}}(s)+\epsilon_{\mathrm{ij}}(s)$$

The matrix form of the mixed effects model is

$$\tilde{Y}_{i}=\Phi_{i}\xi_{i}+\Psi_{i}\zeta_{i}+\epsilon_{i}$$

Based on the mixed effects model equation, the FPC score is estimated to be

$$\left( \begin{matrix} \hat{\xi}_{i} \\ \hat{\zeta}_{i} \end{matrix} \right)=\left( \begin{matrix} \Phi_{i}^{T}\Phi_{i}+\sigma^{2}\Lambda_{1}^{-1} & \Phi_{i}^{T}\Psi_{i} \\ \Psi_{i}^{T}\Phi_{i} & \Psi_{i}^{T}\Psi_{i}+\sigma^{2}I_{J_{i}}\bigotimes\Lambda_{2}^{-1} \end{matrix} \right)^{-1}\left( \begin{matrix} \Phi_{i}^{T}\tilde{Y}_{i} \\ \Psi_{i}^{T}\tilde{Y}_{i} \end{matrix} \right)$$

where, $\Lambda_{1}$ is the covariance matrix of $\xi_{i}$ and $\Lambda_{2}$ is the covariance matrix of $\zeta_{\mathrm{ij}}$.

***Reference***

[1] Cui E, Li R, Crainiceanu CM, Xiao L. Fast multilevel functional principal component analysis. *J Comput Graph Stat*. 2023; 32(2): 366-377.

[2] Weaver C, Xiao L, Lu W. Functional data analysis for longitudinal data with informative observation times. *Biometrics*. 2023; 79(2): 722-733.

[3] Shamshoian J, Şentürk D, Jeste S, Telesca D. Bayesian analysis of longitudinal and multidimensional functional data. *Biostatistics*. 2022; 23(2): 558-573.

[4] Shi H, Dong J, Wang L, Cao J. Functional principal component analysis for longitudinal data with informative dropout. *Stat Med*. 2021; 40(3): 712-724.

[5] Xiao L, Zipunnikov V, Ruppert D, Crainiceanu C. Fast covariance estimation for high-dimensional functional data. *Stat Comput*. 2016; 26(1): 409-421.

[6] Chiou JM, Müller HG. A pairwise interaction model for multivariate functional and longitudinal data. *Biometrika*. 2016; 103(2): 377-396.

[7] Di C, Crainiceanu CM, Jank WS. Multilevel sparse functional principal component analysis. *Stat*. 2014; 3(1): 126-143.

[8] Cuevas, Antonio. A partial overview of the theory of statistics with functional data. *J Stat Plan Inference*. 2014; 147: 1-23.

[9] Ullah S, Finch CF. Applications of functional data analysis: a systematic review. *BMC Med. Res. Methodol*. 2013; 13: 43.

[10] Di CZ, Crainiceanu CM, Caffo BS, Punjabi NM. Multilevel functional principal component analysis. *Ann Appl Stat*. 2009; 3(1): 458-488.

**RESULTS**

**Table 1 Baseline characteristics of all participants in this study.**

This study included 1,019 individuals with MCI, with 606 males (59.47%) and 413 females (40.53%); 805 were married (79.00%) and 214 were single (21.00%); the average age was 73.55 years, and the average years of education was 15.99 years. Among them, 517 individuals (50.74%) did not carry the APOEε4 allele, 396 (38.86%) carried one APOEε4 allele, and 106 (10.40%) carried two APOEε4 alleles.

And, there were 93 with rMCI, 567 with sMCI, and 359 with pMCI.

For rMCI, there were 53 males (56.99%) and 40 females (43.01%); 77 were married (82.80%) and 16 were single (17.20%); the baseline average age was 69.75 years, and the average years of education was 16.72 years. Among them, 59 individuals (63.44%) did not carry the APOEε4 allele, 31 (33.33%) carried one APOEε4 allele, and 3 (3.23%) carried two APOEε4 alleles.

For sMCI, there were 341 males (60.14%) and 226 females (39.86%); 438 were married (77.25%) and 129 were single (22.75%); the average baseline age was 73.85 years, and the average years of education was 15.91 years. Among them, 329 individuals (58.02%) did not carry the APOEε4 allele, 185 (32.63%) carried one APOEε4 allele, and 53 (9.35%) carried two APOEε4 alleles.

For pMCI, there were 212 males (59.05%) and 147 females (40.95%); 290 were married (80.78%) and 69 were single (19.22%); the average baseline age was 74.06 years, and the average years of education was 15.92 years. Among them, 129 individuals (35.93%) did not carry the APOEε4 allele, 180 (50.14%) carried one APOEε4 allele, and 50 (13.93%) carried two APOEε4 alleles.

Both the age and years of education of sMCI and pMCI individuals were higher than those of rMCI individuals. The proportions of pMCI individuals who did not carry the APOEε4 allele and those who carried one APOEε4 allele were higher than those of rMCI and sMCI individuals; the proportion of pMCI individuals who carried two APOEε4 alleles was higher than that of rMCI individuals.

| Variables | Total population (N=1019) | Outcome types of MCI | | | *χ^2^/F/H* | *P* |
| --- | --- | --- | --- | --- | --- | --- |
|  |  | rMCI (N=93) | sMCI (N=567) | pMCI (N=359) |  |  |
| Age, years | 73.55±7.55 | 69.75±8.13 | 73.85±7.63^a^ | 74.06±7.00^a^ | 13.382 | <0.001 |
| Sex |  |  |  |  |  |  |
| Female | 606 (59.47) | 53 (56.99) | 341 (60.14) | 212 (59.05) | 0.369 | 0.831 |
| Male | 413 (40.53) | 40 (43.01) | 226 (39.86) | 147 (40.95) |  |  |
| Educational attainment, years | 15.99±2.77 | 16.72±2.31 | 15.91±2.87^a^ | 15.92±2.70^a^ | 3.606 | 0.028 |
| Marital status |  |  |  |  |  |  |
| Married | 805 (79.00) | 77 (82.80) | 438 (77.25) | 290 (80.78) | 2.541 | 0.281 |
| Single | 214 (21.00) | 16 (17.20) | 129 (22.75) | 69 (19.22) |  |  |
| APOEε4 |  |  |  |  |  |  |
| -/- | 517 (50.74) | 59 (63.44) | 329 (58.02) | 129 (35.93)^ab^ | 52.051 | <0.001 |
| +/- | 396 (38.86) | 31 (33.33) | 185 (32.63) | 180 (50.14)^ab^ |  |  |
| +/+ | 106 (10.40) | 3 (3.23) | 53 (9.35) | 50 (13.93)^a^ |  |  |
| CDRSB | 1.5 (1, 2) | 1 (0.5, 1.5) | 1 (0.5, 1.5)^a^ | 1.5 (1, 2.5)^ab^ | 124.753 | <0.001 |
| MMSE | 28 (26, 29) | 29 (28, 30) | 28 (27, 29)^a^ | 27 (26, 28)^ab^ | 86.802 | <0.001 |
| ADAS11 | 7 (9.67, 13) | 6 (4.33, 9) | 9 (6, 11.67)^a^ | 12 (9.33, 15.33)^ab^ | 190.674 | <0.001 |
| ADAS13 | 16 (11, 21) | 10 (7, 13.50) | 14 (10.33, 19)^a^ | 20 (16, 24)^ab^ | 241.425 | <0.001 |
| ADASQ4 | 5 (4, 7) | 3 (2, 5) | 5 (3, 7)^a^ | 7 (5, 9)^ab^ | 205.175 | <0.001 |
| FAQ | 1 (0, 5) | 0 (0, 1) | 1 (0, 3)^a^ | 4 (1, 8)^ab^ | 169.913 | <0.001 |
| RAVLT Immediate Recall | 33 (27, 41) | 42 (34.50, 50) | 35 (29, 43)^a^ | 29 (24, 34)^ab^ | 160.812 | <0.001 |
| RAVLT Learning Across Trials | 4 (2, 6) | 5 (4, 7) | 4 (3, 6)^a^ | 3 (2, 4)^ab^ | 98.789 | <0.001 |
| RAVLT Forgetting | 5 (3, 6) | 5 (2, 6) | 4 (3, 6) | 5 (4, 6)^b^ | 12.711 | <0.001 |
| RAVLT Percentage of Forgetting | 61.54  (36.36, 92.31) | 36.36  (20, 65.16) | 54.55  (30.77, 80)^a^ | 83.33  (57.14, 100)^ab^ | 123.827 | <0.001 |
| TMT-B | 96  (70, 135) | 70  (58, 86) | 94  (69, 128)^a^ | 109  (78, 161)^ab^ | 77.414 | <0.001 |
| mPACCdigit | -5.85  (-8.98, -2.83) | -1.96  (-4.21, -0.81) | -4.93  (-7.70, -2.31)^a^ | -8.66  (-10.72, -5.45)^ab^ | 206.859 | <0.001 |
| mPACCtrailsB | -5.36  (-8.45, -2.60) | -1.41  (-3.12, -0.50) | -4.40  (-7.35, -2.28)^a^ | -8.12  (-10.55, -5.25)^ab^ | 230.954 | <0.001 |

MCI, mild cognitive impairment; rMCI, recurrent cognitive reversion; sMCI, stable cognitive status; pMCI, eventual progression to AD; CDRSB, Sum of Boxes score of the Clinical Dementia Rating Scale; MMSE, Mini Mental State Examination; ADAS, Alzheimer’s Disease Assessment Scale-cognitive subscale; FAQ, Functional Activities Questionnaire; RAVLT, Rey Auditory Verbal Learning Test.

For sex, marital status and APOEε4 carrier status, frequency (%) was used to describe, and Chi-square test was used to compare different MCI outcome types.

For age and educational attainment, mean±standard deviation was used to describe, ANOVA was used to compare different MCI outcome types, and LSD was used to make multiple comparisons.

For CDRSB, MMSE and other 13 cognitive assessments, P50 (P25, P75) was used to describe, Kruskal-Wallis test was used to compare different outcome types, and Bonferroni method was used to make multiple comparisons.

a, compared to rMCI, *P*<0.05；b, compared to sMCI, *P*<0.05.

**Table 2 Eigenvalue, proportion of variance explained (PVE) and accumulate PVE of the FPCs extracted for each longitudinal cognitive assessment using univariate FPCA, when setting the cumulative PVE at 90%.**

| Cognitive assessments | FPC1 | FPC2 | FPC3 |
| --- | --- | --- | --- |
| ADAS11.FPC |  |  |  |
| Eigenvalue | 12.745 | 4.735 | 2.218 |
| PVE (%) | 58.23 | 21.63 | 10.13 |
| Accumulate PVE (%) | 58.23 | 79.86 | 89.99 |
| ADAS13.FPC |  |  |  |
| Eigenvalue | 33.693 | 8.256 |  |
| PVE (%) | 72.29 | 17.71 |  |
| Accumulate PVE (%) | 72.29 | 90 |  |
| ADASQ4.FPC |  |  |  |
| Eigenvalue | 4.101 | 0.564 |  |
| PVE (%) | 79.11 | 10.89 |  |
| Accumulate PVE (%) | 79.11 | 90 |  |
| FAQ.FPC |  |  |  |
| Eigenvalue | 9.284 | 4.343 | 1.062 |
| PVE (%) | 56.88 | 26.61 | 6.51 |
| Accumulate PVE (%) | 56.88 | 83.49 | 90 |
| RAVLT Immediate Recall.FPC |  |  |  |
| Eigenvalue | 104.421 | 6.938 |  |
| PVE (%) | 84.39 | 5.61 |  |
| Accumulate PVE (%) | 84.39 | 90 |  |
| RAVLT Learning Across Trials.FPC |  |  |  |
| Eigenvalue | 2.787 | 0.297 |  |
| PVE (%) | 81.33 | 8.67 |  |
| Accumulate PVE (%) | 81.33 | 90 |  |
| RAVLT Forgetting.FPC |  |  |  |
| Eigenvalue | 2.490 | 0.517 | 0.280 |
| PVE (%) | 68.18 | 14.16 | 7.66 |
| Accumulate PVE (%) | 68.18 | 82.34 | 90 |
| RAVLT Percentage of Forgetting.FPC |  |  |  |
| Eigenvalue | 608.778 | 137.140 |  |
| PVE (%) | 73.45 | 16.55 |  |
| Accumulate PVE (%) | 73.45 | 90 |  |
| TMT-B.FPC |  |  |  |
| Eigenvalue | 2428.984 | 722.696 |  |
| PVE (%) | 69.36 | 20.64 |  |
| Accumulate PVE (%) | 69.36 | 90 |  |
| mPACCdigit.FPC |  |  |  |
| Eigenvalue | 19.139 | 4.717 |  |
| PVE (%) | 72.21 | 17.79 |  |
| Accumulate PVE (%) | 72.21 | 90 |  |
| mPACCtrailsB.FPC |  |  |  |
| Eigenvalue | 17.402 | 4.142 |  |
| PVE (%) | 72.70 | 17.30 |  |
| Accumulate PVE (%) | 72.7 | 90 |  |

**Table 3 Spatial structure order and related information of different brain regions.**

| Spatial structure order | NO.AAL | Brain region | Anatomical structure | Location | Spatial structure order | NO.AAL | Brain region | Anatomical structure | Location |
| --- | --- | --- | --- | --- | --- | --- | --- | --- | --- |
| 1 | 29 | Insula | Insula | Left hemisphere | 59 | 113 | Vermis | Vermis | Vermis |
| 2 | 31 | Anterior cingulate and paracingulate gyri | Limbic lobe | Left hemisphere | 60 | 114 | Vermis | Vermis | Vermis |
| 3 | 33 | Median cingulate and paracingulate gyri | Limbic lobe | Left hemisphere | 61 | 115 | Vermis | Vermis | Vermis |
| 4 | 35 | Posterior cingulate gyrus | Limbic lobe | Left hemisphere | 62 | 116 | Vermis | Vermis | Vermis |
| 5 | 37 | Hippocampus | Limbic lobe | Left hemisphere | 63 | 108 | Cerebelum | Cerebellum | Right cerebellum |
| 6 | 39 | Parahippocampal gyrus | Limbic lobe | Left hemisphere | 64 | 106 | Cerebelum | Cerebellum | Right cerebellum |
| 7 | 83 | Temporal pole: superior temporal gyrus | Limbic lobe | Left hemisphere | 65 | 104 | Cerebelum | Cerebellum | Right cerebellum |
| 8 | 87 | Temporal pole: middle temporal gyrus | Limbic lobe | Left hemisphere | 66 | 102 | Cerebelum | Cerebellum | Right cerebellum |
| 9 | 41 | Amygdala | Subcortical gray nuclei | Left hemisphere | 67 | 100 | Cerebelum | Cerebellum | Right cerebellum |
| 10 | 71 | Caudate nucleus | Subcortical gray nuclei | Left hemisphere | 68 | 98 | Cerebelum | Cerebellum | Right cerebellum |
| 11 | 73 | Lenticular nucleus, putamen | Subcortical gray nuclei | Left hemisphere | 69 | 96 | Cerebelum | Cerebellum | Right cerebellum |
| 12 | 75 | Lenticular nucleus, pallidum | Subcortical gray nuclei | Left hemisphere | 70 | 94 | Cerebelum_Crus | Cerebellum | Right cerebellum |
| 13 | 77 | Thalamus | Subcortical gray nuclei | Left hemisphere | 71 | 92 | Cerebelum_Crus | Cerebellum | Right cerebellum |
| 14 | 3 | Superior frontal gyrus dorsolateral | Frontal lobe | Left hemisphere | 72 | 90 | Inferior temporal gyrus | Temporal lobe | Right hemisphere |
| 15 | 7 | Middle frontal gyrus | Frontal lobe | Left hemisphere | 73 | 86 | Middle temporal gyrus | Temporal lobe | Right hemisphere |
| 16 | 11 | Inferior frontal gyrus, opercular part | Frontal lobe | Left hemisphere | 74 | 82 | Superior temporal gyrus | Temporal lobe | Right hemisphere |
| 17 | 13 | Inferior frontal gyrus, triangular part | Frontal lobe | Left hemisphere | 75 | 80 | Heschl gyrus | Temporal lobe | Right hemisphere |
| 18 | 19 | Supplementary motor area | Frontal lobe | Left hemisphere | 76 | 56 | Fusiform gyrus | Occipital lobe | Right hemisphere |
| 19 | 23 | Superior frontal gyrus, medial | Frontal lobe | Left hemisphere | 77 | 48 | Lingual gyrus | Occipital lobe | Right hemisphere |
| 20 | 69 | Paracentral lobule | Frontal lobe | Left hemisphere | 78 | 46 | Cuneus | Occipital lobe | Right hemisphere |
| 21 | 5 | Superior frontal gyrus orbital part | Frontal lobe | Left hemisphere | 79 | 44 | Calcarine fissure and surrounding cortex | Occipital lobe | Right hemisphere |
| 22 | 9 | Middle frontal gyrus, orbital part | Frontal lobe | Left hemisphere | 80 | 54 | Inferior occipital gyrus | Occipital lobe | Right hemisphere |
| 23 | 15 | Inferior frontal gyrus, orbital part | Frontal lobe | Left hemisphere | 81 | 52 | Middle occipital gyrus | Occipital lobe | Right hemisphere |
| 24 | 21 | Olfactory cortex | Frontal lobe | Left hemisphere | 82 | 50 | Superior occipital gyrus | Occipital lobe | Right hemisphere |
| 25 | 25 | Superior frontal gyrus, medial orbital | Frontal lobe | Left hemisphere | 83 | 68 | Precuneus | Parietal lobe | Right hemisphere |
| 26 | 27 | Gyrus rectus | Frontal lobe | Left hemisphere | 84 | 66 | Angular gyrus | Parietal lobe | Right hemisphere |
| 27 | 1 | Percental gyrus | Central region | Left hemisphere | 85 | 64 | Supramarginal gyrus | Parietal lobe | Right hemisphere |
| 28 | 17 | Rolandic operculum | Central region | Left hemisphere | 86 | 62 | Inferior parietal, but supramarginal and angular gyri | Parietal lobe | Right hemisphere |
| 29 | 57 | Postcentral gyrus | Central region | Left hemisphere | 87 | 60 | Superior parietal gyrus | Parietal lobe | Right hemisphere |
| 30 | 59 | Superior parietal gyrus | Parietal lobe | Left hemisphere | 88 | 58 | Postcentral gyrus | Central region | Right hemisphere |
| 31 | 61 | Inferior parietal, but supramarginal and angular gyri | Parietal lobe | Left hemisphere | 89 | 18 | Rolandic operculum | Central region | Right hemisphere |
| 32 | 63 | Supramarginal gyrus | Parietal lobe | Left hemisphere | 90 | 2 | Percental gyrus | Central region | Right hemisphere |
| 33 | 65 | Angular gyrus | Parietal lobe | Left hemisphere | 91 | 28 | Gyrus rectus | Frontal lobe | Right hemisphere |
| 34 | 67 | Precuneus | Parietal lobe | Left hemisphere | 92 | 26 | Superior frontal gyrus, medial orbital | Frontal lobe | Right hemisphere |
| 35 | 49 | Superior occipital gyrus | Occipital lobe | Left hemisphere | 93 | 22 | Olfactory cortex | Frontal lobe | Right hemisphere |
| 36 | 51 | Middle occipital gyrus | Occipital lobe | Left hemisphere | 94 | 16 | Inferior frontal gyrus,orbital part | Frontal lobe | Right hemisphere |
| 37 | 53 | Inferior occipital gyrus | Occipital lobe | Left hemisphere | 95 | 10 | Middle frontal gyrus, orbital part | Frontal lobe | Right hemisphere |
| 38 | 43 | Calcarine fissure and surrounding cortex | Occipital lobe | Left hemisphere | 96 | 6 | Superior frontal gyrus orbital part | Frontal lobe | Right hemisphere |
| 39 | 45 | Cuneus | Occipital lobe | Left hemisphere | 97 | 70 | Paracentral lobule | Frontal lobe | Right hemisphere |
| 40 | 47 | Lingual gyrus | Occipital lobe | Left hemisphere | 98 | 24 | Superior frontal gyrus, medial | Frontal lobe | Right hemisphere |
| 41 | 55 | Fusiform gyrus | Occipital lobe | Left hemisphere | 99 | 20 | Supplementary motor area | Frontal lobe | Right hemisphere |
| 42 | 79 | Heschl gyrus | Temporal lobe | Left hemisphere | 100 | 14 | Inferior frontal gyrus, triangular part | Frontal lobe | Right hemisphere |
| 43 | 81 | Superior temporal gyrus | Temporal lobe | Left hemisphere | 101 | 12 | Inferior frontal gyrus, opercular part | Frontal lobe | Right hemisphere |
| 44 | 85 | Middle temporal gyrus | Temporal lobe | Left hemisphere | 102 | 8 | Middle frontal gyrus | Frontal lobe | Right hemisphere |
| 45 | 89 | Inferior temporal gyrus | Temporal lobe | Left hemisphere | 103 | 4 | Superior frontal gyrus dorsolateral | Frontal lobe | Right hemisphere |
| 46 | 91 | Cerebelum_Crus | Cerebellum | Left cerebellum | 104 | 78 | Thalamus | Subcortical gray nuclei | Right hemisphere |
| 47 | 93 | Cerebelum_Crus | Cerebellum | Left cerebellum | 105 | 76 | Lenticular nucleus, pallidum | Subcortical gray nuclei | Right hemisphere |
| 48 | 95 | Cerebelum | Cerebellum | Left cerebellum | 106 | 74 | Lenticular nucleus, putamen | Subcortical gray nuclei | Right hemisphere |
| 49 | 97 | Cerebelum | Cerebellum | Left cerebellum | 107 | 72 | Caudate nucleus | Subcortical gray nuclei | Right hemisphere |
| 50 | 99 | Cerebelum | Cerebellum | Left cerebellum | 108 | 42 | Amygdala | Subcortical gray nuclei | Right hemisphere |
| 51 | 101 | Cerebelum | Cerebellum | Left cerebellum | 109 | 88 | Temporal pole: middle temporal gyrus | Limbic lobe | Right hemisphere |
| 52 | 103 | Cerebelum | Cerebellum | Left cerebellum | 110 | 84 | Temporal pole: superior temporal gyrus | Limbic lobe | Right hemisphere |
| 53 | 105 | Cerebelum | Cerebellum | Left cerebellum | 111 | 40 | Parahippocampal gyrus | Limbic lobe | Right hemisphere |
| 54 | 107 | Cerebelum | Cerebellum | Left cerebellum | 112 | 38 | Hippocampus | Limbic lobe | Right hemisphere |
| 55 | 109 | Vermis | Vermis | Vermis | 113 | 36 | Posterior cingulate gyrus | Limbic lobe | Right hemisphere |
| 56 | 110 | Vermis | Vermis | Vermis | 114 | 34 | Median cingulate and paracingulate gyri | Limbic lobe | Right hemisphere |
| 57 | 111 | Vermis | Vermis | Vermis | 115 | 32 | Anterior cingulate and paracingulate gyri | Limbic lobe | Right hemisphere |
| 58 | 112 | Vermis | Vermis | Vermis | 116 | 30 | Insula | Insula | Right hemisphere |

**Table 4 Eigenvalue, PVE and accumulate PVE of the fiest ten FPCs extracted for gray volume proportions using univariate FPCA in the brain space continuous domain.**

| FPC | FPC1 | FPC2 | FPC3 | FPC4 | FPC5 | FPC6 | FPC7 | FPC8 | FPC9 | FPC10 |
| --- | --- | --- | --- | --- | --- | --- | --- | --- | --- | --- |
| Eigenvalue | 0.703 | 0.240 | 0.173 | 0.041 | 0.030 | 0.020 | 0.012 | 0.009 | 0.003 | 0.003 |
| PVE (%) | 56.48 | 19.26 | 13.92 | 3.33 | 2.39 | 1.65 | 0.96 | 0.74 | 0.27 | 0.24 |
| Accumulate PVE (%) | 56.48 | 75.74 | 89.66 | 92.99 | 95.38 | 97.03 | 97.99 | 98.73 | 99.00 | 99.24 |

**Table 5 Eigenvalue, PVE and accumulate PVE of the level 2 tMLFPCs for gray volume proportions using tMLFPCA.**

| tMLFPCs | Level 2.1 | Level 2.2 | Level 2.3 | Level 2.4 | Level 2.5 | Level 2.6 | Level 2.7 |
| --- | --- | --- | --- | --- | --- | --- | --- |
| Log_10_ of eigenvalue | -17.277 | -17.526 | -17.677 | -17.692 | -17.728 | -17.806 | -17.955 |
| PVE(%) | 3.40 | 3.45 | 3.48 | 3.48 | 3.49 | 3.50 | 3.53 |
| Accumulate PVE(%) | 3.40 | 6.85 | 10.33 | 13.81 | 17.30 | 20.80 | 24.33 |
| tMLFPCs | Level 2.8 | Level 2.9 | Level 2.10 | Level 2.11 | Level 2.12 | Level 2.13 | Level 2.14 |
| Log_10_ of eigenvalue | -17.997 | -18.024 | -18.102 | -18.123 | -18.145 | -18.183 | -18.224 |
| PVE(%) | 3.54 | 3.54 | 3.56 | 3.56 | 3.57 | 3.58 | 3.58 |
| Accumulate PVE(%) | 27.87 | 31.41 | 34.97 | 38.53 | 42.10 | 45.68 | 49.26 |
| tMLFPCs | Level 2.15 | Level 2.16 | Level 2.17 | Level 2.18 | Level 2.19 | Level 2.20 | Level 2.21 |
| Log_10_ of eigenvalue | -18.262 | -18.281 | -18.329 | -18.363 | -18.377 | -18.386 | -18.410 |
| PVE(%) | 3.59 | 3.59 | 3.60 | 3.61 | 3.61 | 3.62 | 3.62 |
| Accumulate PVE(%) | 52.85 | 56.44 | 60.04 | 63.65 | 67.26 | 70.88 | 74.50 |
| tMLFPCs | Level 2.22 | Level 2.23 | Level 2.24 | Level 2.25 | Level 2.26 | Level 2.27 | Level 2.28 |
| Log_10_ of eigenvalue | -18.460 | -18.473 | -18.495 | -18.527 | -18.546 | -18.560 | -18.592 |
| PVE(%) | 3.63 | 3.63 | 3.64 | 3.64 | 3.65 | 3.65 | 3.66 |
| Accumulate PVE(%) | 78.13 | 81.76 | 85.40 | 89.04 | 92.69 | 96.34 | 100 |

**Table 6 Eigenvalue, PVE and accumulate PVE of the level 2 fMLFPCs for gray volume proportions using fMLFPCA.**

| fMLFPCs | level 1 | level 2.1 | level 2.2 | level 2.3 |
| --- | --- | --- | --- | --- |
| Log_10_ of eigenvalue | -0.477 | -2.244 | -2.743 | -2.990 |
| PVE(%) | 100 | 66.83 | 21.18 | 11.99 |
| Accumulate PVE(%) | 100 | 66.83 | 88.01 | 100 |

**Table 7 -2*Ln* likelihood and AIC values of the FMSM models and MSM models constructed in this study.**

Compared with MSMs, FMSMs had lower -2Ln likelihood and AIC and better performance.

FMSM 4 added the FPC scores extracted from longitudinal neuroimaging in the spatial continuous domain on the basis of FMSM 1, and there was no significant difference in the performance of these two models (G=4.059, P=0.982).

FMSM 4 added the MVFPC scores extracted from longitudinal cognitive assessments on the basis of FMSM 2, and the performance of FMSM 4 was significantly improved (G=237.072, P<0.001)

FMSM 5 added the fMLFPC scores extracted from longitudinal neuroimaging in the spatial-temporal two-dimensional domain on the basis of FMSM 1, and there was no significant difference in the performance of these two models (G=8.616，P=0.735).

FMSM 5 added the MVFPC scores extracted from longitudinal cognitive assessments on the basis of FMSM 3, and the performance of FMSM 5 was significantly improved (G=234.270，P<0.001)

G = -2 (*ln*L_0_ - *ln*L_1_) = (-2 *ln*L_0_) - (-2 *ln*L_1_)

| Models | Longitudinal markers | *-2ln Likelihood* | AIC | *G^#^* |
| --- | --- | --- | --- | --- |
| MSM 0 | - | 3301.766 | 3307.766 | - |
| FMSM 1 | MVFPC scores derived from eleven longitudinal neuropsychological scales | 2944.705 | 2992.705 | 357.061^*^ |
| FMSM 2 | FPC scores derived from longitudinal neuroimaging data in the spatial domain | 3177.718 | 3237.718 | 124.048^*^ |
| FMSM 3 | fMLFPC scores derived from longitudinal neuroimaging data in the spatial-temporal two-dimensional domain | 3170.359 | 3230.359 | 131.407^*^ |
| FMSM 4 | FMSMs 1+2 | 2940.646 | 3012.646 | 361.120^*^ |
| FMSM 5 | FMSMs 1+3 | 2936.089 | 3008.089 | 365.677^*^ |
| MSM 1 | PC scores derived from the same eleven longitudinal neuropsychological scales | 3255.17 | 3285.17 | 46.596^*^ |
| MSM 2 | PC scores derived from longitudinal neuroimaging data | 3275.767 | 3311.767 | 25.999^*^ |
| MSM 3 | MSMs 1+2 | 3222.020 | 3276.02 | 79.746^*^ |

^#^, *G* values comparing with MSM 0.

^*^, *P*<0.05.

**Table 8 Tables of observed and expected number of transitions and percentages over a set of times were constructed, assuming that the cognitive status of individuals at any time was the same as at the previous observed time, and tested by Wilcoxon Signed-Rank Test.**

| Models | *W* | *z* | *P* |
| --- | --- | --- | --- |
| MSM 0 | 296 | 0.277 | 0.782 |
| FMSM 1 | 2390 | 0.227 | 0.821 |
| FMSM 2 | 2334 | 0.022 | 0.983 |
| FMSM 3 | 2394 | 0.241 | 0.809 |
| FMSM 4 | 2333 | 0.018 | 0.985 |
| FMSM 5 | 2345 | 0.062 | 0.950 |
| MSM 1 | 2411 | 0.303 | 0.762 |
| MSM 2 | 2396 | 0.248 | 0.804 |
| MSM 3 | 2250 | 0.247 | 0.805 |

**Table 9 Estimated transition probabilities and their 95% CIs in the FMSM 1, incorporated longitudinal cognitive information by including the MVFPC scores derived from the eleven cognitive assessments in the temporal continuous domain.**

| Years | MCI→rNC | MCI→MCI | MCI→AD | rNC→rNC | rNC→MCI | rNC→AD |
| --- | --- | --- | --- | --- | --- | --- |
| 1 | 0.018  (0.013, 0.026) | 0.899  (0.886, 0.910) | 0.083  (0.073, 0.094) | 0.873  (0.752, 0.934) | 0.122  (0.063, 0.236) | 0.006  (0.003, 0.011) |
| 2 | 0.032  (0.022, 0.045) | 0.810  (0.788, 0.831) | 0.158  (0.139, 0.179) | 0.764  (0.589, 0.870) | 0.216  (0.119, 0.375) | 0.021  (0.011, 0.037) |
| 3 | 0.042  (0.029, 0.059) | 0.732  (0.702, 0.759) | 0.225  (0.200, 0.253) | 0.670  (0.448, 0.816) | 0.287  (0.16, 0.477) | 0.043  (0.023, 0.076) |
| 4 | 0.050  (0.033, 0.071) | 0.664  (0.629, 0.696) | 0.286  (0.255, 0.321) | 0.590  (0.333, 0.768) | 0.340  (0.192, 0.538) | 0.070  (0.038, 0.126) |
| 5 | 0.056  (0.037, 0.078) | 0.603  (0.562, 0.641) | 0.342  (0.306, 0.379) | 0.521  (0.268, 0.714) | 0.377  (0.225, 0.563) | 0.102  (0.056, 0.174) |
| 6 | 0.060  (0.038, 0.085) | 0.549  (0.502, 0.593) | 0.392  (0.349, 0.434) | 0.461  (0.231, 0.670) | 0.403  (0.249, 0.558) | 0.136  (0.080, 0.217) |
| 7 | 0.062  (0.038, 0.090) | 0.500  (0.454, 0.547) | 0.438  (0.395, 0.487) | 0.410  (0.175, 0.630) | 0.418  (0.269, 0.559) | 0.172  (0.103, 0.274) |
| 8 | 0.063  (0.039, 0.093) | 0.457  (0.407, 0.504) | 0.480  (0.431, 0.528) | 0.365  (0.150, 0.599) | 0.426  (0.280, 0.541) | 0.209  (0.120, 0.311) |
| 9 | 0.063  (0.037, 0.095) | 0.419  (0.366, 0.467) | 0.518  (0.469, 0.569) | 0.326  (0.128, 0.551) | 0.427  (0.294, 0.523) | 0.246  (0.151, 0.360) |
| 10 | 0.063  (0.036, 0.096) | 0.384  (0.338, 0.431) | 0.553  (0.501, 0.605) | 0.293  (0.107, 0.537) | 0.424  (0.295, 0.506) | 0.284  (0.170, 0.405) |
| 11 | 0.062  (0.032, 0.098) | 0.353  (0.306, 0.403) | 0.585  (0.534, 0.635) | 0.263  (0.085, 0.498) | 0.417  (0.300, 0.486) | 0.320  (0.198, 0.451) |
| 12 | 0.060  (0.032, 0.096) | 0.325  (0.278, 0.376) | 0.615  (0.561, 0.665) | 0.237  (0.081, 0.464) | 0.407  (0.298, 0.464) | 0.356  (0.226, 0.485) |
| 13 | 0.058  (0.030, 0.095) | 0.299  (0.253, 0.348) | 0.642  (0.586, 0.696) | 0.214  (0.069, 0.455) | 0.394  (0.293, 0.443) | 0.392  (0.245, 0.531) |
| 14 | 0.056  (0.028, 0.095) | 0.276  (0.228, 0.325) | 0.667  (0.612, 0.721) | 0.194  (0.057, 0.420) | 0.381  (0.297, 0.424) | 0.425  (0.273, 0.565) |
| 15 | 0.054  (0.027, 0.091) | 0.255  (0.211, 0.304) | 0.691  (0.634, 0.741) | 0.176  (0.052, 0.399) | 0.366  (0.295, 0.406) | 0.458  (0.300, 0.600) |

**Table 10 Estimated total length of stay for post-reversion over 15 years in the FMSM 1, incorporated longitudinal cognitive information by including the MVFPC scores derived from the eleven cognitive assessments in the temporal continuous domain.**

| Years | Total time (years) | | | Percentage (%) | | |
| --- | --- | --- | --- | --- | --- | --- |
|  | rNC | MCI | AD | rNC | MCI | AD |
| Year 1 | 0.9346 | 0.0635 | 0.0019 | 93.46 | 6.35 | 0.19 |
| Year 2 | 1.7514 | 0.2344 | 0.0142 | 87.57 | 11.72 | 0.71 |
| Year 3 | 2.4672 | 0.4875 | 0.0453 | 82.24 | 16.25 | 1.51 |
| Year 4 | 3.0963 | 0.8023 | 0.1014 | 77.41 | 20.06 | 2.54 |
| Year 5 | 3.651 | 1.1619 | 0.1871 | 73.02 | 23.24 | 3.74 |
| Year 6 | 4.1415 | 1.5528 | 0.3057 | 69.03 | 25.88 | 5.10 |
| Year 7 | 4.5766 | 1.9639 | 0.4595 | 65.38 | 28.06 | 6.56 |
| Year 8 | 4.9636 | 2.3866 | 0.6498 | 62.05 | 29.83 | 8.12 |
| Year 9 | 5.309 | 2.8136 | 0.8774 | 58.99 | 31.26 | 9.75 |
| Year 10 | 5.6181 | 3.2396 | 1.1423 | 56.18 | 32.40 | 11.42 |
| Year 11 | 5.8954 | 3.6603 | 1.4443 | 53.59 | 33.28 | 13.13 |
| Year 12 | 6.145 | 4.0722 | 1.7828 | 51.21 | 33.94 | 14.86 |
| Year 13 | 6.3702 | 4.4729 | 2.1568 | 49.00 | 34.41 | 16.59 |
| Year 14 | 6.574 | 4.8606 | 2.5654 | 46.96 | 34.72 | 18.32 |
| Year 15 | 6.7588 | 5.234 | 3.0073 | 45.06 | 34.89 | 20.05 |

**Table 11 Estimated HRs and their 95% CIs in the FMSM 1, incorporated longitudinal cognitive information by including the MVFPC scores derived from the eleven cognitive assessments in the temporal continuous domain.**

| Covariates | HR | | |
| --- | --- | --- | --- |
|  | MCI→AD | MCI→rNC | rNC→MCI |
| Age, years | 1.010(0.995, 1.025) | 0.987(0.957, 1.017) | 0.993(0.941, 1.047) |
| Sex (female) | 1.247(0.993, 1.567) | 1.178(0.755, 1.837) | 1.069(0.483, 2.366) |
| Educational attainment, years | 1.054(1.016, 1.093)^*^ | 1.008(0.924, 1.100) | 0.841(0.705, 1.003) |
| Marital status (married) | 1.322(1.002, 1.743)^*^ | 1.189(0.682, 2.072) | 0.925(0.333, 2.567) |
| APOEε4 | 1.438(1.231, 1.681)^*^ | 1.128(0.810, 1.570) | 1.065(0.450, 2.519) |
| MVFPC1 | 1.003(1.002, 1.004)^*^ | 0.993(0.991, 0.995)^*^ | 1.000(0.996, 1.004) |
| MVFPC2 | 0.993(0.991, 1.994) | 1.004(1.001, 1.007)^*^ | 1.003(0.999, 1.008) |

**Table 12 The observed and expected numbers at one-yearly intervals up to 15 years in the FMSM 1, incorporated longitudinal neuroimaging data through FPC scores in the spatial domain.**

| Years | Observed | | | Expected | | |
| --- | --- | --- | --- | --- | --- | --- |
|  | rNC | MCI | AD | rNC | MCI | AD |
| 0 | 0 | 1019 | 0 | 0.000 | 1019.000 | 0.000 |
| 1 | 32 | 832 | 115 | 29.749 | 831.397 | 117.854 |
| 2 | 42 | 561 | 234 | 44.794 | 606.789 | 185.417 |
| 3 | 45 | 366 | 278 | 46.725 | 424.965 | 217.310 |
| 4 | 39 | 238 | 317 | 44.627 | 311.219 | 238.154 |
| 5 | 36 | 156 | 329 | 39.632 | 230.980 | 250.387 |
| 6 | 38 | 122 | 338 | 38.405 | 191.675 | 267.920 |
| 7 | 35 | 98 | 341 | 35.914 | 157.393 | 280.693 |
| 8 | 28 | 74 | 347 | 33.062 | 128.972 | 286.966 |
| 9 | 16 | 54 | 353 | 28.401 | 104.096 | 290.503 |
| 10 | 11 | 39 | 356 | 24.869 | 87.378 | 293.754 |
| 11 | 7 | 20 | 359 | 19.162 | 69.868 | 296.970 |
| 12 | 3 | 8 | 359 | 14.982 | 56.389 | 298.629 |
| 13 | 2 | 4 | 359 | 13.944 | 49.022 | 302.035 |
| 14 | 2 | 1 | 359 | 12.940 | 42.966 | 306.095 |
| 15 | 2 | 1 | 359 | 12.505 | 38.914 | 310.580 |

**Table 13 Estimated transition probabilities and their 95% CIs in the FMSM 2, incorporated longitudinal neuroimaging information by including FPC scores in the spatial continuous domain.**

| Years | MCI→rNC | MCI→MCI | MCI→AD | rNC→rNC | rNC→MCI | rNC→AD |
| --- | --- | --- | --- | --- | --- | --- |
| 1 | 0.030  (0.024, 0.037) | 0.866  (0.853, 0.879) | 0.105  (0.094, 0.116) | 0.887  (0.828, 0.927) | 0.107  (0.069, 0.162) | 0.006  (0.004, 0.010) |
| 2 | 0.052  (0.042, 0.064) | 0.753  (0.730, 0.773) | 0.195  (0.178, 0.216) | 0.790  (0.687, 0.865) | 0.187  (0.121, 0.279) | 0.023  (0.014, 0.035) |
| 3 | 0.068  (0.055, 0.086) | 0.657  (0.628, 0.683) | 0.274  (0.250, 0.302) | 0.706  (0.578, 0.802) | 0.247  (0.168, 0.351) | 0.048  (0.031, 0.072) |
| 4 | 0.080  (0.063, 0.101) | 0.576  (0.543, 0.607) | 0.343  (0.314, 0.374) | 0.633  (0.474, 0.746) | 0.289  (0.200, 0.411) | 0.078  (0.052, 0.119) |
| 5 | 0.088  (0.068, 0.110) | 0.508  (0.475, 0.541) | 0.404  (0.370, 0.438) | 0.570  (0.428, 0.708) | 0.318  (0.219, 0.417) | 0.112  (0.073, 0.158) |
| 6 | 0.093  (0.070, 0.119) | 0.449  (0.414, 0.485) | 0.458  (0.422, 0.495) | 0.515  (0.341, 0.663) | 0.336  (0.237, 0.449) | 0.149  (0.099, 0.216) |
| 7 | 0.096  (0.072, 0.122) | 0.399  (0.363, 0.436) | 0.505  (0.470, 0.544) | 0.467  (0.302, 0.614) | 0.346  (0.254, 0.439) | 0.187  (0.128, 0.259) |
| 8 | 0.097  (0.070, 0.121) | 0.355  (0.318, 0.393) | 0.548  (0.513, 0.587) | 0.424  (0.254, 0.584) | 0.350  (0.256, 0.437) | 0.226  (0.158, 0.306) |
| 9 | 0.097  (0.069, 0.128) | 0.318  (0.279, 0.357) | 0.585  (0.545, 0.626) | 0.387  (0.223, 0.54) | 0.348  (0.267, 0.421) | 0.265  (0.186, 0.362) |
| 10 | 0.095  (0.068, 0.130) | 0.286  (0.251, 0.321) | 0.619  (0.575, 0.661) | 0.353  (0.217, 0.534) | 0.343  (0.260, 0.400) | 0.304  (0.205, 0.397) |
| 11 | 0.093  (0.063, 0.127) | 0.258  (0.220, 0.296) | 0.650  (0.608, 0.694) | 0.323  (0.174, 0.492) | 0.334  (0.258, 0.386) | 0.342  (0.243, 0.452) |
| 12 | 0.090  (0.060, 0.122) | 0.233  (0.199, 0.268) | 0.677  (0.635, 0.720) | 0.297  (0.155, 0.455) | 0.324  (0.260, 0.368) | 0.379  (0.279, 0.485) |
| 13 | 0.087  (0.058, 0.123) | 0.211  (0.176, 0.248) | 0.702  (0.659, 0.739) | 0.273  (0.140, 0.435) | 0.312  (0.255, 0.352) | 0.415  (0.305, 0.525) |
| 14 | 0.083  (0.053, 0.118) | 0.192  (0.161, 0.224) | 0.725  (0.684, 0.765) | 0.251  (0.125, 0.414) | 0.300  (0.250, 0.331) | 0.449  (0.334, 0.566) |
| 15 | 0.079  (0.048, 0.113) | 0.175  (0.145, 0.206) | 0.745  (0.703, 0.785) | 0.232  (0.108, 0.374) | 0.286  (0.243, 0.315) | 0.482  (0.370, 0.606) |

**Table 14 Estimated total length of stay for post-reversion over 15 years in the FMSM 2, incorporated longitudinal neuroimaging information by including FPC scores in the spatial continuous domain.**

| Years | Total time (years) | | | Percentage (%) | | |
| --- | --- | --- | --- | --- | --- | --- |
|  | rNC | MCI | AD | rNC | MCI | AD |
| Year 1 | 0.9420 | 0.0559 | 0.0021 | 94.20 | 5.59 | 0.21 |
| Year 2 | 1.7790 | 0.2049 | 0.0160 | 88.95 | 10.25 | 0.80 |
| Year 3 | 2.5258 | 0.4234 | 0.0507 | 84.19 | 14.11 | 1.69 |
| Year 4 | 3.1946 | 0.6924 | 0.1130 | 79.87 | 17.31 | 2.82 |
| Year 5 | 3.7957 | 0.9967 | 0.2075 | 75.91 | 19.93 | 4.15 |
| Year 6 | 4.3378 | 1.3245 | 0.3377 | 72.30 | 22.07 | 5.63 |
| Year 7 | 4.8283 | 1.6662 | 0.5055 | 68.98 | 23.80 | 7.22 |
| Year 8 | 5.2735 | 2.0145 | 0.7120 | 65.92 | 25.18 | 8.90 |
| Year 9 | 5.6786 | 2.3636 | 0.9578 | 63.10 | 26.26 | 10.64 |
| Year 10 | 6.0483 | 2.7091 | 1.2426 | 60.48 | 27.09 | 12.43 |
| Year 11 | 6.3863 | 3.0478 | 1.5659 | 58.06 | 27.71 | 14.24 |
| Year 12 | 6.6962 | 3.3772 | 1.9266 | 55.80 | 28.14 | 16.06 |
| Year 13 | 6.9808 | 3.6954 | 2.3238 | 53.70 | 28.43 | 17.88 |
| Year 14 | 7.2426 | 4.0014 | 2.7560 | 51.73 | 28.58 | 19.69 |
| Year 15 | 7.4839 | 4.2943 | 3.2218 | 49.89 | 28.63 | 21.48 |

**Table 15 Estimated HRs and their 95% CIs in the FMSM 2, incorporated longitudinal neuroimaging information by including FPC scores in the spatial continuous domain.**

| Covariates | HR | | |
| --- | --- | --- | --- |
|  | MCI→AD | MCI→rNC | rNC→MCI |
| Age, years | 1.030(1.015, 1.046)^*^ | 0.939(0.914, 0.963)^*^ | 0.987(0.935, 1.043) |
| Sex (female) | 1.259(0.994, 1.595) | 0.974(0.633, 1.498) | 0.647(0.283, 1.482) |
| Educational attainment, years | 1.009(0.971, 1.047) | 1.064(0.981, 1.154) | 0.791(0.664, 0.941)^*^ |
| Marital status (married) | 1.409(1.064, 1.867)^*^ | 1.362(0.775, 2.393) | 1.320(0.357, 4.881) |
| APOEε4 | 1.823(1.572, 2.115)^*^ | 0.711(0.512, 0.986)^*^ | 1.507(0.730, 3.112) |
| FPC1 | 1.006(0.887, 1.141) | 0.907(0.692, 1.188) | 1.135(0.642, 2.007) |
| FPC2 | 1.230(1.030, 1.469)^*^ | 0.599(0.296, 1.212) | 0.304(0.037, 2.507) |
| FPC3 | 1.006(0.802, 1.262) | 1.044(0.637, 1.711) | 0.788(0.165, 3.777) |
| FPC4 | 0.864(0.588, 1.269) | 0.625(0.223, 1.749) | 0.193(0.004, 9.624) |

**Table 16 The observed and expected numbers at one-yearly intervals up to 15 years in the FMSM 2, incorporated longitudinal neuroimaging data through FPC scores in the spatial domain.**

| Years | Observed | | | Expected | | |
| --- | --- | --- | --- | --- | --- | --- |
|  | rNC | MCI | AD | rNC | MCI | AD |
| 0 | 0 | 1019 | 0 | 0.000 | 1019.000 | 0.000 |
| 1 | 32 | 832 | 115 | 35.499 | 831.580 | 111.921 |
| 2 | 42 | 561 | 234 | 52.119 | 610.017 | 174.864 |
| 3 | 45 | 366 | 278 | 55.006 | 432.043 | 201.951 |
| 4 | 39 | 238 | 317 | 53.402 | 321.266 | 219.332 |
| 5 | 36 | 156 | 329 | 50.243 | 243.705 | 227.052 |
| 6 | 38 | 122 | 338 | 49.825 | 203.968 | 244.206 |
| 7 | 35 | 98 | 341 | 47.257 | 170.124 | 256.619 |
| 8 | 28 | 74 | 347 | 44.887 | 141.127 | 262.986 |
| 9 | 16 | 54 | 353 | 41.055 | 115.943 | 266.002 |
| 10 | 11 | 39 | 356 | 38.242 | 98.750 | 269.009 |
| 11 | 7 | 20 | 359 | 34.662 | 82.748 | 268.589 |
| 12 | 3 | 8 | 359 | 32.024 | 70.353 | 267.623 |
| 13 | 2 | 4 | 359 | 30.202 | 62.255 | 272.544 |
| 14 | 2 | 1 | 359 | 28.773 | 55.766 | 277.461 |
| 15 | 2 | 1 | 359 | 27.835 | 50.689 | 283.476 |

**Table 17 Estimated transition probabilities and their 95% CIs in the FMSM 3, incorporated longitudinal neuroimaging information by including fMLFPC scores in the spatial-temporal two-dimensional continuous domain.**

| Years | MCI→rNC | MCI→MCI | MCI→AD | rNC→rNC | rNC→MCI | rNC→AD |
| --- | --- | --- | --- | --- | --- | --- |
| 1 | 0.030  (0.024, 0.038) | 0.865  (0.851, 0.877) | 0.105  (0.095, 0.117) | 0.872  (0.813, 0.913) | 0.121  (0.082, 0.176) | 0.007  (0.005, 0.011) |
| 2 | 0.052  (0.042, 0.065) | 0.752  (0.731, 0.774) | 0.196  (0.177, 0.214) | 0.764  (0.673, 0.842) | 0.209  (0.141, 0.292) | 0.026  (0.017, 0.037) |
| 3 | 0.068  (0.055, 0.084) | 0.656  (0.626, 0.683) | 0.275  (0.251, 0.303) | 0.673  (0.561, 0.768) | 0.273  (0.196, 0.367) | 0.053  (0.036, 0.076) |
| 4 | 0.080  (0.063, 0.098) | 0.576  (0.545, 0.607) | 0.345  (0.313, 0.376) | 0.595  (0.463, 0.698) | 0.318  (0.235, 0.422) | 0.087  (0.062, 0.122) |
| 5 | 0.087  (0.068, 0.108) | 0.508  (0.472, 0.542) | 0.406  (0.373, 0.439) | 0.529  (0.385, 0.658) | 0.346  (0.255, 0.448) | 0.125  (0.088, 0.171) |
| 6 | 0.091  (0.071, 0.113) | 0.449  (0.414, 0.485) | 0.460  (0.424, 0.499) | 0.472  (0.341, 0.612) | 0.363  (0.271, 0.445) | 0.165  (0.117, 0.220) |
| 7 | 0.093  (0.070, 0.117) | 0.400  (0.364, 0.439) | 0.507  (0.467, 0.546) | 0.423  (0.276, 0.555) | 0.371  (0.291, 0.449) | 0.206  (0.151, 0.281) |
| 8 | 0.093  (0.070, 0.119) | 0.357  (0.321, 0.394) | 0.550  (0.512, 0.591) | 0.380  (0.246, 0.519) | 0.372  (0.296, 0.439) | 0.248  (0.182, 0.322) |
| 9 | 0.092  (0.068, 0.119) | 0.320  (0.283, 0.356) | 0.588  (0.549, 0.629) | 0.342  (0.212, 0.481) | 0.367  (0.297, 0.423) | 0.290  (0.219, 0.374) |
| 10 | 0.090  (0.065, 0.119) | 0.288  (0.254, 0.325) | 0.622  (0.583, 0.663) | 0.310  (0.186, 0.451) | 0.359  (0.296, 0.407) | 0.331  (0.248, 0.422) |
| 11 | 0.087  (0.062, 0.117) | 0.260  (0.224, 0.298) | 0.653  (0.609, 0.692) | 0.281  (0.164, 0.421) | 0.348  (0.290, 0.390) | 0.371  (0.282, 0.459) |
| 12 | 0.084  (0.058, 0.112) | 0.235  (0.201, 0.269) | 0.681  (0.641, 0.722) | 0.256  (0.142, 0.401) | 0.335  (0.281, 0.371) | 0.410  (0.311, 0.505) |
| 13 | 0.080  (0.055, 0.108) | 0.213  (0.183, 0.248) | 0.706  (0.663, 0.745) | 0.233  (0.133, 0.370) | 0.320  (0.277, 0.353) | 0.446  (0.349, 0.539) |
| 14 | 0.076  (0.049, 0.106) | 0.194  (0.163, 0.228) | 0.729  (0.687, 0.767) | 0.213  (0.107, 0.339) | 0.305  (0.265, 0.335) | 0.482  (0.381, 0.588) |
| 15 | 0.073  (0.048, 0.104) | 0.177  (0.147, 0.208) | 0.750  (0.709, 0.788) | 0.195  (0.100, 0.32) | 0.290  (0.255, 0.316) | 0.515  (0.408, 0.613) |

**Table 18 Estimated total length of stay for post-reversion over 15 years in the FMSM 3, incorporated longitudinal neuroimaging information by including fMLFPC scores in the spatial-temporal two-dimensional continuous domain.**

| Years | Total time (years) | | | Percentage (%) | | |
| --- | --- | --- | --- | --- | --- | --- |
|  | rNC | MCI | AD | rNC | MCI | AD |
| Year 1 | 0.9343 | 0.0633 | 0.0024 | 93.43 | 6.33 | 0.24 |
| Year 2 | 1.7512 | 0.2306 | 0.0182 | 87.56 | 11.53 | 0.91 |
| Year 3 | 2.4687 | 0.4739 | 0.0573 | 82.29 | 15.80 | 1.91 |
| Year 4 | 3.102 | 0.7708 | 0.1272 | 77.55 | 19.27 | 3.18 |
| Year 5 | 3.6633 | 1.104 | 0.2327 | 73.27 | 22.08 | 4.65 |
| Year 6 | 4.163 | 1.4598 | 0.3772 | 69.38 | 24.33 | 6.29 |
| Year 7 | 4.6096 | 1.8278 | 0.5626 | 65.85 | 26.11 | 8.04 |
| Year 8 | 5.0103 | 2.1999 | 0.7899 | 62.63 | 27.50 | 9.87 |
| Year 9 | 5.371 | 2.57 | 1.059 | 59.68 | 28.56 | 11.77 |
| Year 10 | 5.6968 | 2.9336 | 1.3696 | 56.97 | 29.34 | 13.70 |
| Year 11 | 5.992 | 3.2873 | 1.7208 | 54.47 | 29.88 | 15.64 |
| Year 12 | 6.2601 | 3.6288 | 2.1111 | 52.17 | 30.24 | 17.59 |
| Year 13 | 6.5043 | 3.9565 | 2.5392 | 50.03 | 30.43 | 19.53 |
| Year 14 | 6.7272 | 4.2693 | 3.0035 | 48.05 | 30.50 | 21.45 |
| Year 15 | 6.9311 | 4.5667 | 3.5022 | 46.21 | 30.44 | 23.35 |

**Table 19 Estimated HRs and their 95% CIs in the FMSM 3, incorporated longitudinal neuroimaging information by including fMLFPC scores in the spatial-temporal two-dimensional continuous domain.**

| Covariates | HR | | |
| --- | --- | --- | --- |
|  | MCI→AD | MCI→rNC | rNC→MCI |
| Age, years | 1.030(1.015, 1.046)^*^ | 0.939(0.914, 0.964)^*^ | 0.984(0.932, 1.038) |
| Sex (female) | 1.251(0.988, 1.584) | 1.016(0.660, 1.562) | 0.660(0.290, 1.504) |
| Educational attainment, years | 1.012(0.974, 1.050) | 1.064(0.981, 1.153) | 0.797(0.673, 0.943)^*^ |
| Marital status (married) | 1.431(1.079, 1.897)^*^ | 1.404(0.799, 2.467) | 1.475(0.430, 5.058) |
| APOEε4 | 1.805(1.556, 2.094)^*^ | 0.735(0.529, 1.019) | 1.604(0.747, 3.441) |
| fMLFPC.1evel1 | 1.339(0.133, 13.471) | 1.627(0.029, 90.47) | 0.009(0.001, 2.484E+03) |
| fMLFPC.1evel2.1 | 1.207(0.198, 7.378) | 4.646(0.123, 176.013) | 0.122(0.001, 56.873) |
| fMLFPC.1evel2.2 | 25.480(0.777, 835.730) | 0.217(0.001, 2.063E+03) | 0.671(0.001, 3.469E+08) |
| fMLFPC.1evel2.3 | 1.601(0.003, 998.688) | 3.149(0.001, 2.146E+06) | 0.343(0.001, 4.836E+18) |

**Table 20 The observed and expected numbers at one-yearly intervals up to 15 years in the FMSM 3, incorporated longitudinal neuroimaging data through fMLFPC scores in the spatial-temporal two-dimensional domain.**

| Years | Observed | | | Expected | | |
| --- | --- | --- | --- | --- | --- | --- |
|  | rNC | MCI | AD | rNC | MCI | AD |
| 0 | 0 | 1019 | 0 | 0.000 | 1019.000 | 0.000 |
| 1 | 32 | 832 | 115 | 35.599 | 831.380 | 112.021 |
| 2 | 42 | 561 | 234 | 51.962 | 610.044 | 174.994 |
| 3 | 45 | 366 | 278 | 54.622 | 432.237 | 202.141 |
| 4 | 39 | 238 | 317 | 52.798 | 321.587 | 219.615 |
| 5 | 36 | 156 | 329 | 49.524 | 244.160 | 227.316 |
| 6 | 38 | 122 | 338 | 49.000 | 204.516 | 244.483 |
| 7 | 35 | 98 | 341 | 46.521 | 170.513 | 256.966 |
| 8 | 28 | 74 | 347 | 43.953 | 141.625 | 263.422 |
| 9 | 16 | 54 | 353 | 39.964 | 116.501 | 266.535 |
| 10 | 11 | 39 | 356 | 37.142 | 99.231 | 269.627 |
| 11 | 7 | 20 | 359 | 33.443 | 83.250 | 269.307 |
| 12 | 3 | 8 | 359 | 30.674 | 70.890 | 268.436 |
| 13 | 2 | 4 | 359 | 28.852 | 62.720 | 273.428 |
| 14 | 2 | 1 | 359 | 27.471 | 56.146 | 278.383 |
| 15 | 2 | 1 | 359 | 26.500 | 51.018 | 284.482 |

**Table 21 Estimated transition probabilities and their 95% CIs in the FMSM 4, combining longitudinal markers from FMSMs 1 and 2.**

| Years | MCI→rNC | MCI→MCI | MCI→AD | rNC→rNC | rNC→MCI | rNC→AD |
| --- | --- | --- | --- | --- | --- | --- |
| 1 | 0.019  (0.014, 0.026) | 0.899  (0.885, 0.910) | 0.082  (0.072, 0.094) | 0.885  (0.788, 0.943) | 0.110  (0.054, 0.203) | 0.005  (0.002, 0.009) |
| 2 | 0.034  (0.024, 0.046) | 0.810  (0.788, 0.830) | 0.156  (0.137, 0.178) | 0.786  (0.609, 0.890) | 0.196  (0.101, 0.354) | 0.018  (0.009, 0.035) |
| 3 | 0.045  (0.032, 0.062) | 0.732  (0.702, 0.760) | 0.223  (0.199, 0.251) | 0.700  (0.491, 0.851) | 0.262  (0.131, 0.440) | 0.038  (0.018, 0.069) |
| 4 | 0.054  (0.037, 0.076) | 0.663  (0.626, 0.697) | 0.283  (0.251, 0.319) | 0.625  (0.354, 0.809) | 0.312  (0.161, 0.528) | 0.063  (0.031, 0.119) |
| 5 | 0.060  (0.040, 0.084) | 0.602  (0.562, 0.642) | 0.338  (0.299, 0.380) | 0.559  (0.296, 0.762) | 0.349  (0.191, 0.540) | 0.092  (0.047, 0.160) |
| 6 | 0.064  (0.041, 0.089) | 0.548  (0.503, 0.589) | 0.388  (0.346, 0.434) | 0.502  (0.246, 0.717) | 0.375  (0.215, 0.550) | 0.123  (0.065, 0.207) |
| 7 | 0.067  (0.043, 0.096) | 0.499  (0.453, 0.542) | 0.433  (0.393, 0.480) | 0.451  (0.191, 0.673) | 0.392  (0.239, 0.550) | 0.157  (0.087, 0.264) |
| 8 | 0.069  (0.042, 0.102) | 0.456  (0.407, 0.505) | 0.475  (0.425, 0.528) | 0.407  (0.162, 0.643) | 0.402  (0.250, 0.541) | 0.191  (0.108, 0.300) |
| 9 | 0.070  (0.042, 0.101) | 0.418  (0.365, 0.468) | 0.512  (0.462, 0.565) | 0.368  (0.148, 0.617) | 0.406  (0.254, 0.518) | 0.226  (0.126, 0.346) |
| 10 | 0.070  (0.039, 0.107) | 0.383  (0.336, 0.437) | 0.547  (0.496, 0.600) | 0.333  (0.118, 0.587) | 0.405  (0.263, 0.509) | 0.261  (0.149, 0.394) |
| 11 | 0.069  (0.036, 0.108) | 0.352  (0.304, 0.403) | 0.579  (0.528, 0.631) | 0.303  (0.095, 0.568) | 0.401  (0.259, 0.483) | 0.296  (0.169, 0.436) |
| 12 | 0.068  (0.035, 0.109) | 0.324  (0.275, 0.372) | 0.608  (0.555, 0.661) | 0.276  (0.086, 0.543) | 0.393  (0.260, 0.458) | 0.331  (0.194, 0.478) |
| 13 | 0.066  (0.035, 0.105) | 0.299  (0.249, 0.349) | 0.635  (0.585, 0.686) | 0.252  (0.081, 0.515) | 0.384  (0.262, 0.442) | 0.365  (0.212, 0.517) |
| 14 | 0.064  (0.030, 0.104) | 0.276  (0.230, 0.325) | 0.660  (0.604, 0.716) | 0.230  (0.063, 0.472) | 0.373  (0.277, 0.422) | 0.397  (0.248, 0.552) |
| 15 | 0.062  (0.030, 0.105) | 0.255  (0.206, 0.304) | 0.683  (0.623, 0.738) | 0.211  (0.058, 0.475) | 0.360  (0.263, 0.404) | 0.429  (0.26, 0.587) |

**Table 22 Estimated total length of stay for post-reversion over 15 years in the FMSM 4, combining longitudinal markers from FMSMs 1 and 2.**

| Years | Total time (years) | | | Percentage (%) | | |
| --- | --- | --- | --- | --- | --- | --- |
|  | rNC | MCI | AD | rNC | MCI | AD |
| Year 1 | 0.9414 | 0.0569 | 0.0017 | 94.14 | 5.69 | 0.17 |
| Year 2 | 1.7761 | 0.2113 | 0.0127 | 88.81 | 10.57 | 0.64 |
| Year 3 | 2.5181 | 0.4415 | 0.0405 | 83.94 | 14.72 | 1.35 |
| Year 4 | 3.1794 | 0.7297 | 0.0909 | 79.49 | 18.24 | 2.27 |
| Year 5 | 3.7705 | 1.0613 | 0.1682 | 75.41 | 21.23 | 3.36 |
| Year 6 | 4.3001 | 1.4241 | 0.2758 | 71.67 | 23.74 | 4.60 |
| Year 7 | 4.7759 | 1.8084 | 0.4157 | 68.23 | 25.83 | 5.94 |
| Year 8 | 5.2045 | 2.2059 | 0.5896 | 65.06 | 27.57 | 7.37 |
| Year 9 | 5.5914 | 2.6102 | 0.7983 | 62.13 | 29.00 | 8.87 |
| Year 10 | 5.9417 | 3.0161 | 1.0422 | 59.42 | 30.16 | 10.42 |
| Year 11 | 6.2595 | 3.4194 | 1.3211 | 56.90 | 31.09 | 12.01 |
| Year 12 | 6.5485 | 3.8168 | 1.6348 | 54.57 | 31.81 | 13.62 |
| Year 13 | 6.8119 | 4.2056 | 1.9825 | 52.40 | 32.35 | 15.25 |
| Year 14 | 7.0524 | 4.5841 | 2.3635 | 50.37 | 32.74 | 16.88 |
| Year 15 | 7.2725 | 4.9507 | 2.7768 | 48.48 | 33.00 | 18.51 |

**Table 23 Estimated HRs and their 95% CIs in the FMSM 4, combining longitudinal markers from FMSMs 1 and 2.**

| Covariates | HR | | |
| --- | --- | --- | --- |
|  | MCI→AD | MCI→rNC | rNC→MCI |
| Age, years | 1.007(0.991, 1.022) | 0.986(0.957, 1.017) | 1.000(0.940, 1.064) |
| Sex (female) | 1.194(0.946, 1.506) | 1.047(0.670, 1.636) | 1.035(0.448, 2.387) |
| Educational attainment, years | 1.052(1.014, 1.091)^*^ | 0.990(0.906, 1.081) | 0.814(0.671, 0.987)^*^ |
| Marital status (married) | 1.272(0.964, 1.678) | 1.001(0.581, 1.723) | 0.768(0.272, 2.164) |
| APOEε4 | 1.374(1.176, 1.606)^*^ | 1.135(0.801, 1.608) | 1.593(0.672, 3.778) |
| MVFPC1 | 1.003(1.002, 1.004)^*^ | 0.993(0.991, 0.995)^*^ | 1.000(0.995, 1.004) |
| MVFPC2 | 0.992(0.991, 0.994)^*^ | 1.004(1.001, 1.007)^*^ | 1.004(0.999, 1.009) |
| FPC1 | 1.006(0.888, 1.140) | 1.035(0.806, 1.329) | 1.245(0.656, 2.362) |
| FPC2 | 1.218(1.015, 1.461)^*^ | 0.952(0.550, 1.647) | 1.077(0.388, 2.988) |
| FPC3 | 1.014(0.801, 1.284) | 0.989(0.586, 1.671) | 0.623(0.125, 3.104) |
| FPC4 | 1.105(0.698, 1.751) | 0.915(0.267, 3.133) | 1.169(0.041, 33.118) |

**Table 24 The observed and expected numbers at one-yearly intervals up to 15 years in the FMSM 4, incorporated MVFPC scores derived from eleven longitudinal neuropsychological scales and longitudinal neuroimaging data through FPC scores in the spatial domain.**

| Years | Observed | | | Expected | | |
| --- | --- | --- | --- | --- | --- | --- |
|  | rNC | MCI | AD | rNC | MCI | AD |
| 0 | 0 | 1019 | 0 | 0.000 | 1019.000 | 0.000 |
| 1 | 32 | 832 | 115 | 30.277 | 830.980 | 117.743 |
| 2 | 42 | 561 | 234 | 45.517 | 606.162 | 185.321 |
| 3 | 45 | 366 | 278 | 47.536 | 424.104 | 217.360 |
| 4 | 39 | 238 | 317 | 45.519 | 310.381 | 238.100 |
| 5 | 36 | 156 | 329 | 40.520 | 229.787 | 250.693 |
| 6 | 38 | 122 | 338 | 39.414 | 190.162 | 268.424 |
| 7 | 35 | 98 | 341 | 36.747 | 155.989 | 281.264 |
| 8 | 28 | 74 | 347 | 33.824 | 127.646 | 287.530 |
| 9 | 16 | 54 | 353 | 29.026 | 102.941 | 291.033 |
| 10 | 11 | 39 | 356 | 25.592 | 86.217 | 294.190 |
| 11 | 7 | 20 | 359 | 19.921 | 68.615 | 297.464 |
| 12 | 3 | 8 | 359 | 15.663 | 55.075 | 299.262 |
| 13 | 2 | 4 | 359 | 14.566 | 47.818 | 302.616 |
| 14 | 2 | 1 | 359 | 13.484 | 41.865 | 306.651 |
| 15 | 2 | 1 | 359 | 13.104 | 37.846 | 311.050 |

**Table 25 The observed and expected numbers at one-yearly intervals up to 15 years in the FMSM 5, incorporated MVFPC scores derived from eleven longitudinal neuropsychological scales and longitudinal neuroimaging data through fMLFPC scores in the spatial-temporal two-dimensional domain.**

| Years | Observed | | | Expected | | |
| --- | --- | --- | --- | --- | --- | --- |
|  | rNC | MCI | AD | rNC | MCI | AD |
| 0 | 0 | 1019 | 0 | 0 | 1019 | 0 |
| 1 | 32 | 832 | 115 | 30.281 | 832.820 | 115.900 |
| 2 | 42 | 561 | 234 | 45.423 | 609.289 | 182.288 |
| 3 | 45 | 366 | 278 | 47.391 | 427.681 | 213.928 |
| 4 | 39 | 238 | 317 | 45.161 | 313.953 | 234.886 |
| 5 | 36 | 156 | 329 | 40.494 | 233.217 | 247.289 |
| 6 | 38 | 122 | 338 | 39.722 | 193.553 | 264.725 |
| 7 | 35 | 98 | 341 | 37.233 | 159.234 | 277.533 |
| 8 | 28 | 74 | 347 | 34.641 | 130.489 | 283.870 |
| 9 | 16 | 54 | 353 | 30.037 | 105.374 | 287.588 |
| 10 | 11 | 39 | 356 | 26.778 | 88.420 | 290.803 |
| 11 | 7 | 20 | 359 | 21.219 | 70.682 | 294.100 |
| 12 | 3 | 8 | 359 | 17.267 | 56.933 | 295.800 |
| 13 | 2 | 4 | 359 | 16.275 | 49.514 | 299.211 |
| 14 | 2 | 1 | 359 | 15.496 | 43.308 | 303.196 |
| 15 | 2 | 1 | 359 | 15.228 | 39.214 | 307.558 |

**Table 26 Estimated transition probabilities and their 95% CIs in the MSM 1, incorporated longitudinal cognitive information by including the PC scores derived from the eleven cognitive assessments.**

| Years | MCI→rNC | MCI→MCI | MCI→AD | rNC→rNC | rNC→MCI | rNC→AD |
| --- | --- | --- | --- | --- | --- | --- |
| 1 | 0.012  (0.007，0.018) | 0.925  (0.913，0.935) | 0.063  (0.054，0.074) | 0.870  (0.505，0.972) | 0.126  (0.027，0.480) | 0.004  (0.001，0.018) |
| 2 | 0.021  (0.012，0.032) | 0.857  (0.835，0.877) | 0.122  (0.106，0.141) | 0.759  (0.246，0.942) | 0.225  (0.054，0.694) | 0.016  (0.004，0.058) |
| 3 | 0.028  (0.016，0.044) | 0.795  (0.767，0.823) | 0.176  (0.152，0.203) | 0.663  (0.167，0.918) | 0.304  (0.074，0.731) | 0.034  (0.008，0.102) |
| 4 | 0.034  (0.015，0.052) | 0.739  (0.704，0.775) | 0.227  (0.197，0.262) | 0.580  (0.066，0.897) | 0.364  (0.090，0.770) | 0.056  (0.013，0.160) |
| 5 | 0.038  (0.015，0.058) | 0.688  (0.647，0.727) | 0.274  (0.239，0.315) | 0.509  (0.036，0.883) | 0.410  (0.099，0.744) | 0.081  (0.018，0.214) |
| 6 | 0.041  (0.014，0.066) | 0.641  (0.599，0.685) | 0.318  (0.279，0.358) | 0.448  (0.027，0.872) | 0.443  (0.106，0.717) | 0.109  (0.022，0.261) |
| 7 | 0.043  (0.014，0.072) | 0.598  (0.551，0.649) | 0.358  (0.314，0.405) | 0.395  (0.020，0.829) | 0.466  (0.135，0.687) | 0.139  (0.036，0.308) |
| 8 | 0.045  (0.012，0.079) | 0.559  (0.507，0.612) | 0.396  (0.348，0.445) | 0.349  (0.019，0.783) | 0.480  (0.167，0.657) | 0.170  (0.050，0.346) |
| 9 | 0.045  (0.014，0.080) | 0.522  (0.470，0.579) | 0.432  (0.379，0.486) | 0.309  (0.019，0.774) | 0.488  (0.168，0.631) | 0.202  (0.055，0.389) |
| 10 | 0.046  (0.012，0.080) | 0.489  (0.431，0.541) | 0.465  (0.415，0.524) | 0.275  (0.014，0.769) | 0.490  (0.166，0.605) | 0.235  (0.065，0.427) |
| 11 | 0.045  (0.011，0.086) | 0.458  (0.400，0.512) | 0.497  (0.445，0.554) | 0.245  (0.014，0.755) | 0.488  (0.170，0.579) | 0.267  (0.074，0.466) |
| 12 | 0.045  (0.011，0.091) | 0.429  (0.371，0.486) | 0.526  (0.470，0.585) | 0.219  (0.013，0.717) | 0.482  (0.191，0.556) | 0.299  (0.093，0.496) |
| 13 | 0.044  (0.010，0.087) | 0.403  (0.345，0.458) | 0.553  (0.501，0.614) | 0.196  (0.011，0.722) | 0.474  (0.183，0.535) | 0.330  (0.098，0.536) |
| 14 | 0.043  (0.010，0.093) | 0.378  (0.316，0.439) | 0.579  (0.520，0.637) | 0.176  (0.012，0.715) | 0.463  (0.180，0.515) | 0.361  (0.105，0.561) |
| 15 | 0.042  (0.008，0.092) | 0.355  (0.297，0.411) | 0.603  (0.542，0.666) | 0.159  (0.009，0.679) | 0.450  (0.195，0.491) | 0.391  (0.122，0.594) |

**Table 27 Estimated total length of stay for post-reversion over 15 years in the MSM 1, incorporated longitudinal cognitive information by including the PC scores derived from the eleven cognitive assessments.**

| Years | Total time (years) | | | Percentage (%) | | |
| --- | --- | --- | --- | --- | --- | --- |
|  | rNC | MCI | AD | rNC | MCI | AD |
| Year 1 | 0.9334 | 0.0651 | 0.0015 | 93.34 | 6.51 | 0.15 |
| Year 2 | 1.7465 | 0.2425 | 0.011 | 87.33 | 12.13 | 0.55 |
| Year 3 | 2.456 | 0.5086 | 0.0353 | 81.87 | 16.95 | 1.18 |
| Year 4 | 3.0766 | 0.8439 | 0.0796 | 76.92 | 21.10 | 1.99 |
| Year 5 | 3.6205 | 1.2318 | 0.1477 | 72.41 | 24.64 | 2.95 |
| Year 6 | 4.0983 | 1.659 | 0.2427 | 68.31 | 27.65 | 4.05 |
| Year 7 | 4.5191 | 2.1141 | 0.3668 | 64.56 | 30.20 | 5.24 |
| Year 8 | 4.8906 | 2.5878 | 0.5216 | 61.13 | 32.35 | 6.52 |
| Year 9 | 5.2194 | 3.0726 | 0.7079 | 57.99 | 34.14 | 7.87 |
| Year 10 | 5.5112 | 3.5623 | 0.9264 | 55.11 | 35.62 | 9.26 |
| Year 11 | 5.7709 | 4.0519 | 1.1772 | 52.46 | 36.84 | 10.70 |
| Year 12 | 6.0025 | 4.5374 | 1.4601 | 50.02 | 37.81 | 12.17 |
| Year 13 | 6.2098 | 5.0155 | 1.7747 | 47.77 | 38.58 | 13.65 |
| Year 14 | 6.3957 | 5.4837 | 2.1206 | 45.68 | 39.17 | 15.15 |
| Year 15 | 6.563 | 5.9402 | 2.4968 | 43.75 | 39.60 | 16.65 |

**Table 28 Estimated HRs and their 95% CIs in the MSM 1, incorporated longitudinal cognitive information by including the PC scores derived from the eleven cognitive assessments.**

| Covariates | HR | | |
| --- | --- | --- | --- |
|  | MCI→AD | MCI→rNC | rNC→MCI |
| Age, years | 0.999(0.985，1.014) | 0.972(0.943，1.002) | 0.981(0.928，1.036) |
| Sex (female) | 1.079(0.850，1.371) | 0.834(0.531，1.307) | 0.681(0.262，1.767) |
| Educational attainment, years | 1.032(0.995，1.070) | 0.972(0.889，1.062) | 0.804(0.661，0.977)^*^ |
| Marital status (married) | 1.047(0.783，1.400) | 1.772(1.004，3.127)^*^ | 1.752(0.482，6.361) |
| APOEε4 | 1.418(1.212，1.660)^*^ | 1.024(0.693，1.513) | 1.831(0.638，5.252) |
| PC1 | 1.605(1.527，1.688)^*^ | 0.436(0.345，0.551)^*^ | 0.936(0.467，1.875) |
| PC2 | 0.873(0.807，0.945)^*^ | 1.569(1.274，1.932)^*^ | 1.265(0.745，2.148) |
| PC3 | 0.857(0.764，0.960)^*^ | 2.215(1.466，3.346)^*^ | 0.945(0.398，2.245) |
| PC4 | 0.746(0.677，0.822)^*^ | 2.909(1.453，5.826)^*^ | 1.113(0.183，6.789) |
| PC5 | 0.846(0.746，0.959)^*^ | 1.570(1.079，2.284)^*^ | 1.005(0.482，2.095) |

**Table 29 The observed and expected numbers at one-yearly intervals up to 15 years in the MSM 1, incorporated longitudinal cognitive information by including the PC scores derived from the eleven cognitive assessments.**

| Years | Observed | | | Expected | | |
| --- | --- | --- | --- | --- | --- | --- |
|  | rNC | MCI | AD | rNC | MCI | AD |
| 0 | 0 | 1019 | 0 | 0.000 | 1019.000 | 0.000 |
| 1 | 32 | 832 | 115 | 29.861 | 840.504 | 108.635 |
| 2 | 42 | 561 | 234 | 46.611 | 610.838 | 179.551 |
| 3 | 45 | 366 | 278 | 47.735 | 425.806 | 215.459 |
| 4 | 39 | 238 | 317 | 43.981 | 310.712 | 239.307 |
| 5 | 36 | 156 | 329 | 38.255 | 228.259 | 254.486 |
| 6 | 38 | 122 | 338 | 36.067 | 188.807 | 273.127 |
| 7 | 35 | 98 | 341 | 32.148 | 155.186 | 286.666 |
| 8 | 28 | 74 | 347 | 28.208 | 126.091 | 294.701 |
| 9 | 16 | 54 | 353 | 21.962 | 100.440 | 300.598 |
| 10 | 11 | 39 | 356 | 17.754 | 83.043 | 305.203 |
| 11 | 7 | 20 | 359 | 10.794 | 65.781 | 309.425 |
| 12 | 3 | 8 | 359 | 6.247 | 51.986 | 311.767 |
| 13 | 2 | 4 | 359 | 4.838 | 45.206 | 314.956 |
| 14 | 2 | 1 | 359 | 4.285 | 39.563 | 318.153 |
| 15 | 2 | 1 | 359 | 4.196 | 35.887 | 321.916 |

**Table 30 Estimated transition probabilities and their 95% CIs in the MSM 2, incorporated longitudinal neuroimaging information by including the PC scores.**

| Years | MCI→rNC | MCI→MCI | MCI→AD | rNC→rNC | rNC→MCI | rNC→AD |
| --- | --- | --- | --- | --- | --- | --- |
| 1 | 0.028  (0.022，0.035) | 0.866  (0.854，0.878) | 0.105  (0.095，0.117) | 0.890  (0.827，0.930) | 0.104  (0.066，0.163) | 0.006  (0.004，0.01) |
| 2 | 0.050  (0.040，0.062) | 0.753  (0.731，0.774) | 0.197  (0.177，0.219) | 0.794  (0.696，0.866) | 0.183  (0.119，0.268) | 0.023  (0.014，0.034) |
| 3 | 0.065  (0.052，0.082) | 0.658  (0.630，0.685) | 0.277  (0.252，0.303) | 0.712  (0.584，0.809) | 0.241  (0.16，0.346) | 0.047  (0.030，0.072) |
| 4 | 0.077  (0.060，0.098) | 0.577  (0.544，0.607) | 0.347  (0.317，0.379) | 0.640  (0.489，0.760) | 0.283  (0.190，0.399) | 0.077  (0.049，0.115) |
| 5 | 0.085  (0.066，0.107) | 0.508  (0.473，0.543) | 0.408  (0.375，0.442) | 0.577  (0.421，0.716) | 0.312  (0.213，0.421) | 0.111  (0.071，0.158) |
| 6 | 0.090  (0.069，0.117) | 0.449  (0.411，0.486) | 0.462  (0.425，0.500) | 0.522  (0.361，0.658) | 0.331  (0.237，0.432) | 0.147  (0.100，0.211) |
| 7 | 0.092  (0.070，0.119) | 0.398  (0.360，0.435) | 0.510  (0.472，0.549) | 0.474  (0.308，0.622) | 0.341  (0.247，0.434) | 0.185  (0.126，0.260) |
| 8 | 0.093  (0.068，0.122) | 0.354  (0.317，0.392) | 0.552  (0.515，0.593) | 0.431  (0.262，0.576) | 0.345  (0.262，0.430) | 0.224  (0.156，0.309) |
| 9 | 0.093  (0.065，0.123) | 0.317  (0.280，0.355) | 0.590  (0.552，0.631) | 0.393  (0.232，0.557) | 0.344  (0.257，0.417) | 0.263  (0.183，0.360) |
| 10 | 0.092  (0.062，0.124) | 0.284  (0.245，0.320) | 0.624  (0.581，0.666) | 0.360  (0.201，0.528) | 0.339  (0.258，0.403) | 0.302  (0.209，0.410) |
| 11 | 0.090  (0.062，0.122) | 0.256  (0.217，0.293) | 0.655  (0.613，0.700) | 0.330  (0.187，0.501) | 0.331  (0.257，0.384) | 0.340  (0.240，0.448) |
| 12 | 0.087  (0.058，0.122) | 0.231  (0.193，0.264) | 0.682  (0.643，0.723) | 0.303  (0.151，0.474) | 0.321  (0.257，0.366) | 0.376  (0.267，0.491) |
| 13 | 0.084  (0.055，0.118) | 0.209  (0.176，0.247) | 0.707  (0.663，0.745) | 0.278  (0.140，0.446) | 0.310  (0.253，0.350) | 0.412  (0.298，0.526) |
| 14 | 0.081  (0.050，0.115) | 0.190  (0.155，0.225) | 0.730  (0.688，0.770) | 0.256  (0.123，0.416) | 0.297  (0.248，0.332) | 0.447  (0.330，0.572) |
| 15 | 0.077  (0.045，0.111) | 0.173  (0.144，0.204) | 0.750  (0.707，0.789) | 0.236  (0.107，0.409) | 0.284  (0.238，0.313) | 0.479  (0.353，0.602) |

**Table 31 Estimated total length of stay for post-reversion over 15 years in the MSM 2, incorporated longitudinal neuroimaging information by including the PC scores.**

| Years | Total time (years) | | | Percentage (%) | | |
| --- | --- | --- | --- | --- | --- | --- |
|  | rNC | MCI | AD | rNC | MCI | AD |
| Year 1 | 0.9434 | 0.0545 | 0.0021 | 94.34 | 5.45 | 0.21 |
| Year 2 | 1.7842 | 0.2000 | 0.0158 | 89.21 | 10.00 | 0.79 |
| Year 3 | 2.5362 | 0.4138 | 0.0500 | 84.54 | 13.79 | 1.67 |
| Year 4 | 3.2113 | 0.6773 | 0.1114 | 80.28 | 16.93 | 2.79 |
| Year 5 | 3.8193 | 0.9760 | 0.2047 | 76.39 | 19.52 | 4.09 |
| Year 6 | 4.3685 | 1.2982 | 0.3333 | 72.81 | 21.64 | 5.56 |
| Year 7 | 4.8662 | 1.6345 | 0.4993 | 69.52 | 23.35 | 7.13 |
| Year 8 | 5.3185 | 1.9777 | 0.7037 | 66.48 | 24.72 | 8.80 |
| Year 9 | 5.7305 | 2.3222 | 0.9472 | 63.67 | 25.80 | 10.52 |
| Year 10 | 6.1068 | 2.6636 | 1.2296 | 61.07 | 26.64 | 12.30 |
| Year 11 | 6.4512 | 2.9986 | 1.5502 | 58.65 | 27.26 | 14.09 |
| Year 12 | 6.7670 | 3.3246 | 1.9084 | 56.39 | 27.71 | 15.90 |
| Year 13 | 7.0572 | 3.6400 | 2.3028 | 54.29 | 28.00 | 17.71 |
| Year 14 | 7.3242 | 3.9435 | 2.7323 | 52.32 | 28.17 | 19.52 |
| Year 15 | 7.5703 | 4.2342 | 3.1955 | 50.47 | 28.23 | 21.30 |

**Table 32 Estimated HRs and their 95% CIs in the MSM 2, incorporated longitudinal neuroimaging information by including the PC scores.**

| Covariates | HR | | |
| --- | --- | --- | --- |
|  | MCI→AD | MCI→rNC | rNC→MCI |
| Age, years | 1.029(1.014，1.045)^*^ | 0.942(0.917，0.967)^*^ | 0.983(0.930，1.040) |
| Sex (female) | 1.315(1.039，1.664)^*^ | 0.906(0.584，1.406) | 0.613(0.269，1.396) |
| Educational attainment, years | 1.014(0.976，1.052) | 1.069(0.986，1.159) | 0.823(0.692，0.979)^*^ |
| Marital status (married) | 1.321(1.004，1.737)^*^ | 1.301(0.739，2.292) | 1.074(0.310，3.722) |
| APOEε4 | 1.846(1.593，2.140)^*^ | 0.704(0.507，0.977)^*^ | 1.250(0.576，2.714) |
| Cog_PC1 | 0.997(0.984，1.010) | 1.023(0.989，1.058) | 1.015(0.963，1.068) |
| Cog_PC2 | 0.985(0.965，1.005) | 1.083(1.000，1.174)^*^ | 1.131(0.955，1.339) |
| Cog_PC3 | 1.007(0.977，1.038) | 0.996(0.914，1.085) | 1.058(0.833，1.343) |
| Cog_PC4 | 1.021(0.976，1.068) | 0.952(0.821，1.105) | 0.939(0.712，1.238) |
| Cog_PC5 | 1.084(1.034，1.137)^*^ | 0.880(0.722，1.071) | 0.908(0.621，1.327) |

**Table 33 The observed and expected numbers at one-yearly intervals up to 15 years in the MSM 2, incorporated longitudinal neuroimaging information by including the PC scores.**

| Years | Observed | | | Expected | | |
| --- | --- | --- | --- | --- | --- | --- |
|  | rNC | MCI | AD | rNC | MCI | AD |
| 0 | 0 | 1019 | 0 | 0.000 | 1019.000 | 0.000 |
| 1 | 32 | 832 | 115 | 34.974 | 830.246 | 113.780 |
| 2 | 42 | 561 | 234 | 51.290 | 607.822 | 177.889 |
| 3 | 45 | 366 | 278 | 54.171 | 430.115 | 204.715 |
| 4 | 39 | 238 | 317 | 52.404 | 319.120 | 222.476 |
| 5 | 36 | 156 | 329 | 49.328 | 241.833 | 229.839 |
| 6 | 38 | 122 | 338 | 48.613 | 202.081 | 247.306 |
| 7 | 35 | 98 | 341 | 45.993 | 168.273 | 259.734 |
| 8 | 28 | 74 | 347 | 43.594 | 139.358 | 266.049 |
| 9 | 16 | 54 | 353 | 39.686 | 114.185 | 269.129 |
| 10 | 11 | 39 | 356 | 36.855 | 97.140 | 272.005 |
| 11 | 7 | 20 | 359 | 33.217 | 81.175 | 271.608 |
| 12 | 3 | 8 | 359 | 30.499 | 68.988 | 270.513 |
| 13 | 2 | 4 | 359 | 28.677 | 61.054 | 275.269 |
| 14 | 2 | 1 | 359 | 27.233 | 54.600 | 280.167 |
| 15 | 2 | 1 | 359 | 26.255 | 49.629 | 286.116 |

**Table 34 Estimated transition probabilities and their 95% CIs in the MSM 3, combining longitudinal markers from MSMs 1 and 2.**

| Years | MCI→rNC | MCI→MCI | MCI→AD | rNC→rNC | rNC→MCI | rNC→AD |
| --- | --- | --- | --- | --- | --- | --- |
| 1 | 0.011  (0.007，0.017) | 0.926  (0.915，0.936) | 0.063  (0.054，0.073) | 0.899  (0.575，0.981) | 0.098  (0.019，0.411) | 0.003  (0.001，0.015) |
| 2 | 0.020  (0.012，0.030) | 0.859  (0.838，0.878) | 0.121  (0.104，0.140) | 0.809  (0.338，0.957) | 0.179  (0.040，0.614) | 0.012  (0.003，0.051) |
| 3 | 0.028  (0.015，0.044) | 0.798  (0.768，0.823) | 0.175  (0.151，0.202) | 0.729  (0.19，0.938) | 0.245  (0.057，0.716) | 0.026  (0.006，0.097) |
| 4 | 0.034  (0.015，0.053) | 0.742  (0.708，0.773) | 0.225  (0.196，0.259) | 0.658  (0.091，0.918) | 0.298  (0.072，0.753) | 0.044  (0.010，0.151) |
| 5 | 0.039  (0.017，0.059) | 0.690  (0.651，0.731) | 0.271  (0.236，0.307) | 0.595  (0.08，0.897) | 0.341  (0.088，0.738) | 0.065  (0.015，0.190) |
| 6 | 0.042  (0.018，0.068) | 0.643  (0.603，0.689) | 0.315  (0.277，0.352) | 0.538  (0.072，0.893) | 0.374  (0.088，0.706) | 0.088  (0.019，0.226) |
| 7 | 0.045  (0.015，0.074) | 0.600  (0.551，0.649) | 0.355  (0.311，0.402) | 0.488  (0.028，0.867) | 0.399  (0.104，0.688) | 0.113  (0.028，0.291) |
| 8 | 0.047  (0.016，0.078) | 0.560  (0.509，0.614) | 0.393  (0.345，0.440) | 0.443  (0.031，0.846) | 0.417  (0.119，0.658) | 0.140  (0.035，0.323) |
| 9 | 0.049  (0.013，0.082) | 0.523  (0.473，0.577) | 0.428  (0.381，0.482) | 0.403  (0.021，0.844) | 0.430  (0.119，0.632) | 0.168  (0.039，0.378) |
| 10 | 0.050  (0.014，0.086) | 0.489  (0.434，0.548) | 0.461  (0.410，0.518) | 0.367  (0.02，0.814) | 0.438  (0.134，0.609) | 0.196  (0.052，0.413) |
| 11 | 0.050  (0.013，0.089) | 0.458  (0.399，0.513) | 0.492  (0.441，0.552) | 0.334  (0.018，0.819) | 0.441  (0.127，0.580) | 0.224  (0.053，0.455) |
| 12 | 0.050  (0.012，0.095) | 0.429  (0.368，0.488) | 0.521  (0.468，0.576) | 0.305  (0.016，0.791) | 0.441  (0.140，0.557) | 0.253  (0.066，0.482) |
| 13 | 0.050  (0.010，0.097) | 0.403  (0.345，0.461) | 0.548  (0.492，0.608) | 0.279  (0.012，0.784) | 0.439  (0.140，0.535) | 0.282  (0.075，0.521) |
| 14 | 0.049  (0.009，0.093) | 0.378  (0.316，0.434) | 0.573  (0.512，0.634) | 0.256  (0.011，0.766) | 0.434  (0.151，0.514) | 0.310  (0.084，0.554) |
| 15 | 0.048  (0.011，0.101) | 0.355  (0.297，0.415) | 0.597  (0.539，0.660) | 0.235  (0.013，0.787) | 0.427  (0.134，0.491) | 0.338  (0.080，0.577) |

**Table 35 Estimated total length of stay for post-reversion over 15 years in the MSM 3, combining longitudinal markers from MSMs 1 and 2.**

| Years | Total time (years) | | | Percentage (%) | | |
| --- | --- | --- | --- | --- | --- | --- |
|  | rNC | MCI | AD | rNC | MCI | AD |
| Year 1 | 0.9484 | 0.0505 | 0.0011 | 94.84 | 5.05 | 0.11 |
| Year 2 | 1.8013 | 0.1902 | 0.0085 | 90.07 | 9.51 | 0.43 |
| Year 3 | 2.5693 | 0.4032 | 0.0275 | 85.64 | 13.44 | 0.92 |
| Year 4 | 3.262 | 0.6757 | 0.0623 | 81.55 | 16.89 | 1.56 |
| Year 5 | 3.8875 | 0.996 | 0.1165 | 77.75 | 19.92 | 2.33 |
| Year 6 | 4.4533 | 1.3539 | 0.1928 | 74.22 | 22.57 | 3.21 |
| Year 7 | 4.9657 | 1.7408 | 0.2934 | 70.94 | 24.87 | 4.19 |
| Year 8 | 5.4306 | 2.1495 | 0.42 | 67.88 | 26.87 | 5.25 |
| Year 9 | 5.8529 | 2.5735 | 0.5737 | 65.03 | 28.59 | 6.37 |
| Year 10 | 6.2371 | 3.0076 | 0.7553 | 62.37 | 30.08 | 7.55 |
| Year 11 | 6.5872 | 3.4473 | 0.9654 | 59.88 | 31.34 | 8.78 |
| Year 12 | 6.9068 | 3.8889 | 1.2043 | 57.56 | 32.41 | 10.04 |
| Year 13 | 7.1989 | 4.3293 | 1.4718 | 55.38 | 33.30 | 11.32 |
| Year 14 | 7.4663 | 4.7658 | 1.7679 | 53.33 | 34.04 | 12.63 |
| Year 15 | 7.7115 | 5.1962 | 2.0923 | 51.41 | 34.64 | 13.95 |

**Table 36 Estimated HRs and their 95% CIs in the MSM 3, combining longitudinal markers from MSMs 1 and 2.**

| Covariates | HR | | |
| --- | --- | --- | --- |
|  | MCI→AD | MCI→rNC | rNC→MCI |
| Age, years | 0.999(0.985，1.014) | 0.972(0.943，1.002) | 0.981(0.928，1.036) |
| Sex (female) | 1.079(0.850，1.371) | 0.834(0.531，1.307) | 0.681(0.262，1.767) |
| Educational attainment, years | 1.032(0.995，1.070) | 0.972(0.889，1.062) | 0.804(0.661，0.977)^*^ |
| Marital status (married) | 0.998(0.983，1.013) | 0.975(0.945，1.005) | 0.982(0.925，1.044) |
| APOEε4 | 1.131(0.887，1.444) | 0.759(0.480，1.200) | 0.576(0.207，1.603) |
| Cog_PC1 | 1.029(0.992，1.067) | 0.981(0.897，1.072) | 0.807(0.660，0.987)^*^ |
| Cog_PC2 | 1.033(0.772，1.381) | 1.737(0.983，3.070) | 1.430(0.376，5.433) |
| Cog_PC3 | 1.420(1.213，1.664)^*^ | 0.981(0.691，1.393) | 1.402(0.526，3.738) |
| Cog_PC4 | 1.605(1.526，1.688)^*^ | 0.440(0.349，0.556)^*^ | 0.912(0.447，1.860) |
| Cog_PC5 | 0.876(0.809，0.950)^*^ | 1.530(1.253，1.869)^*^ | 1.086(0.640，1.845) |
| MRI_PC1 | 0.852(0.760，0.956)^*^ | 2.192(1.453，3.309)^*^ | 0.905(0.363，2.257) |
| MRI_PC2 | 0.742(0.673，0.818)^*^ | 2.878(1.444，5.734)^*^ | 1.181(0.171，8.148) |
| MRI_PC3 | 0.847(0.748，0.959)^*^ | 1.569(1.073，2.294)^*^ | 1.074(0.475，2.426) |
| MRI_PC4 | 0.999(0.986，1.011) | 1.016(0.982，1.051) | 1.005(0.954，1.059) |
| MRI_PC5 | 0.979(0.958，0.999)^*^ | 1.071(0.985，1.165) | 1.109(0.949，1.297) |

**Table 37 The observed and expected numbers at one-yearly intervals up to 15 years in the MSM 3, combining longitudinal markers from MSMs 1 and 2.**

| Years | Observed | | | Expected | | |
| --- | --- | --- | --- | --- | --- | --- |
|  | rNC | MCI | AD | rNC | MCI | AD |
| 0 | 0 | 1019 | 0 | 0.000 | 1019.000 | 0.000 |
| 1 | 32 | 832 | 115 | 29.671 | 840.284 | 109.045 |
| 2 | 42 | 561 | 234 | 47.587 | 609.170 | 180.243 |
| 3 | 45 | 366 | 278 | 49.381 | 423.635 | 215.983 |
| 4 | 39 | 238 | 317 | 45.962 | 308.381 | 239.656 |
| 5 | 36 | 156 | 329 | 40.428 | 225.901 | 254.671 |
| 6 | 38 | 122 | 338 | 38.183 | 186.484 | 273.333 |
| 7 | 35 | 98 | 341 | 34.027 | 153.305 | 286.669 |
| 8 | 28 | 74 | 347 | 30.109 | 124.269 | 294.622 |
| 9 | 16 | 54 | 353 | 23.791 | 98.811 | 300.398 |
| 10 | 11 | 39 | 356 | 19.773 | 81.417 | 304.811 |
| 11 | 7 | 20 | 359 | 12.741 | 64.262 | 308.997 |
| 12 | 3 | 8 | 359 | 7.911 | 50.714 | 311.376 |
| 13 | 2 | 4 | 359 | 6.446 | 44.107 | 314.447 |
| 14 | 2 | 1 | 359 | 5.783 | 38.624 | 317.593 |
| 15 | 2 | 1 | 359 | 5.613 | 35.104 | 321.283 |

**Figure 1 Observed curves of cognitive assessments for different MCI types. Cognition and daily function in rMCI individuals remained generally at a better level, while function in pMCI individuals deteriorated sharply.**


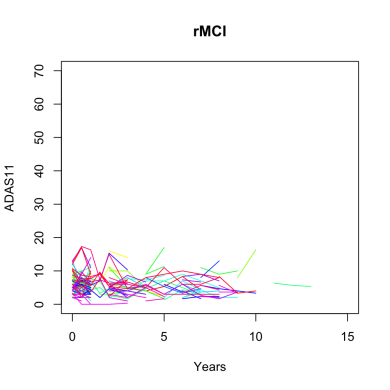

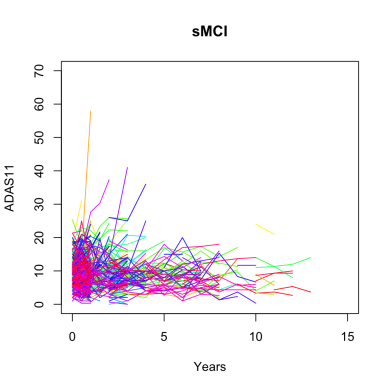

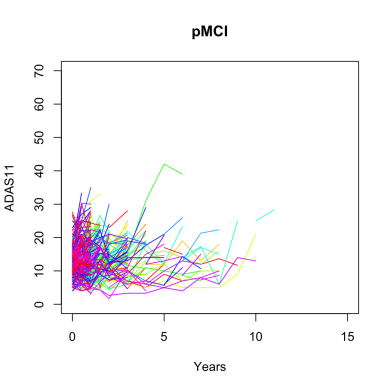


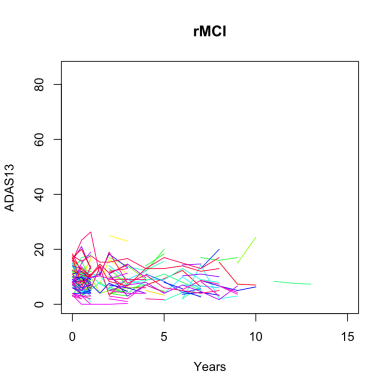

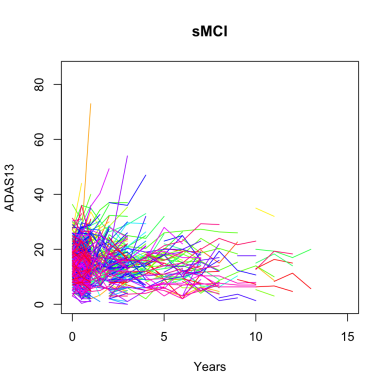

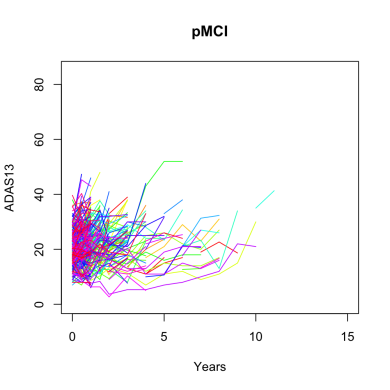


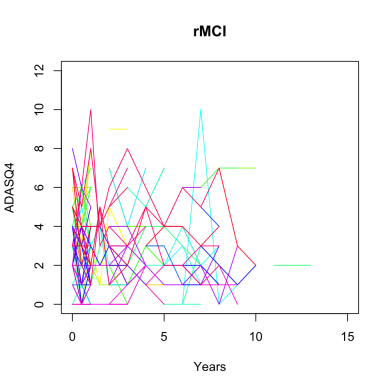

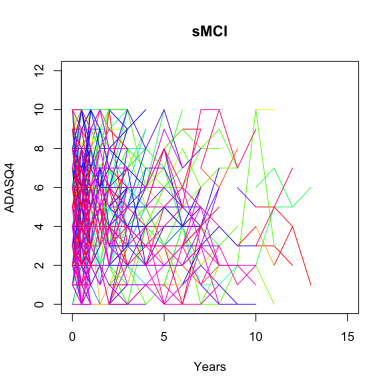

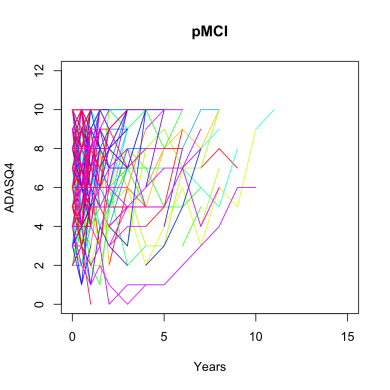


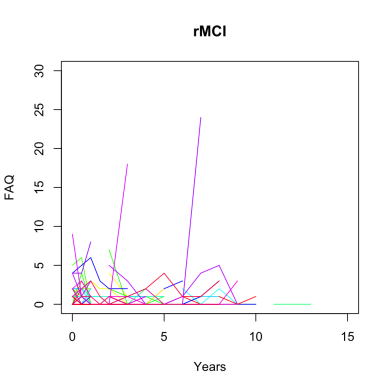

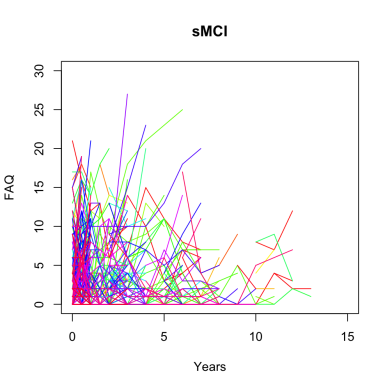

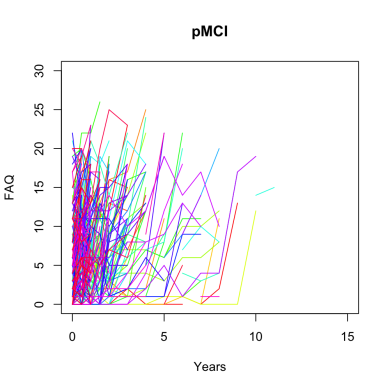


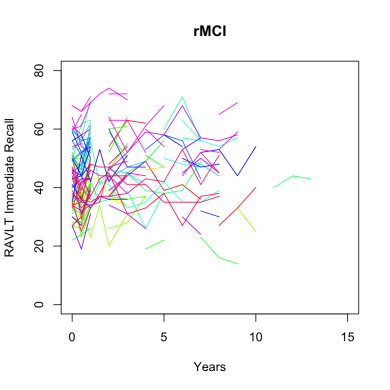

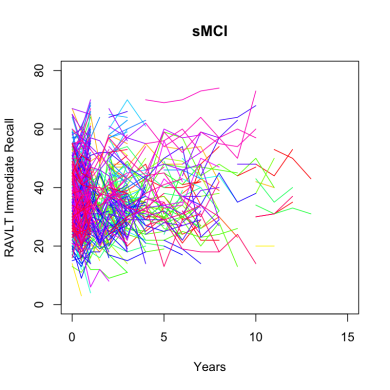

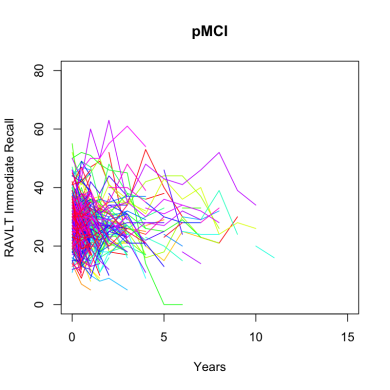


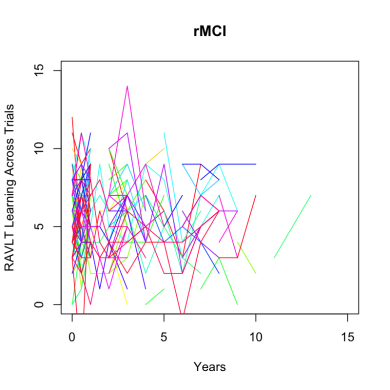

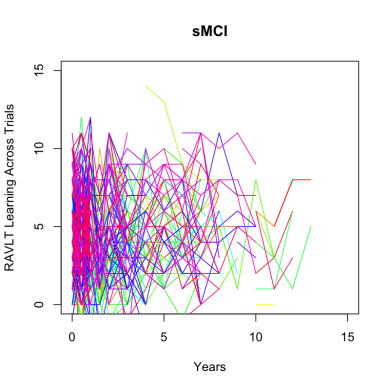

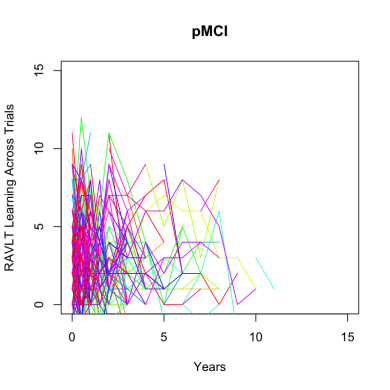


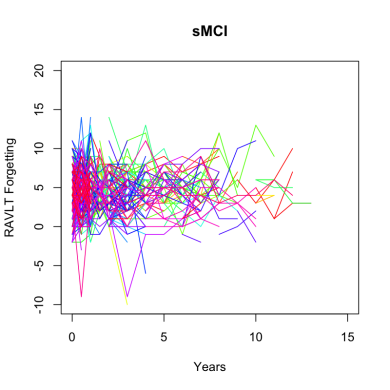

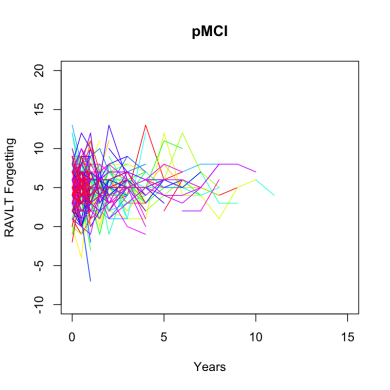

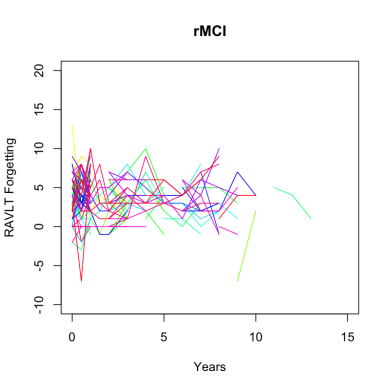


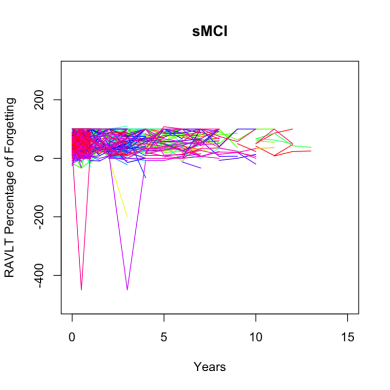

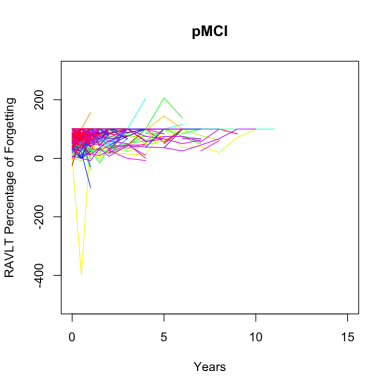

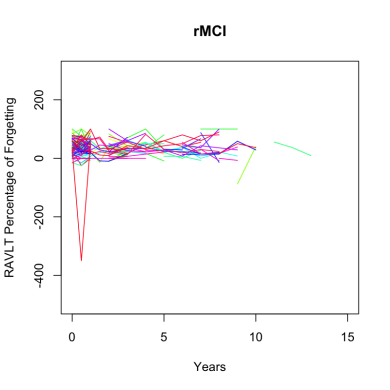


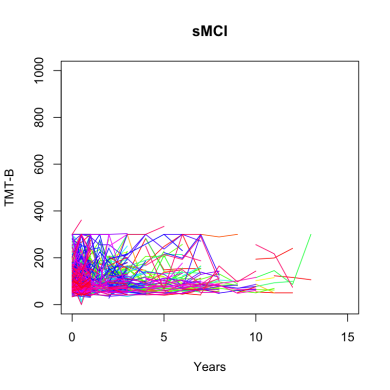

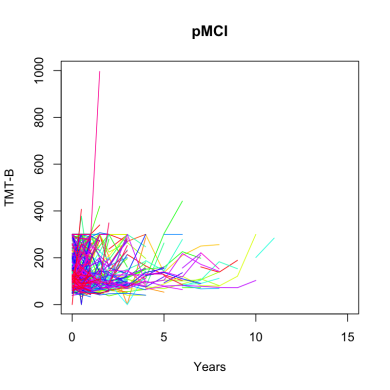

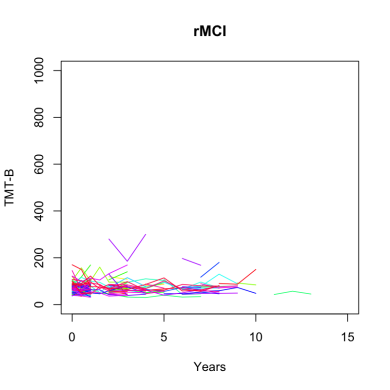


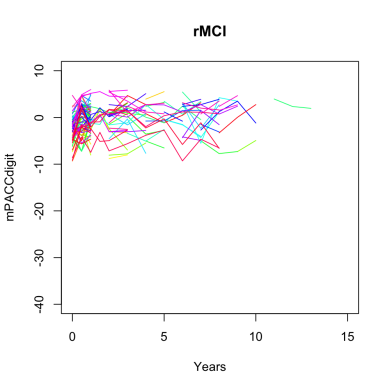

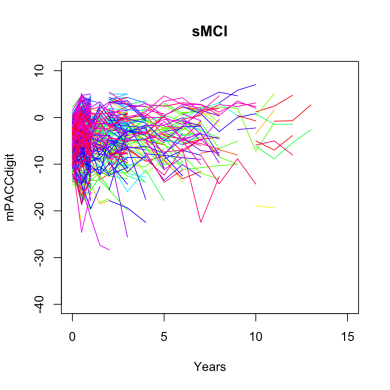

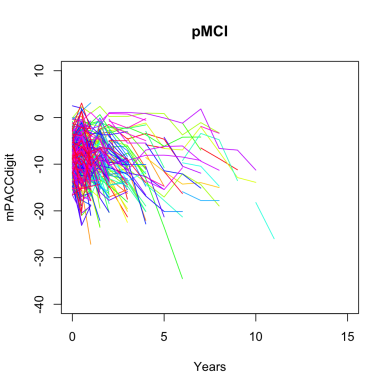


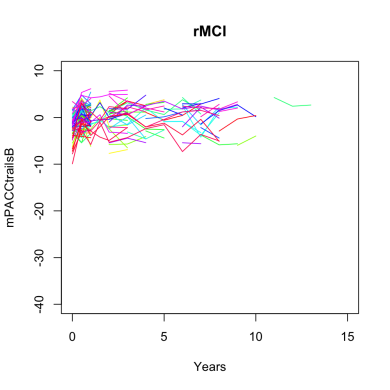

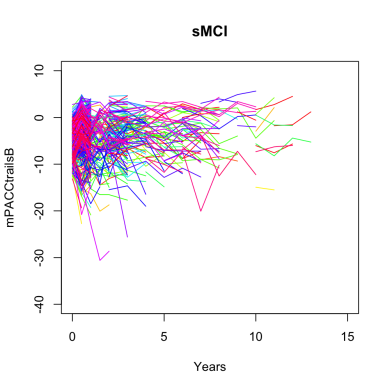

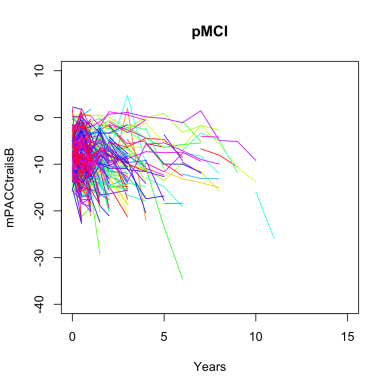


**Figure 2 Scree plots of the FPCs extracted for each longitudinal cognitive assessment using univariate FPCA, when setting the cumulative PVE at 90%.**

**
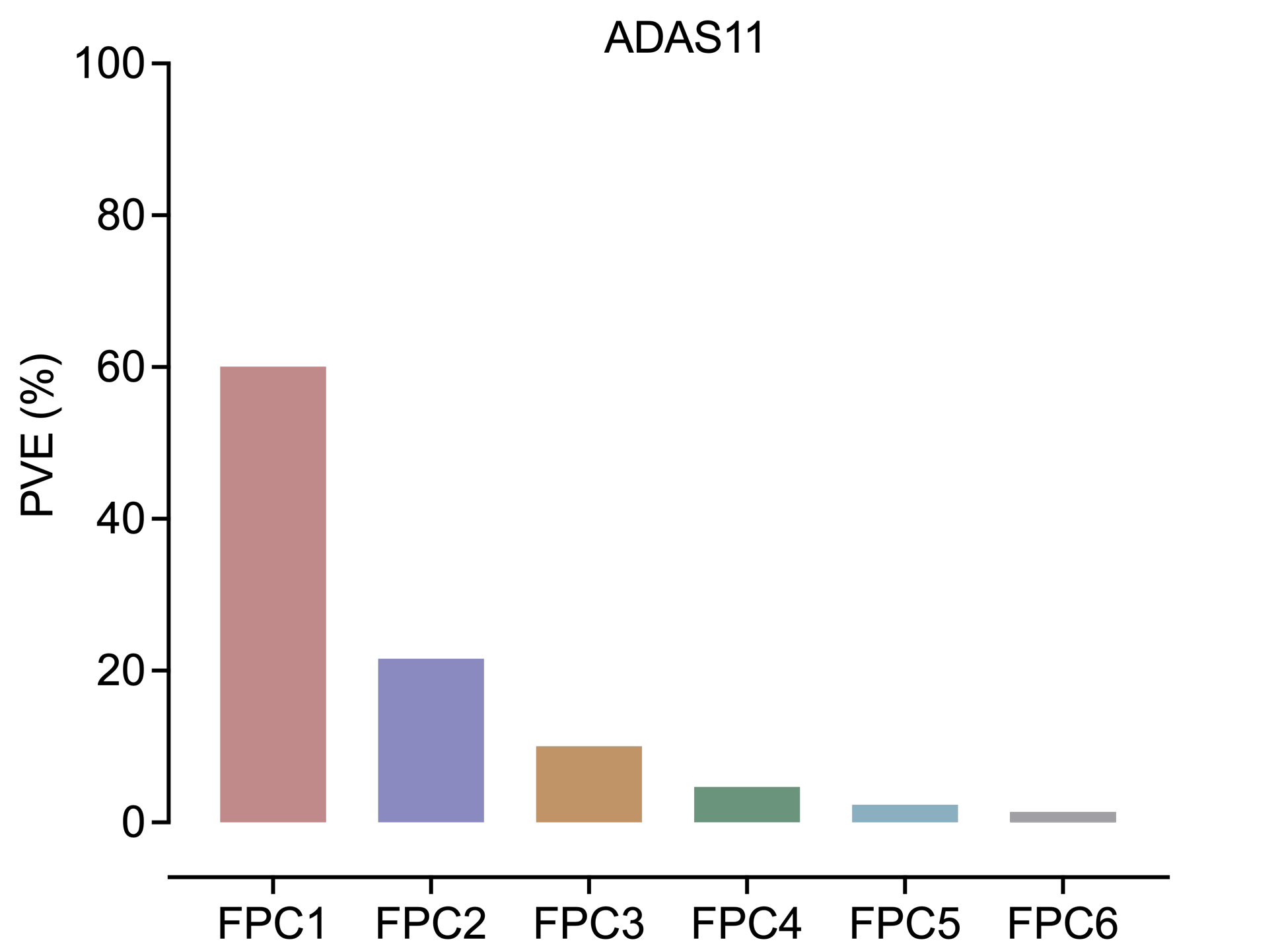

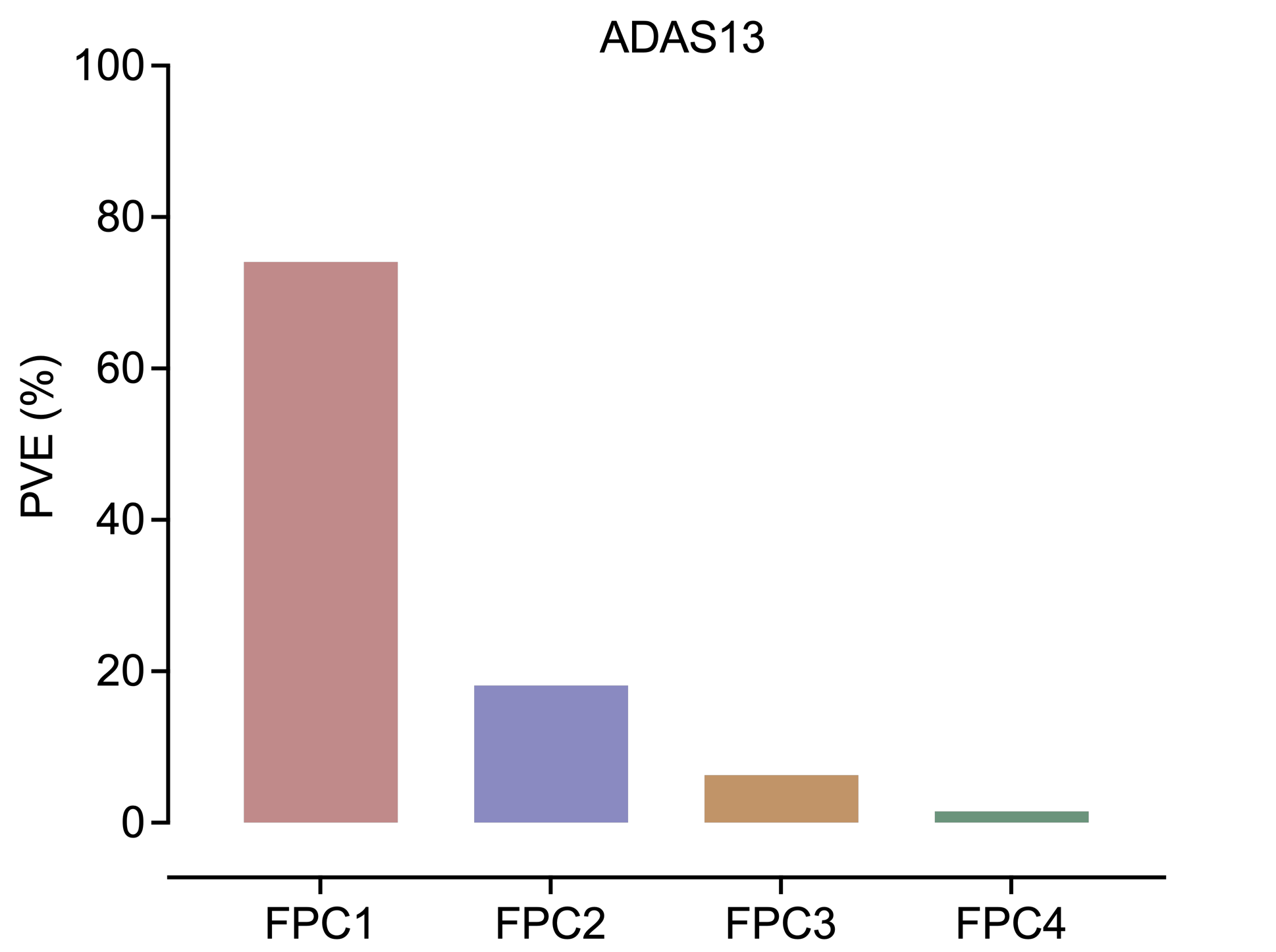

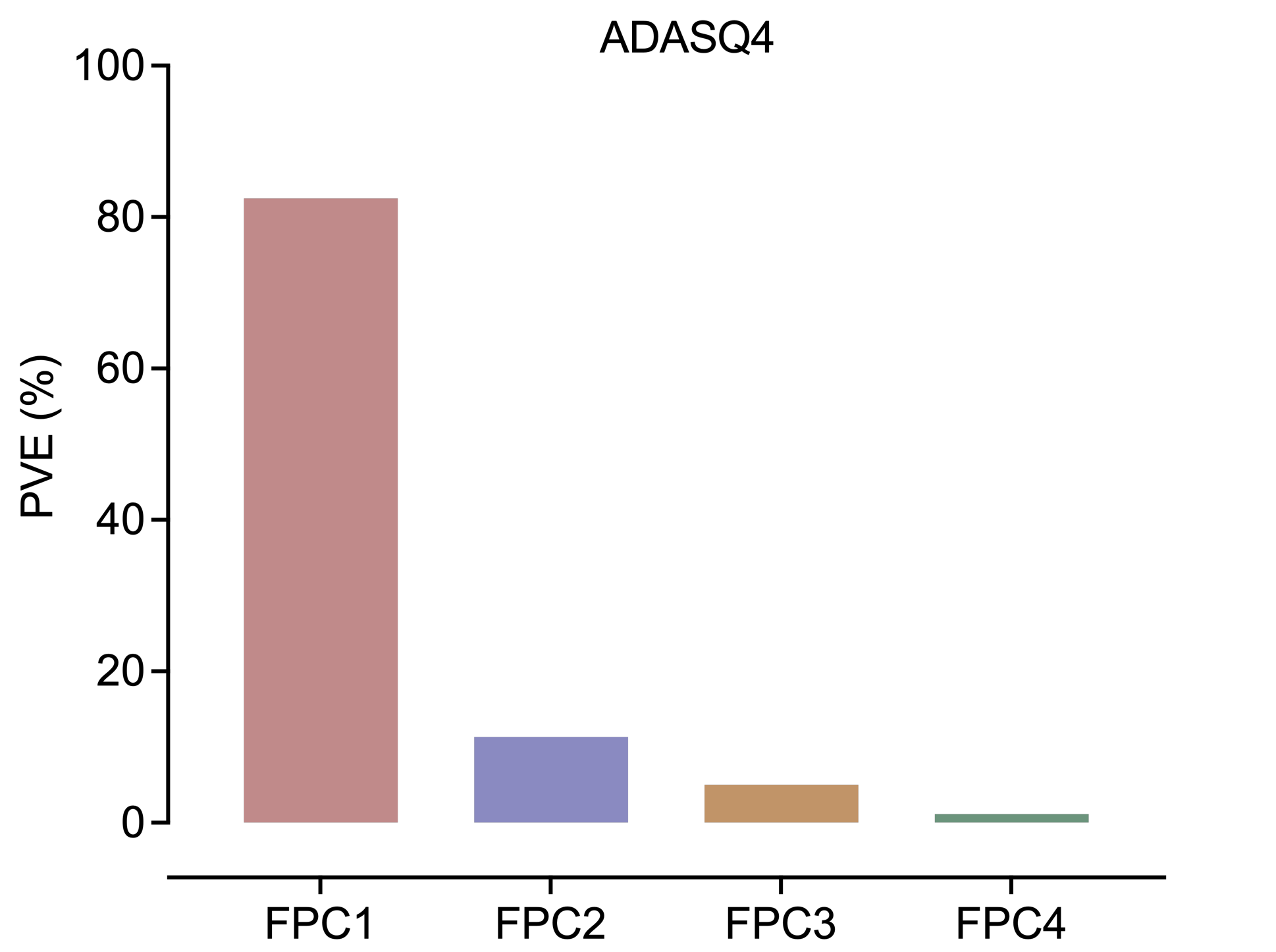
**

**
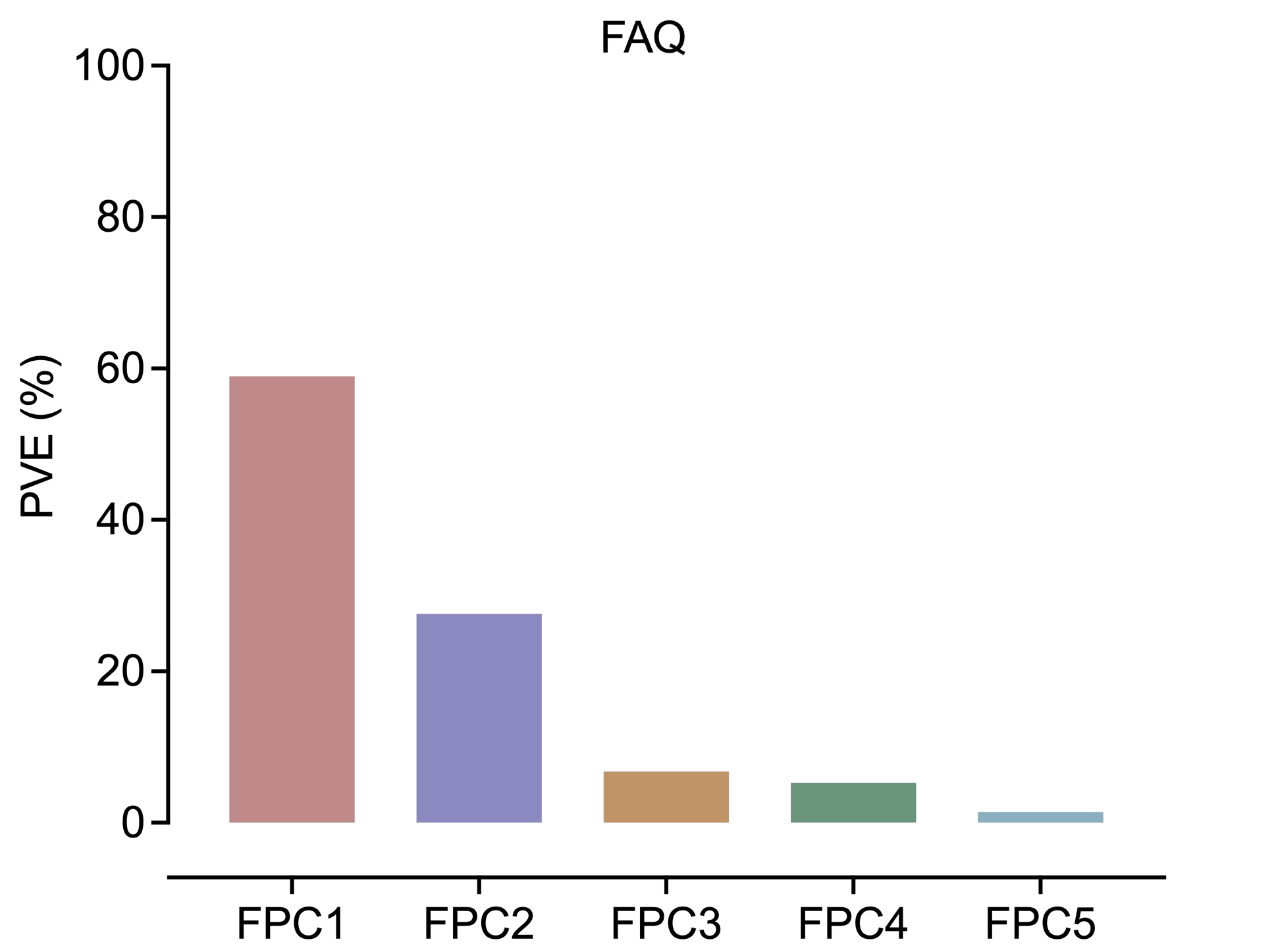

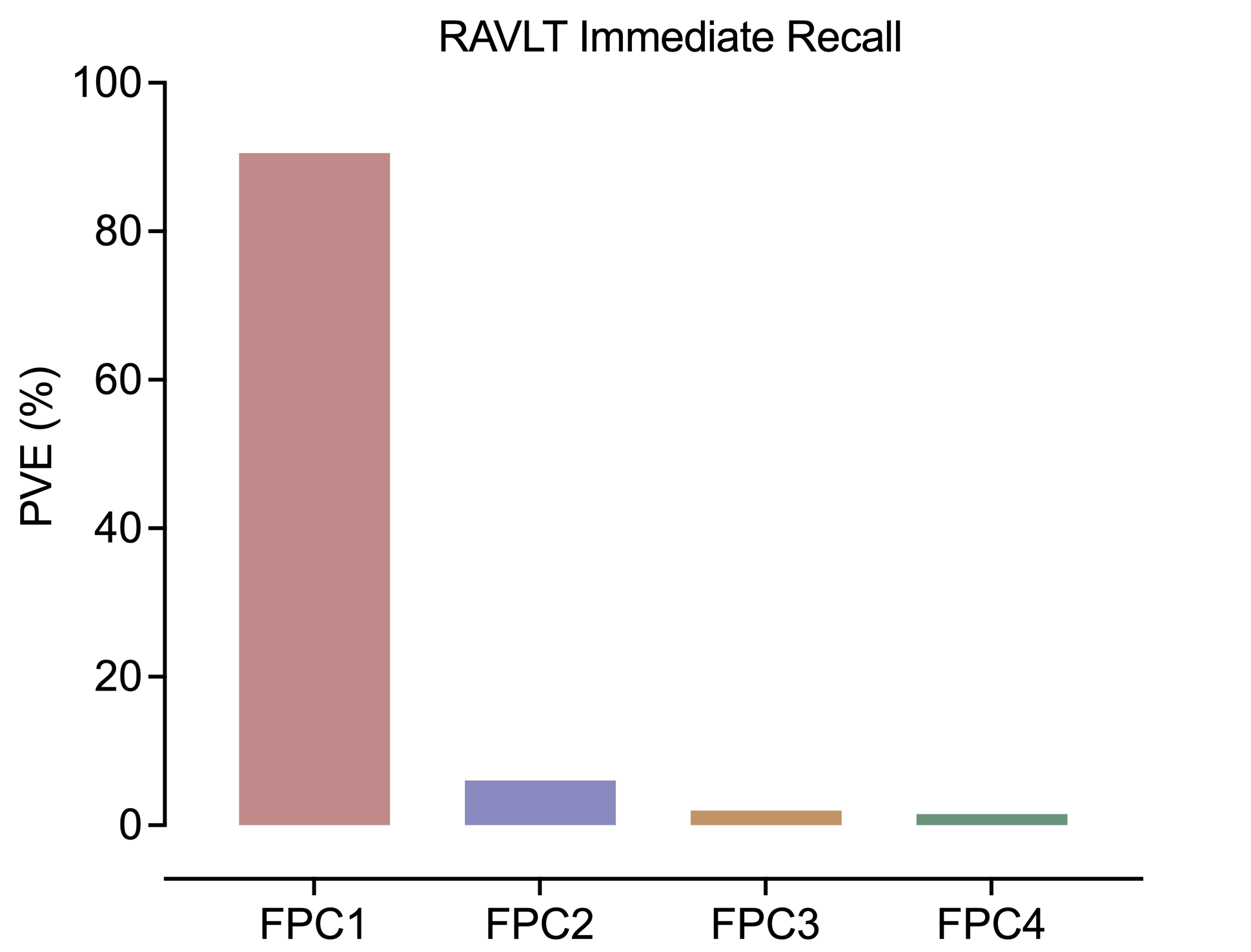

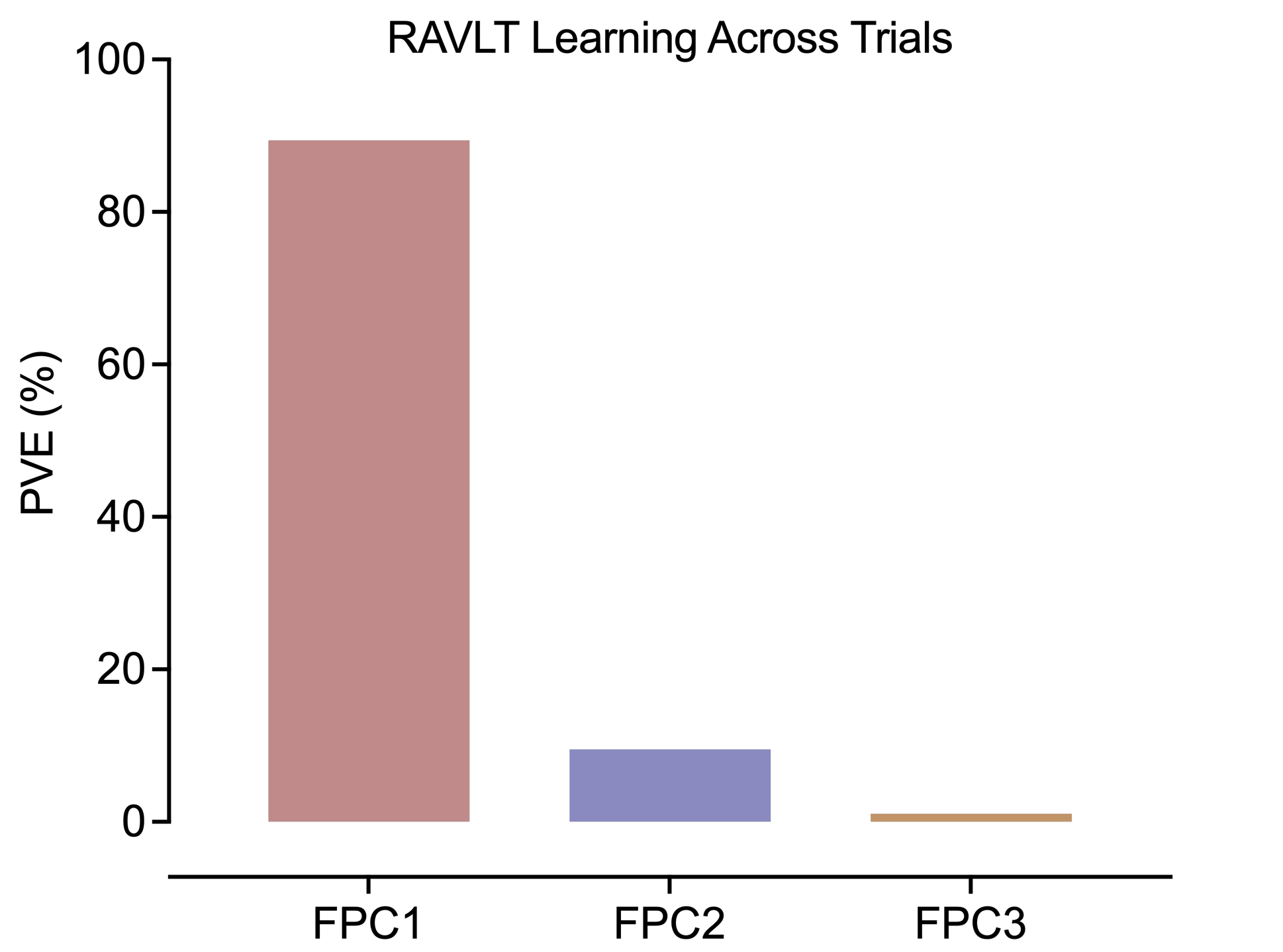
**

**
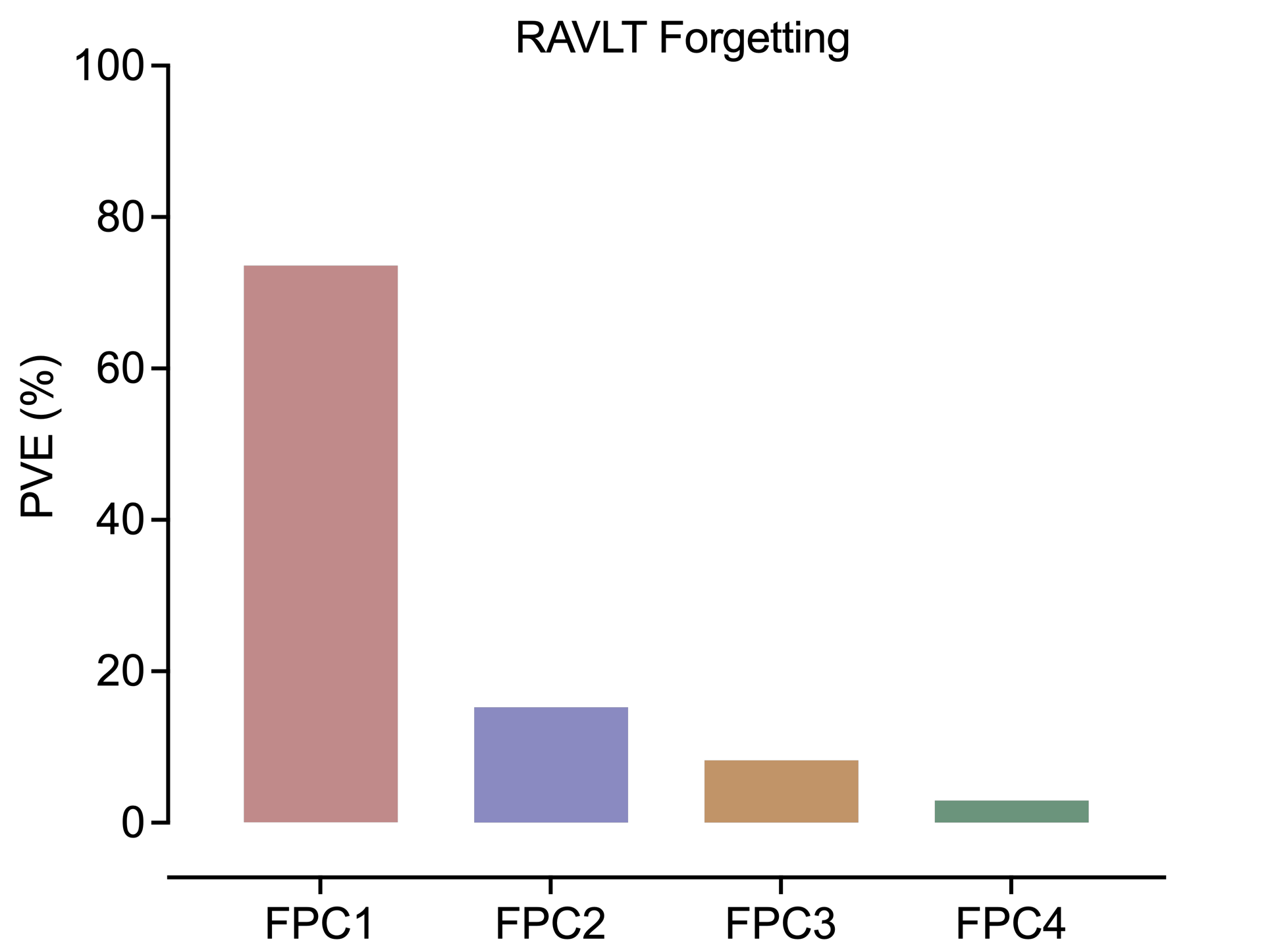

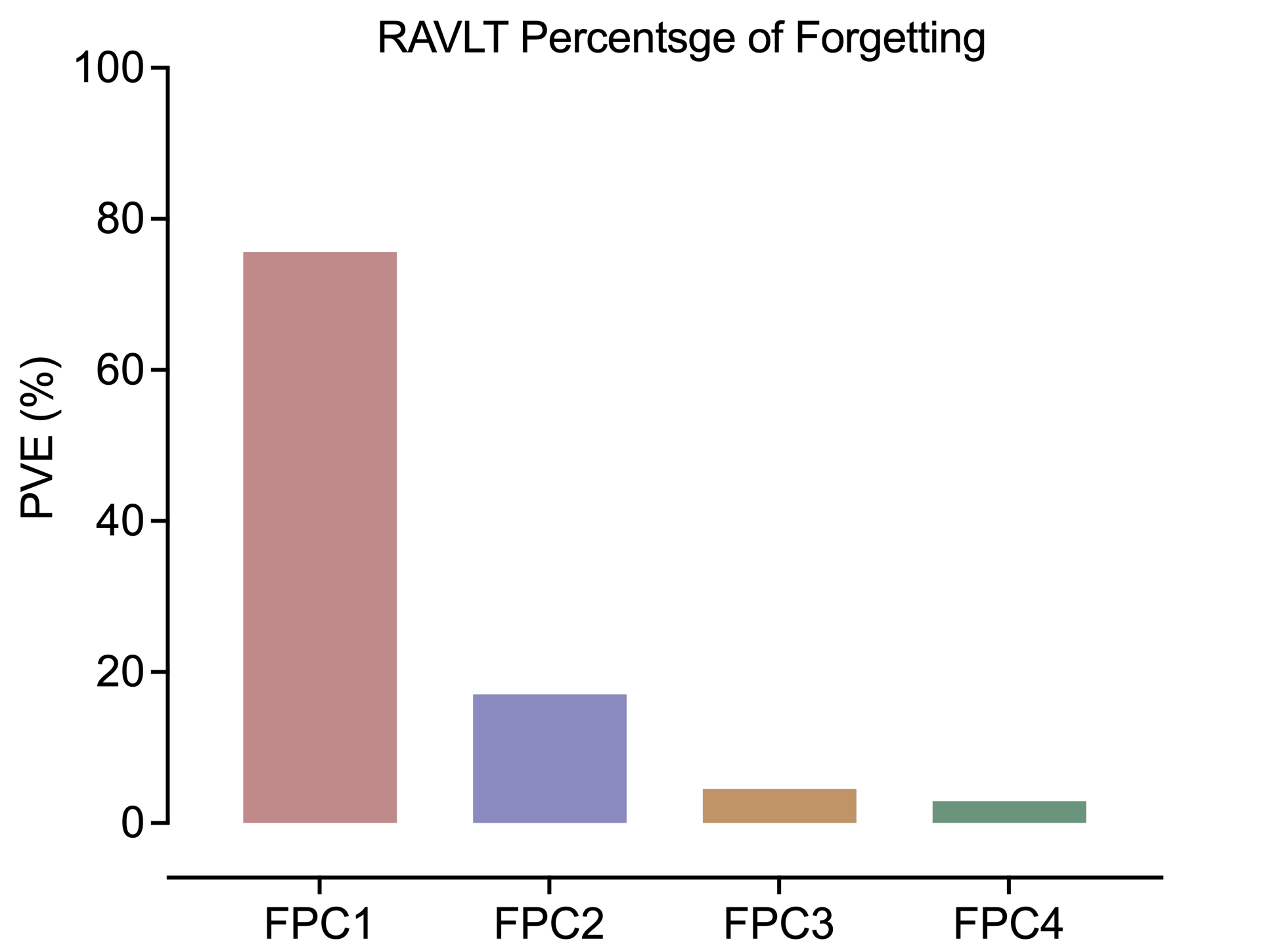

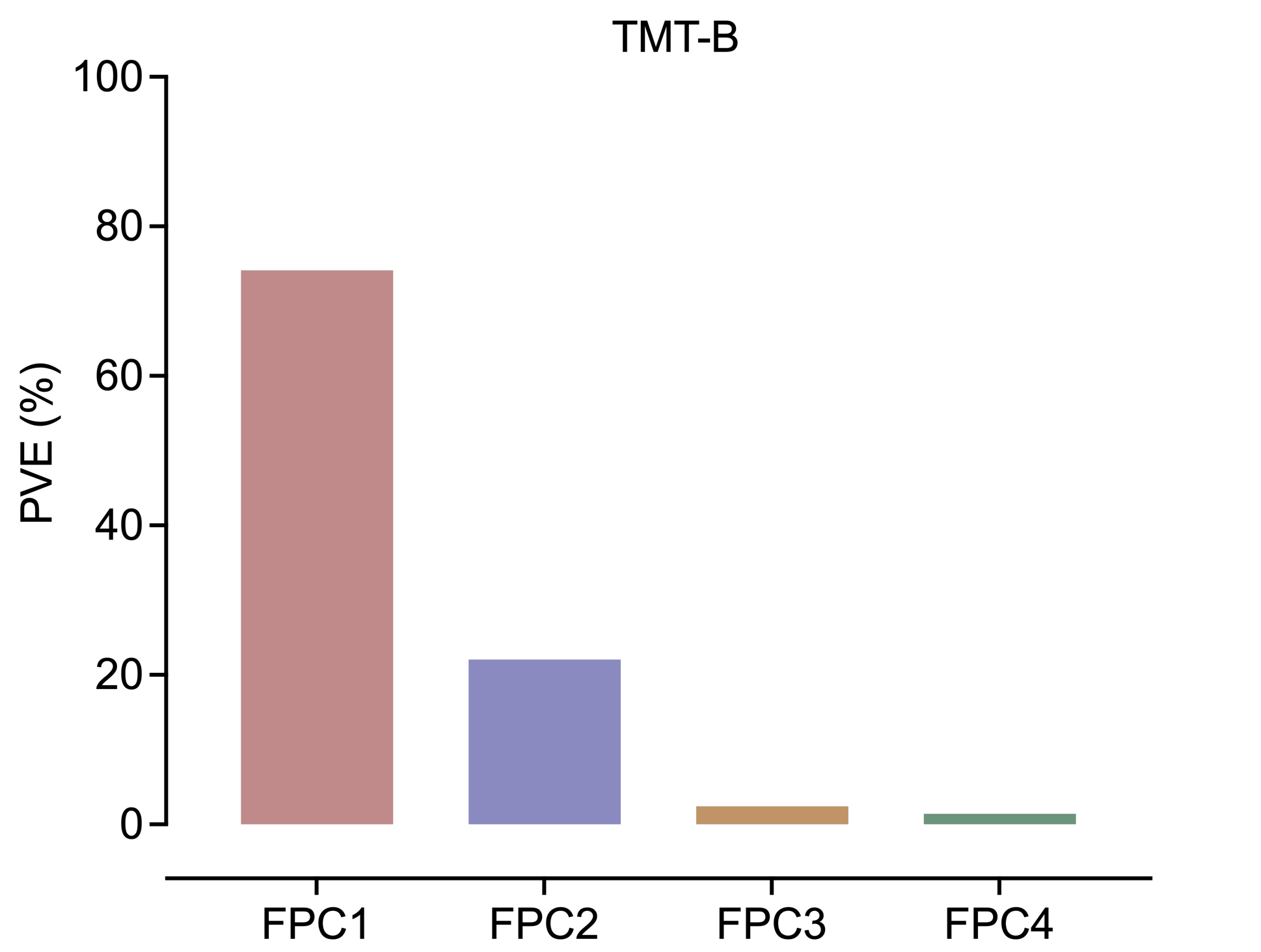
**

**
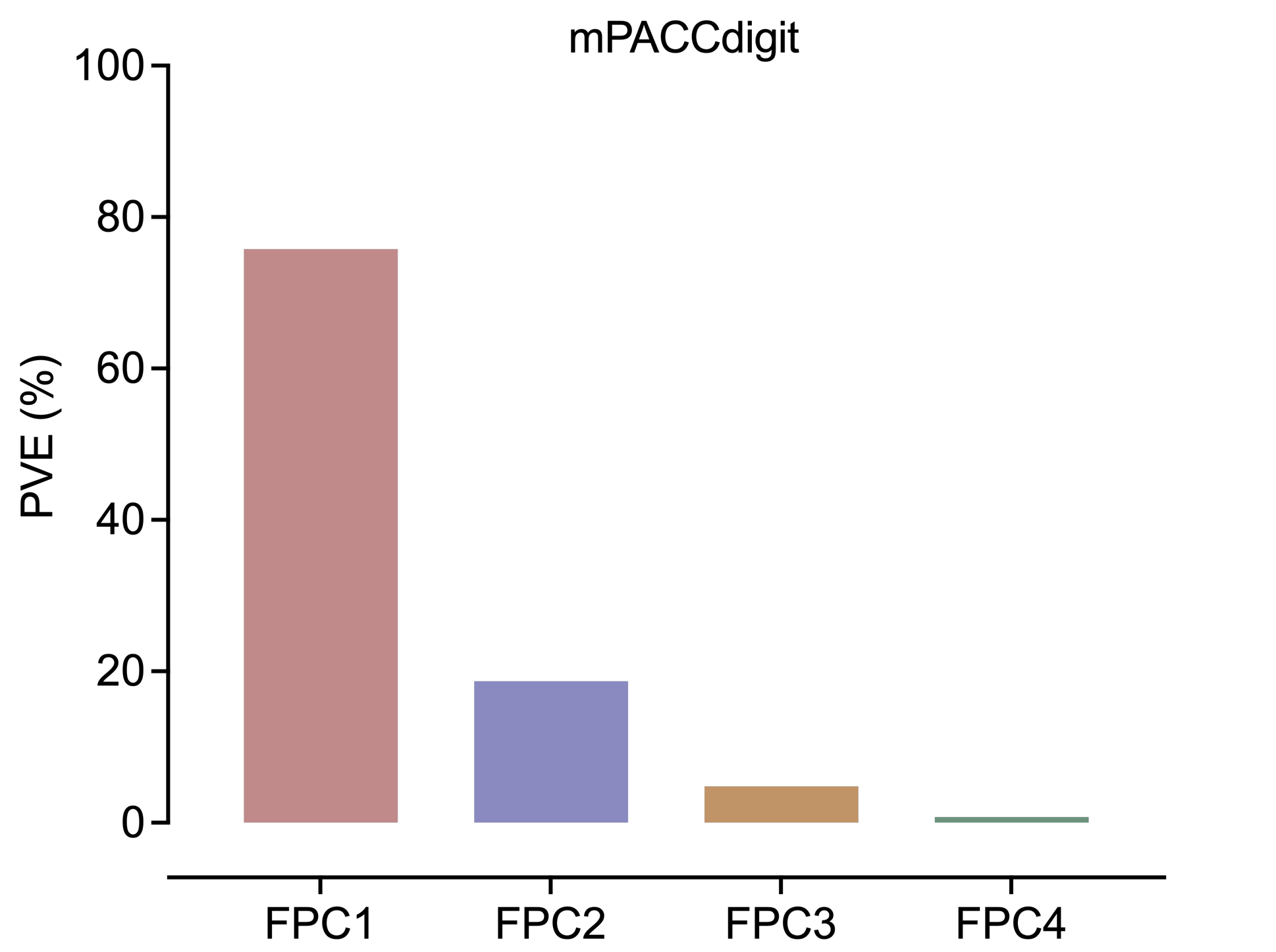

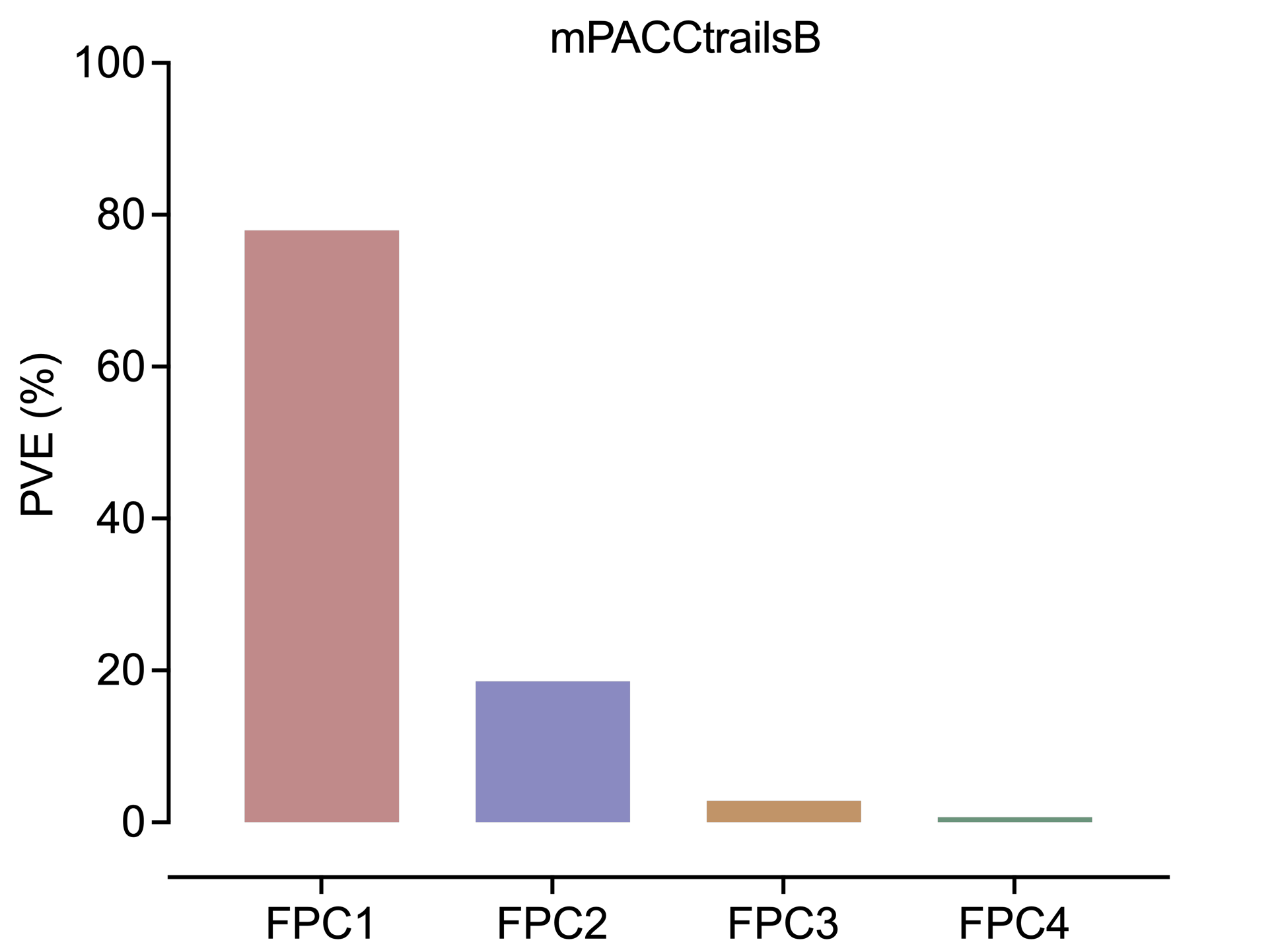
**

**Figure 3 Mean smoothing curves (the first column) and eigenfunction curves (the second column) and observations (circles) and predicted trajectories (red dashed lines) for the two individual with the largest projections on the respective eigenfunctions above, overlaid with the overall estimated mean function (solid lines) (the second and third column), for each longitudinal cognitive assessment obtained by the univariate FPCA.**


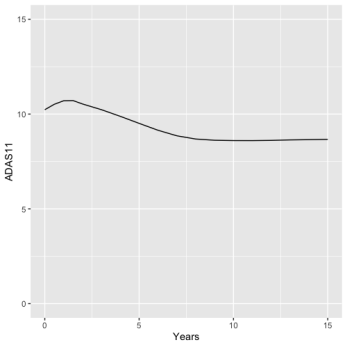

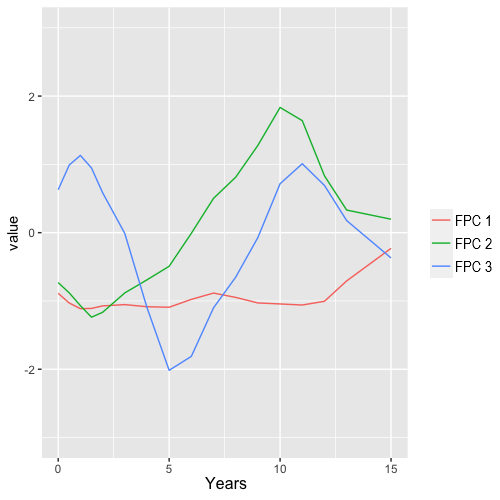

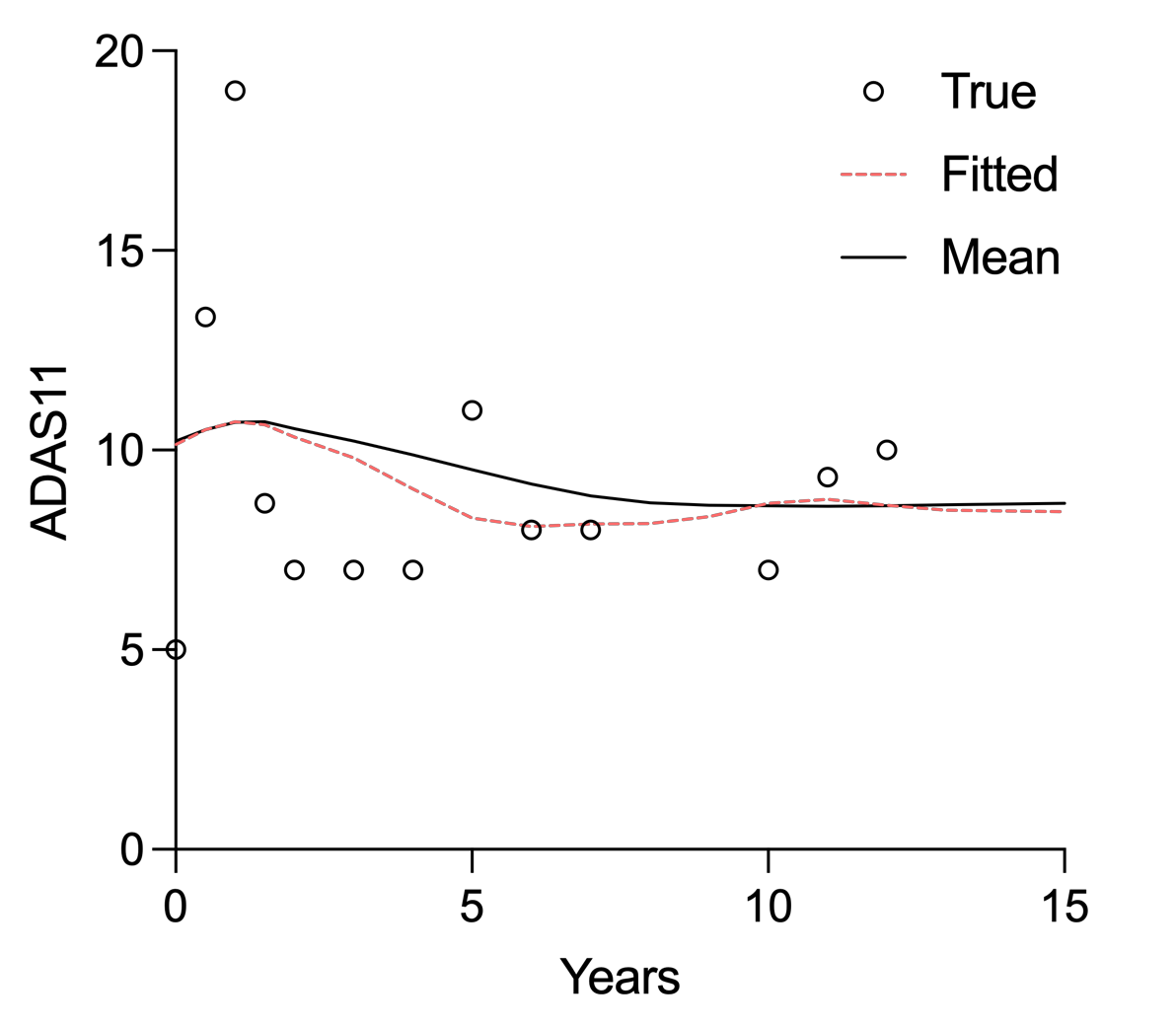

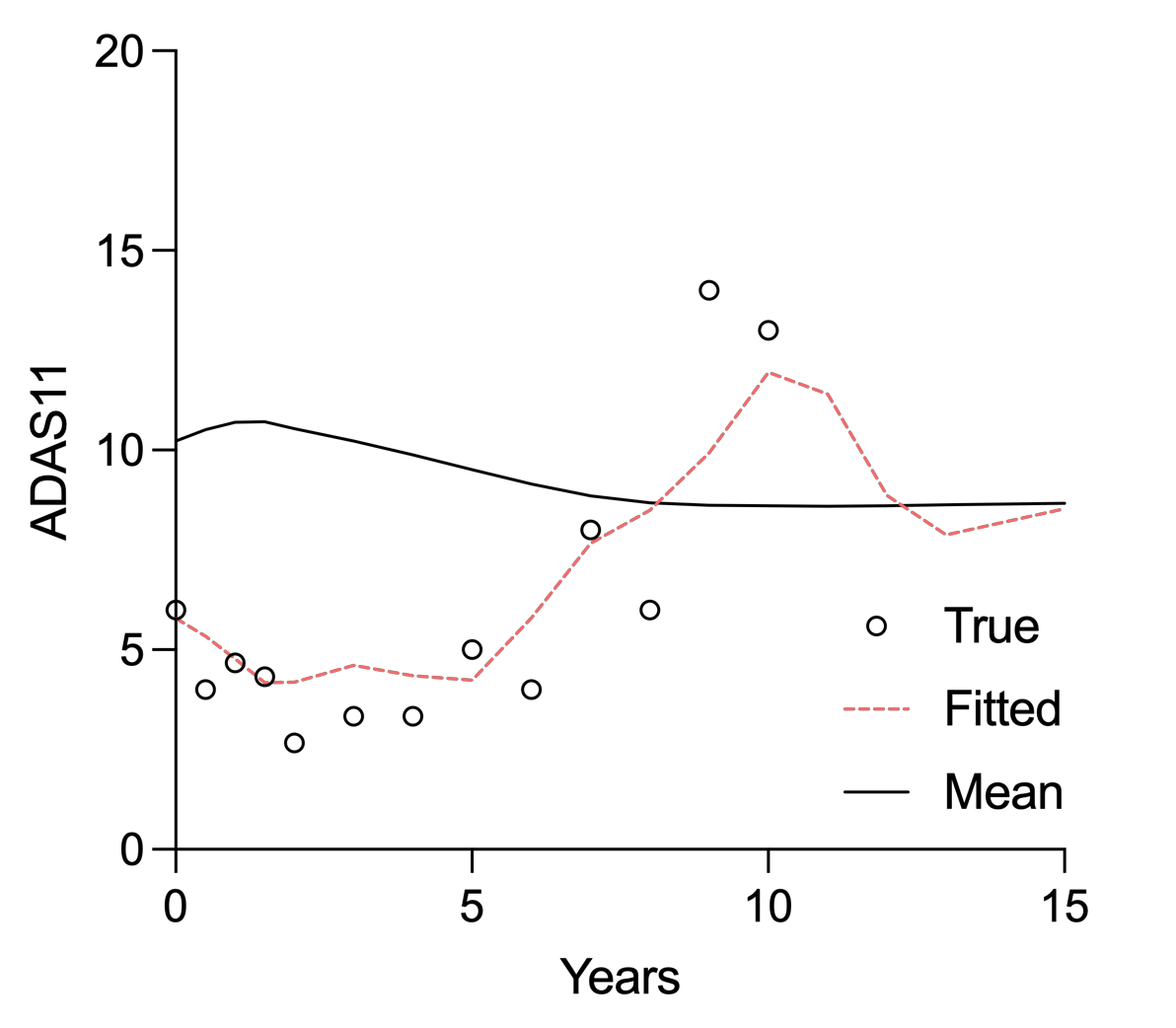


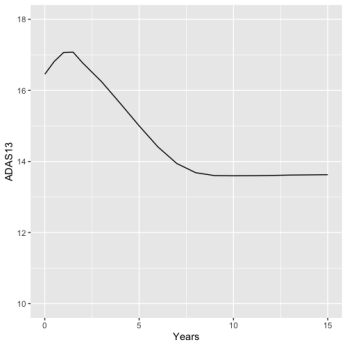

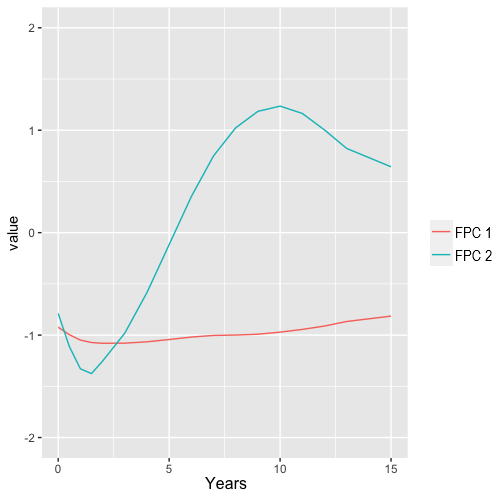

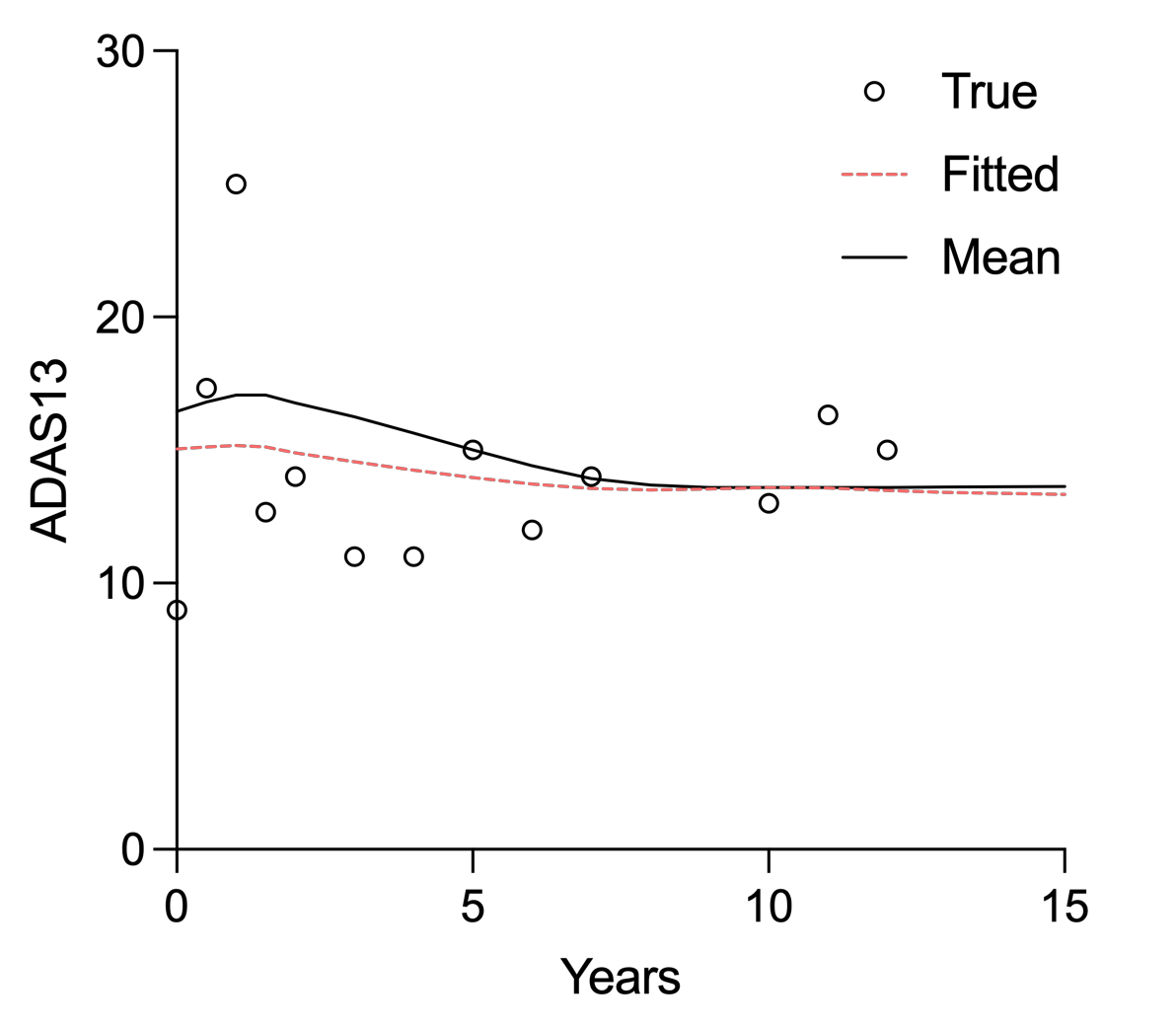

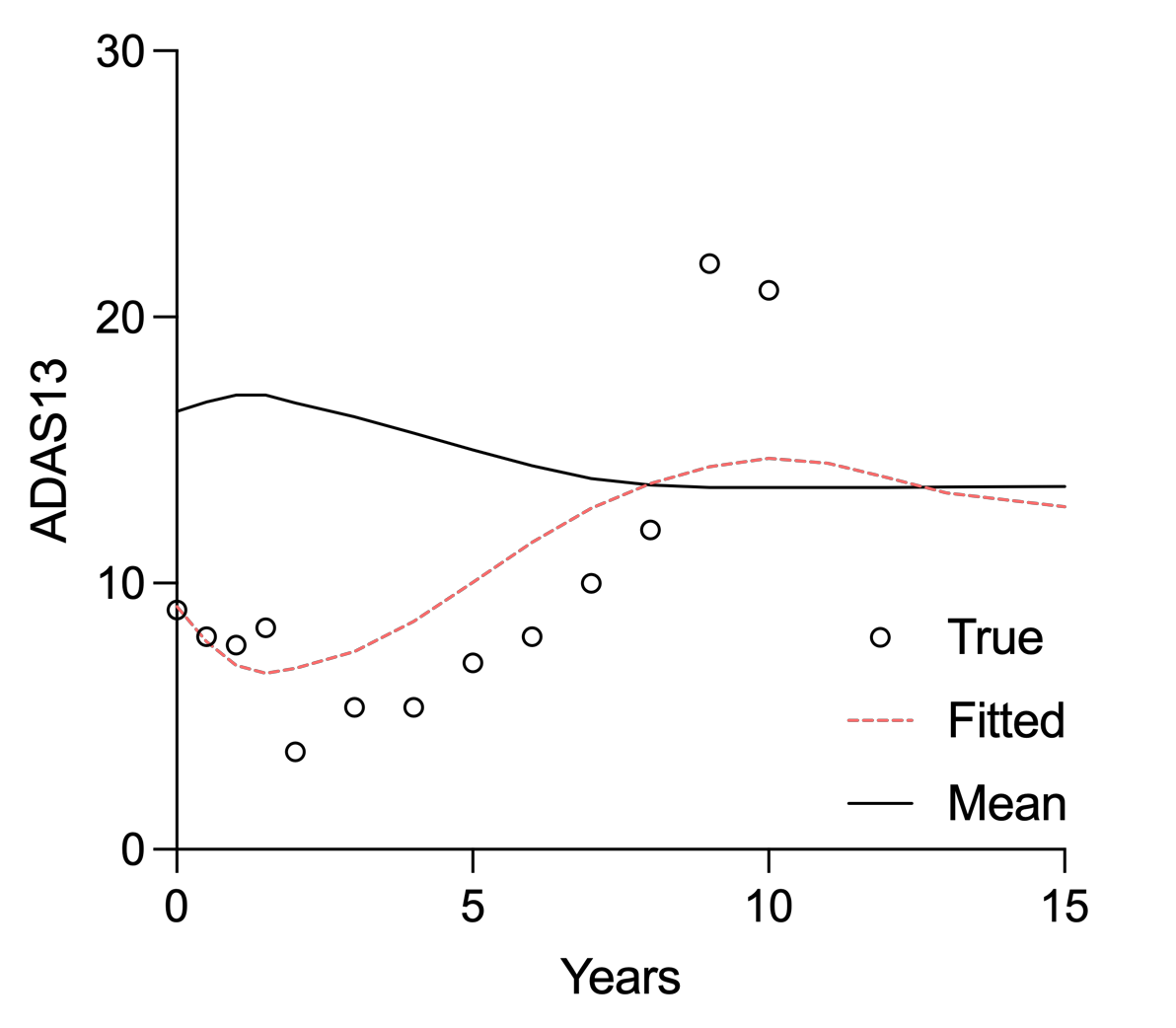


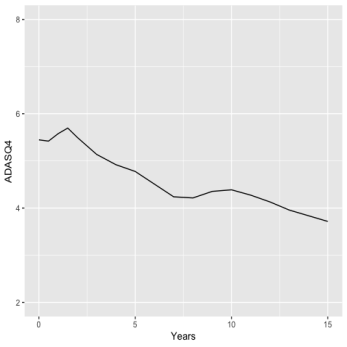

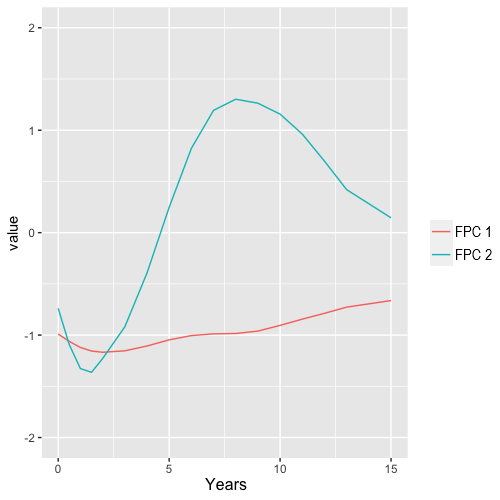

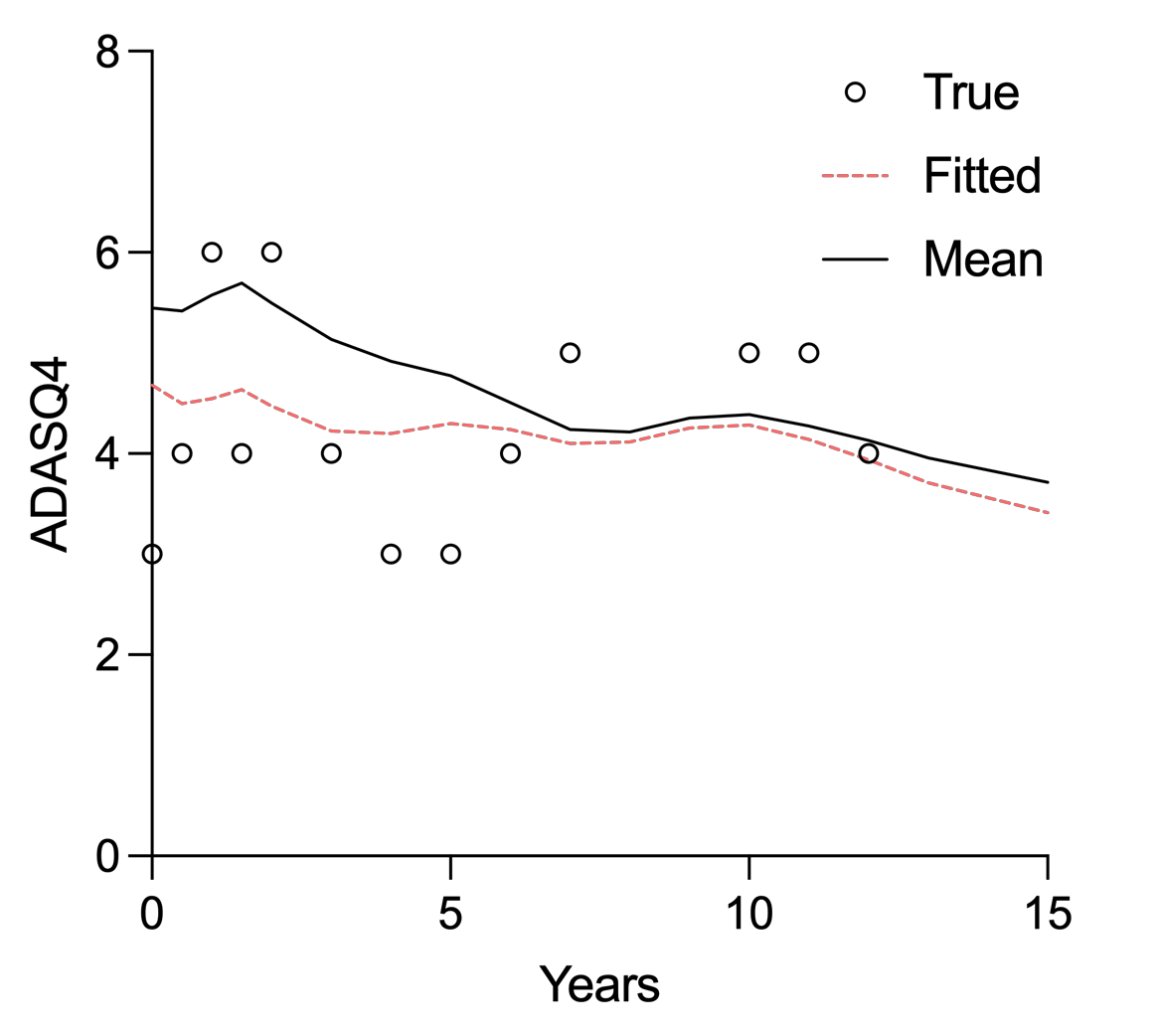

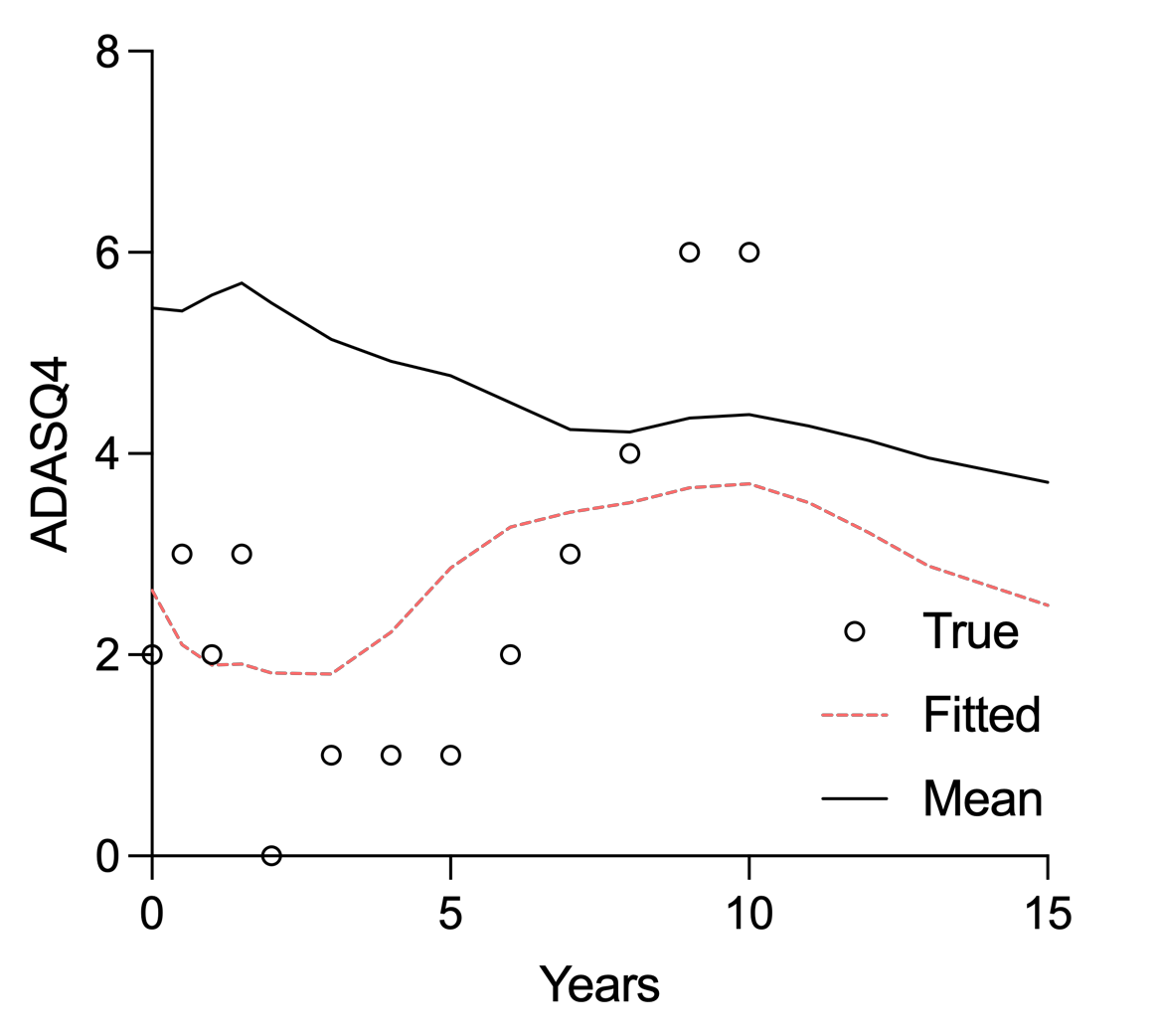


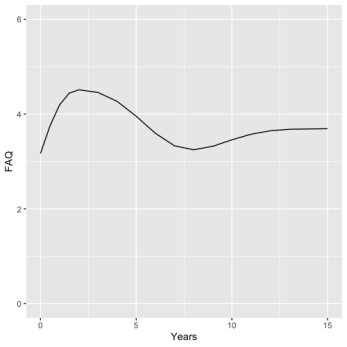

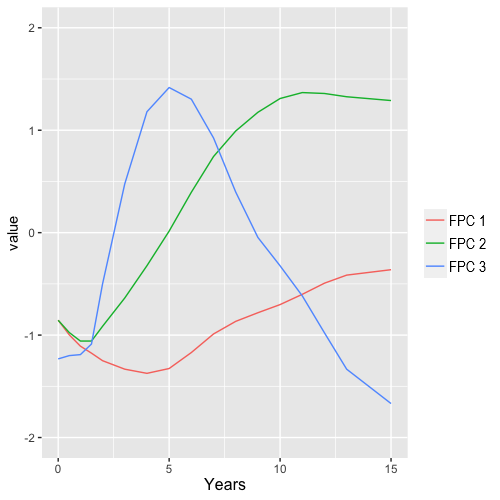

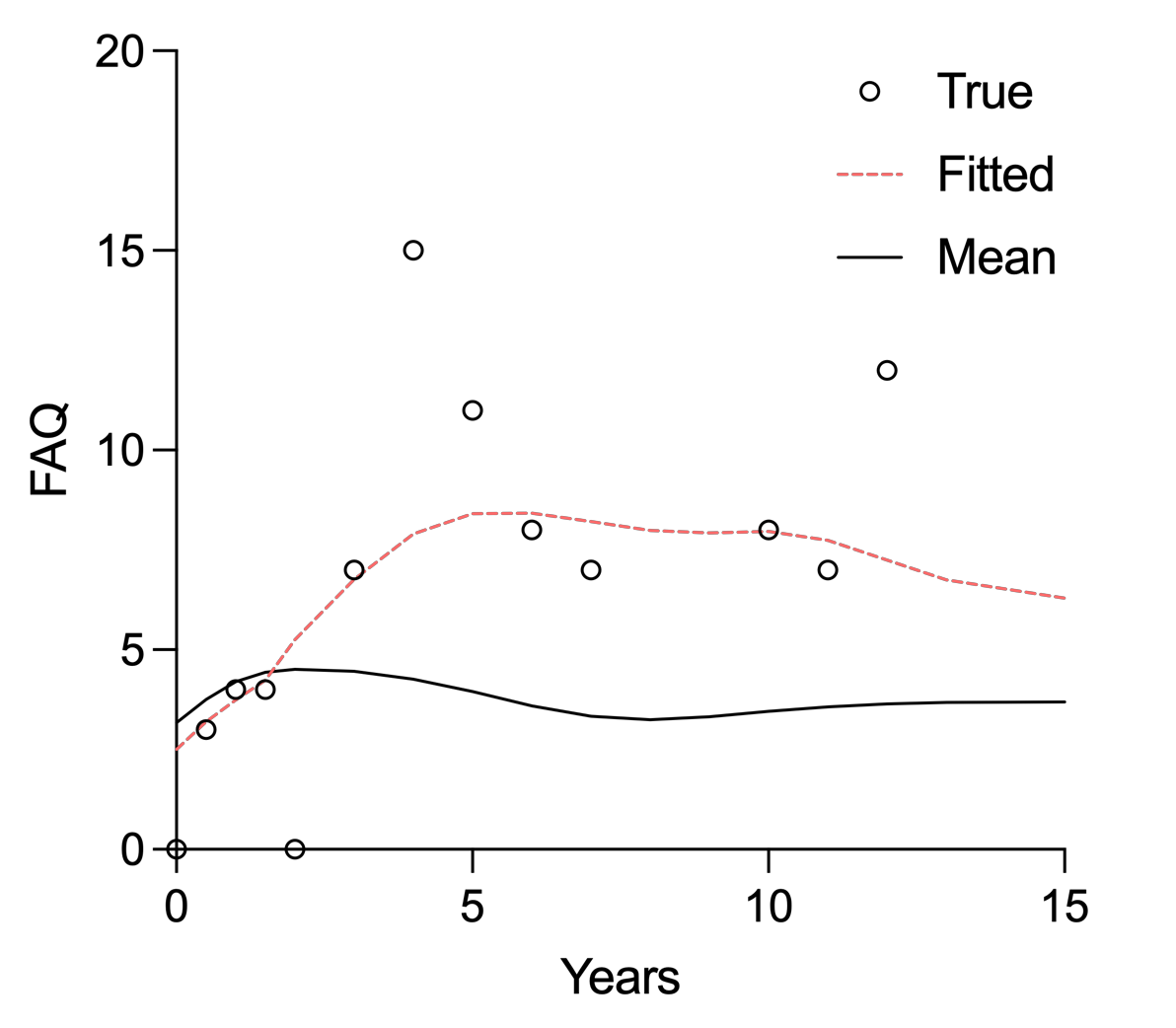

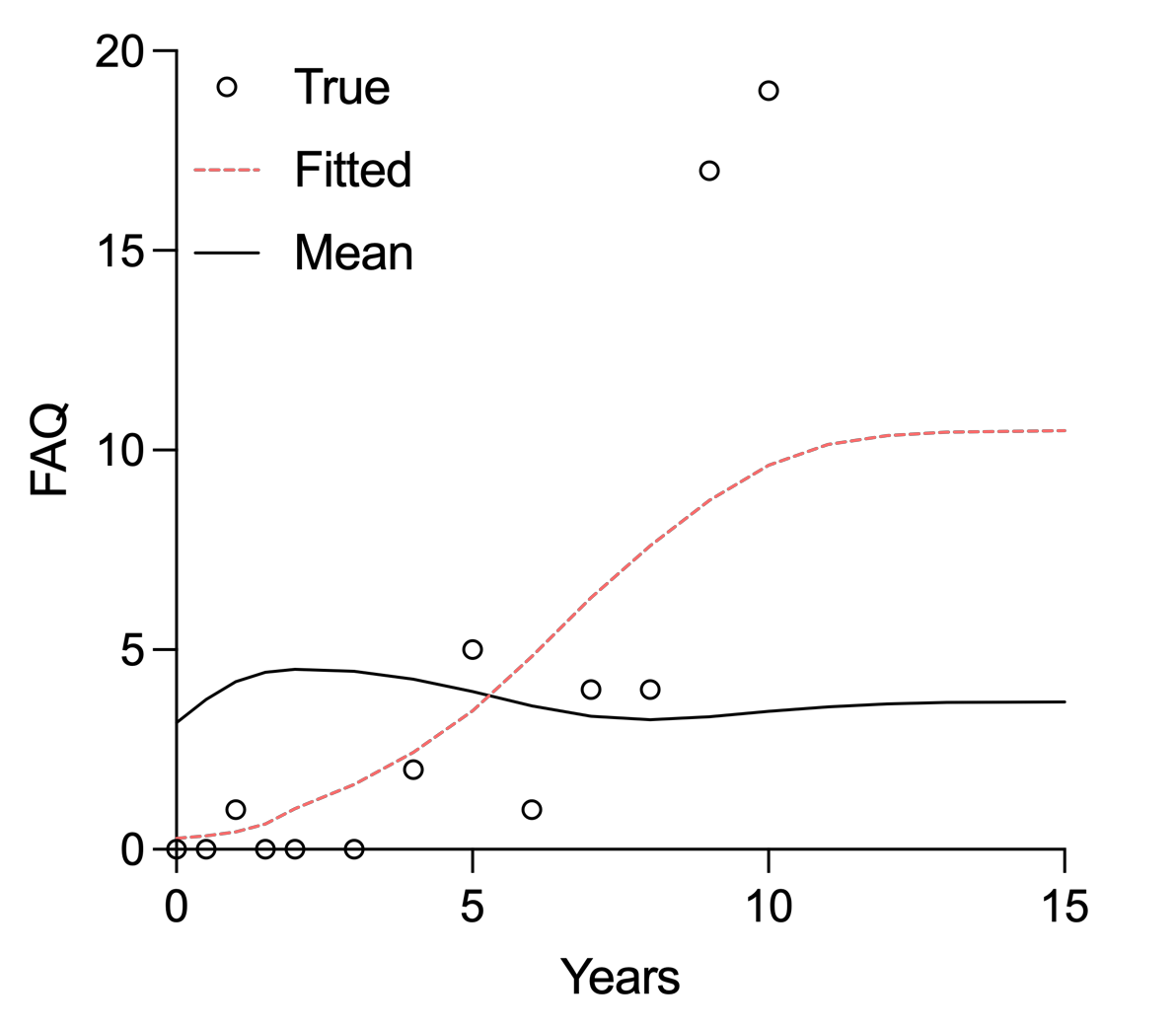


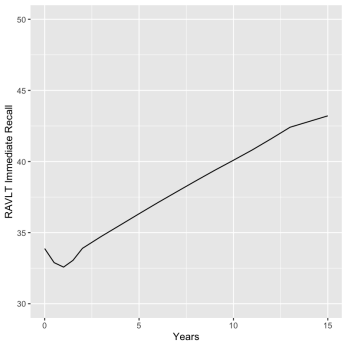

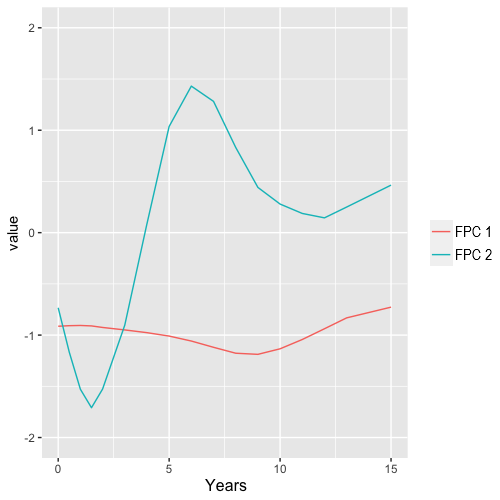

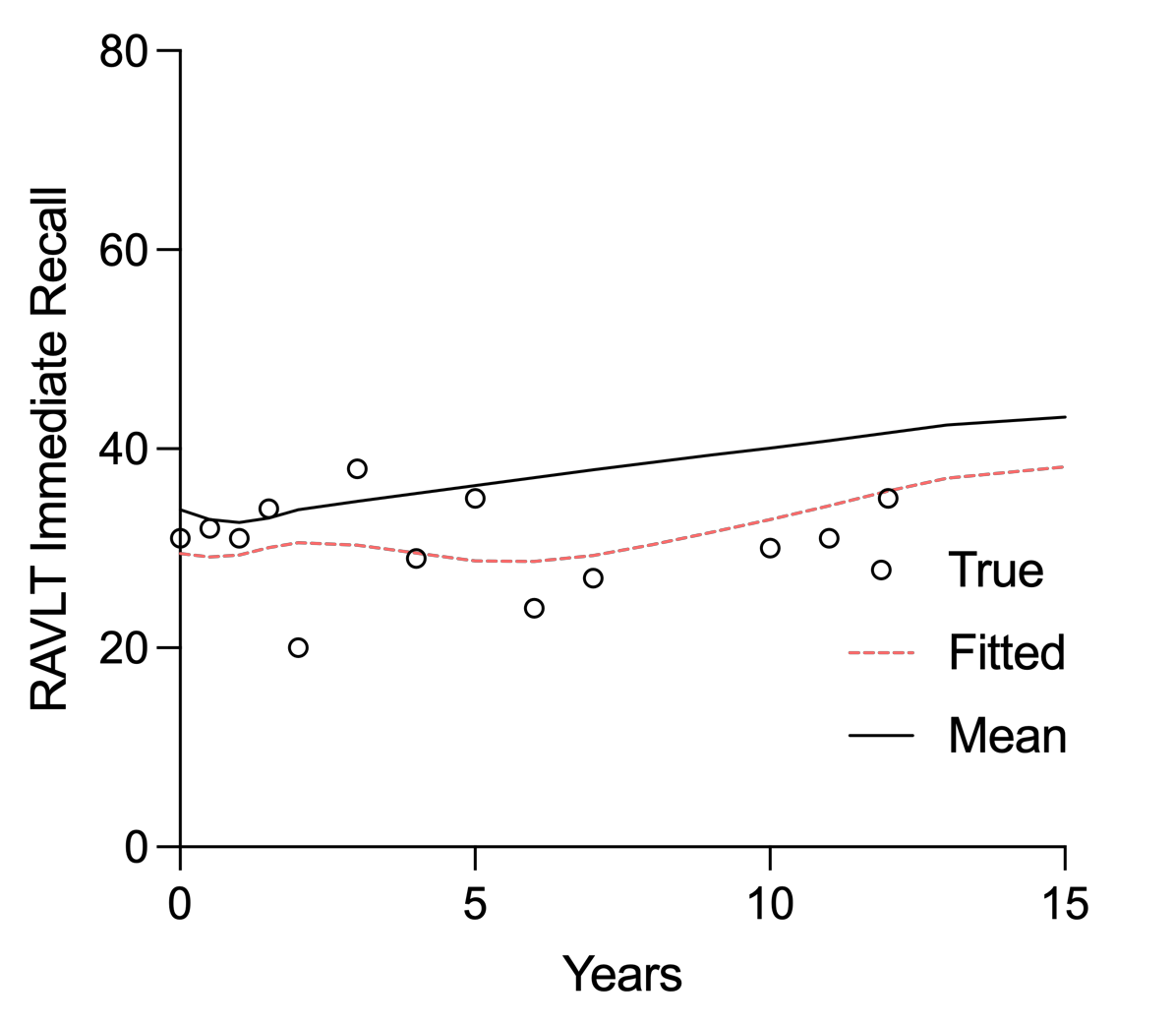

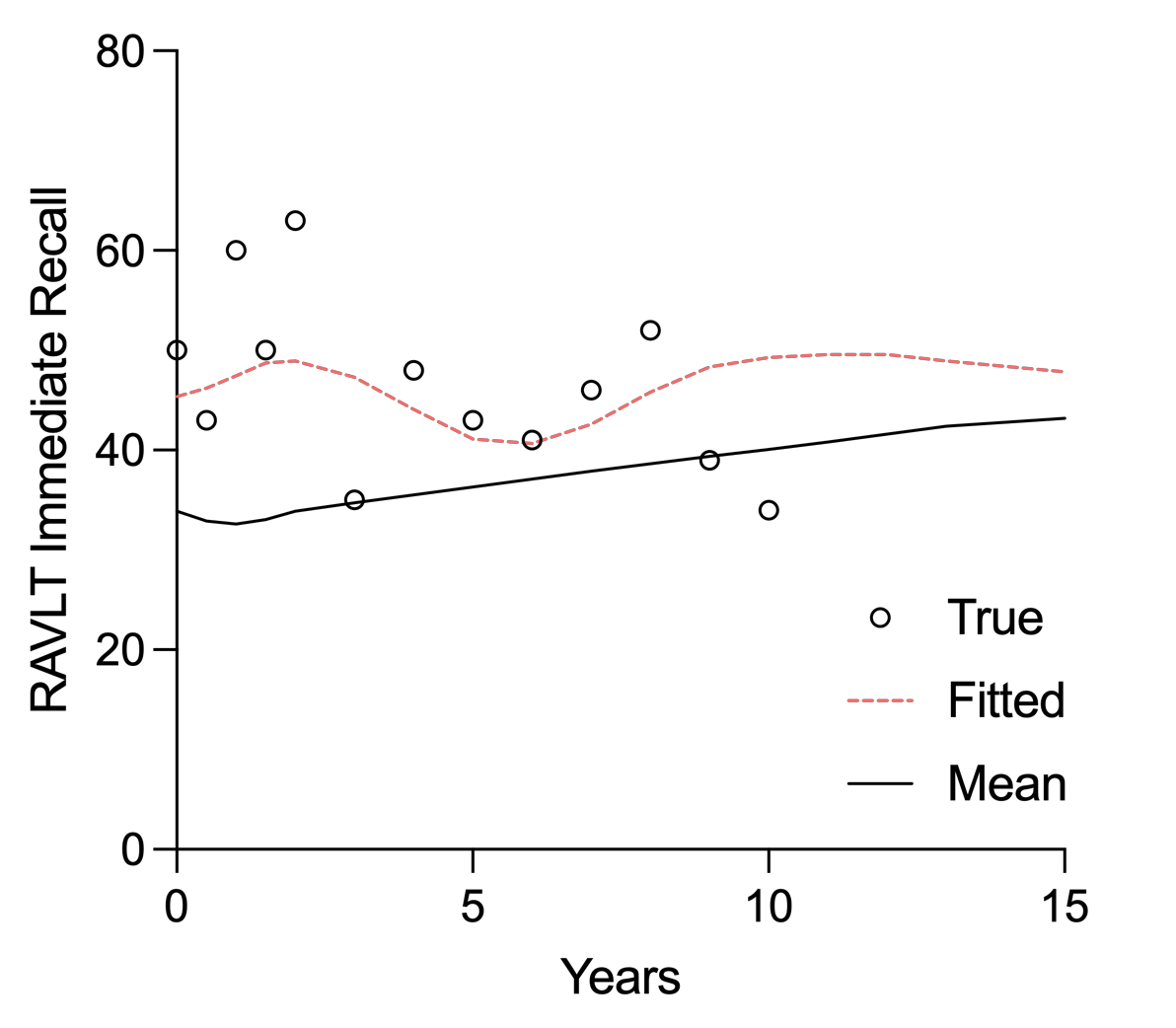


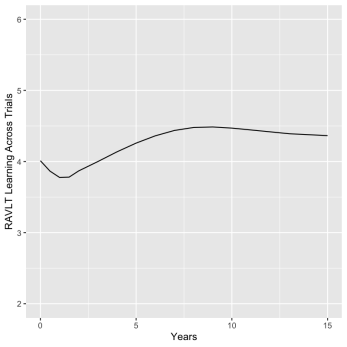

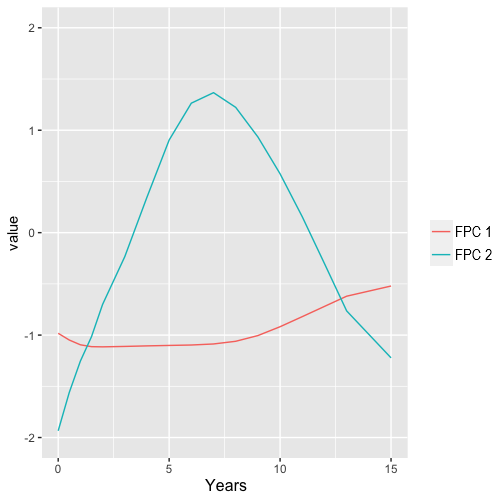

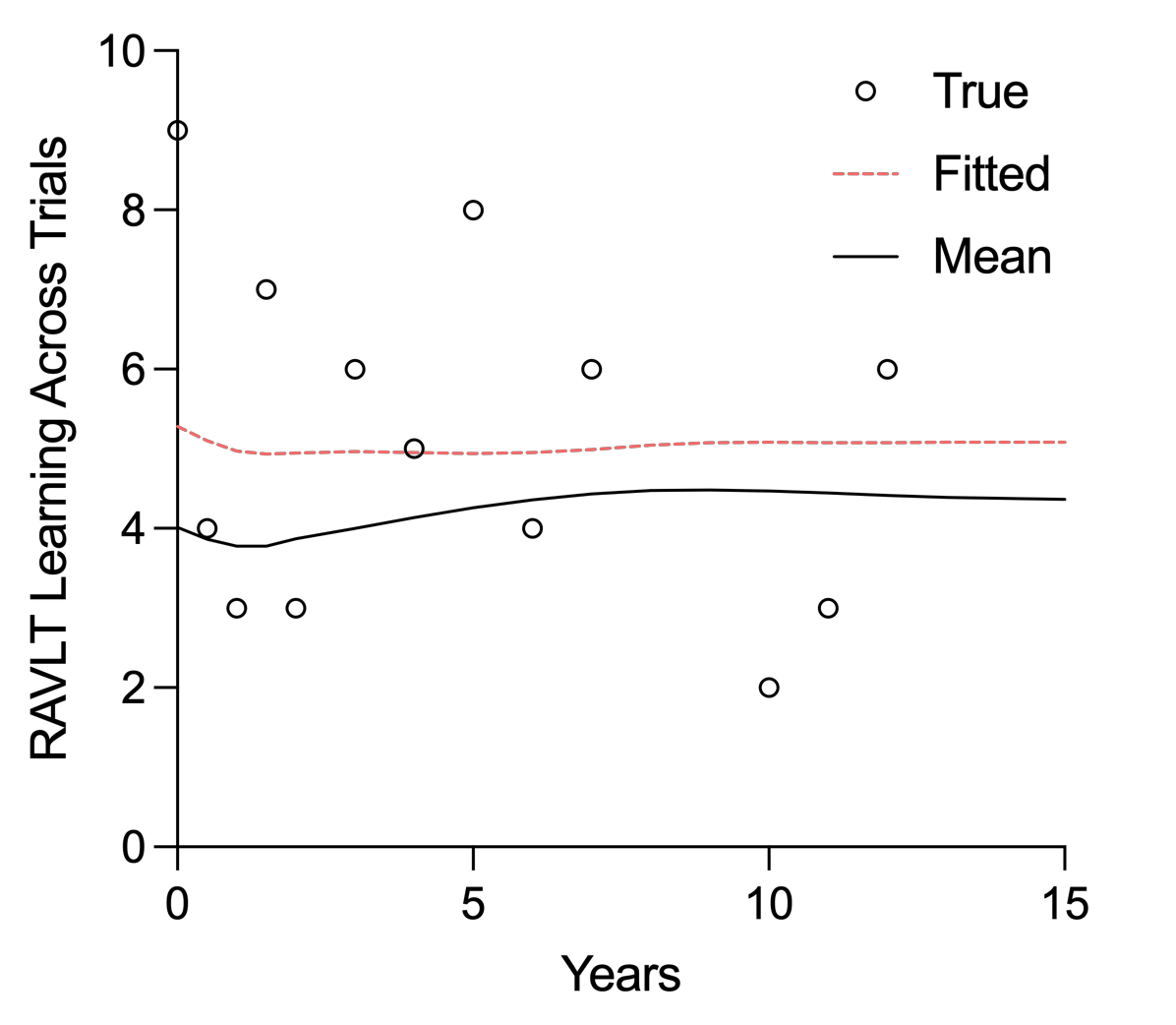

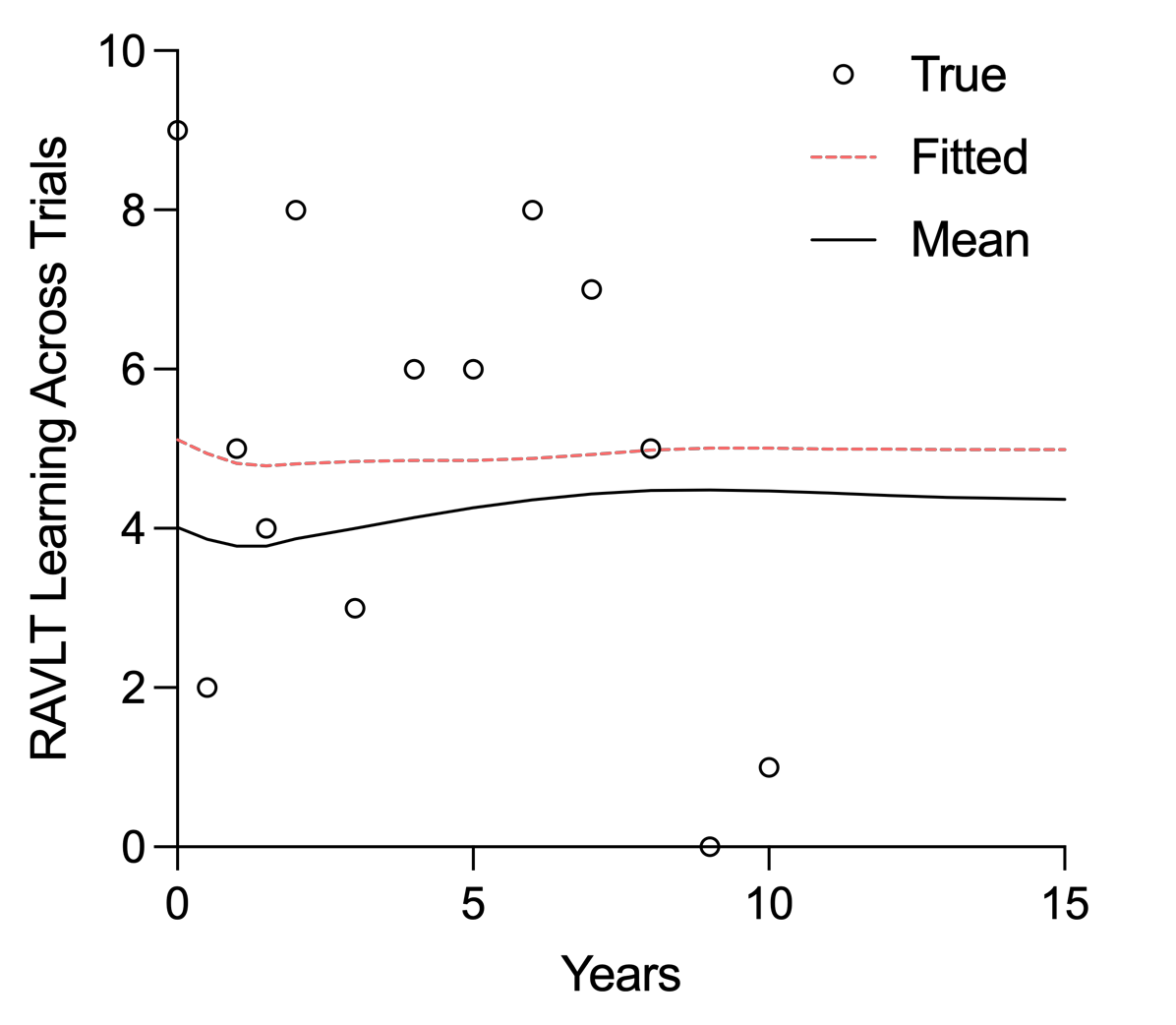


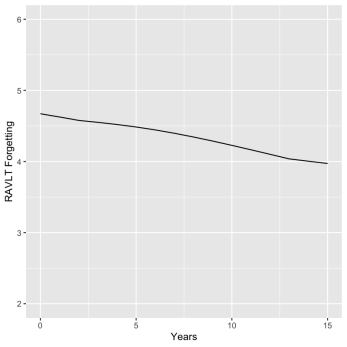

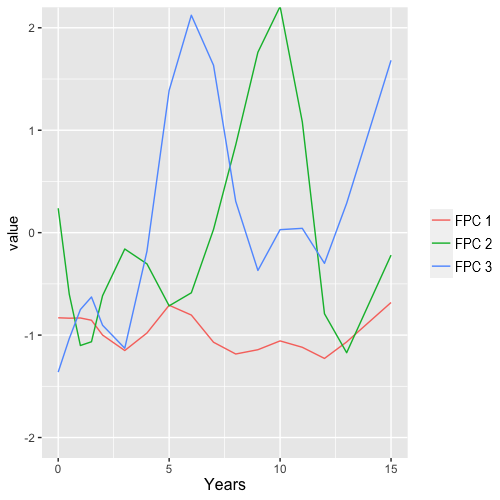

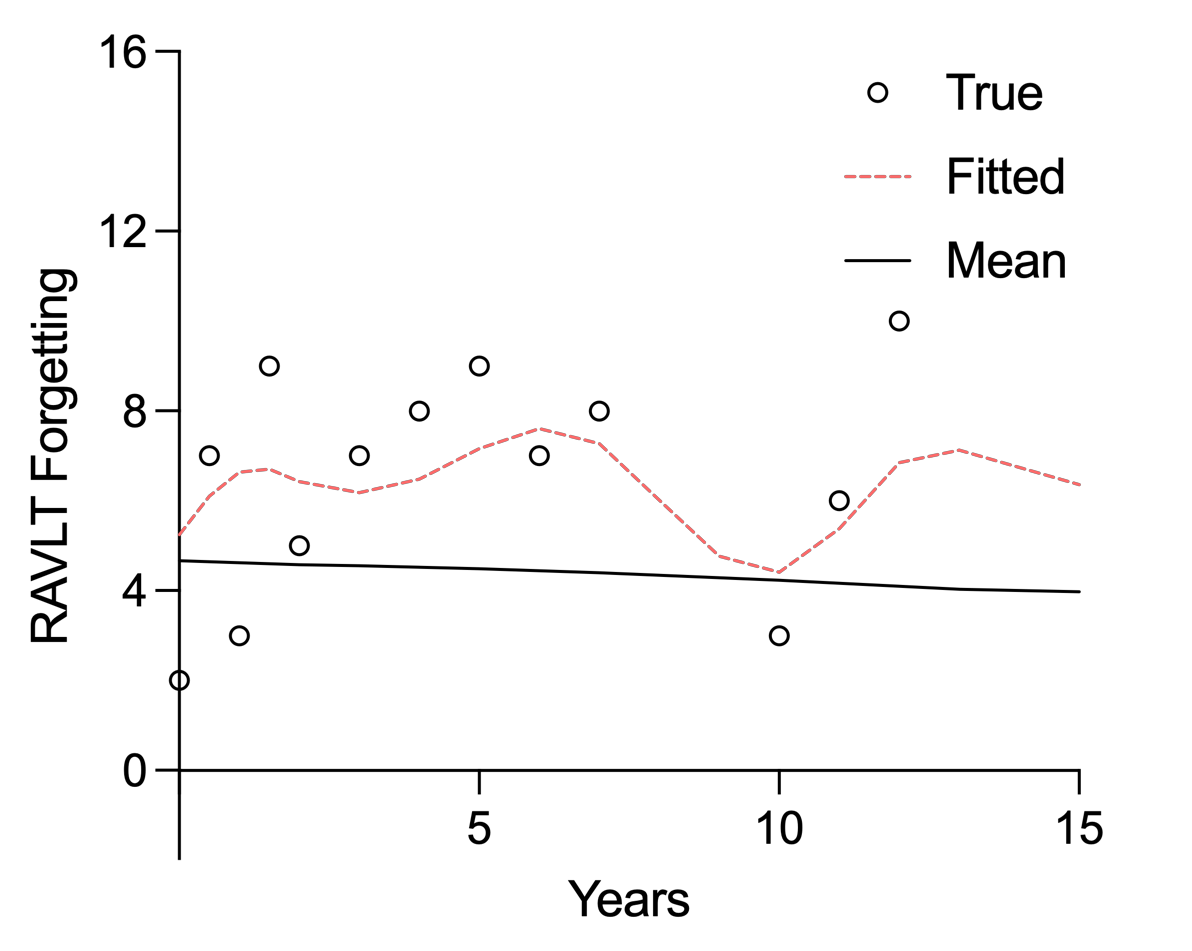

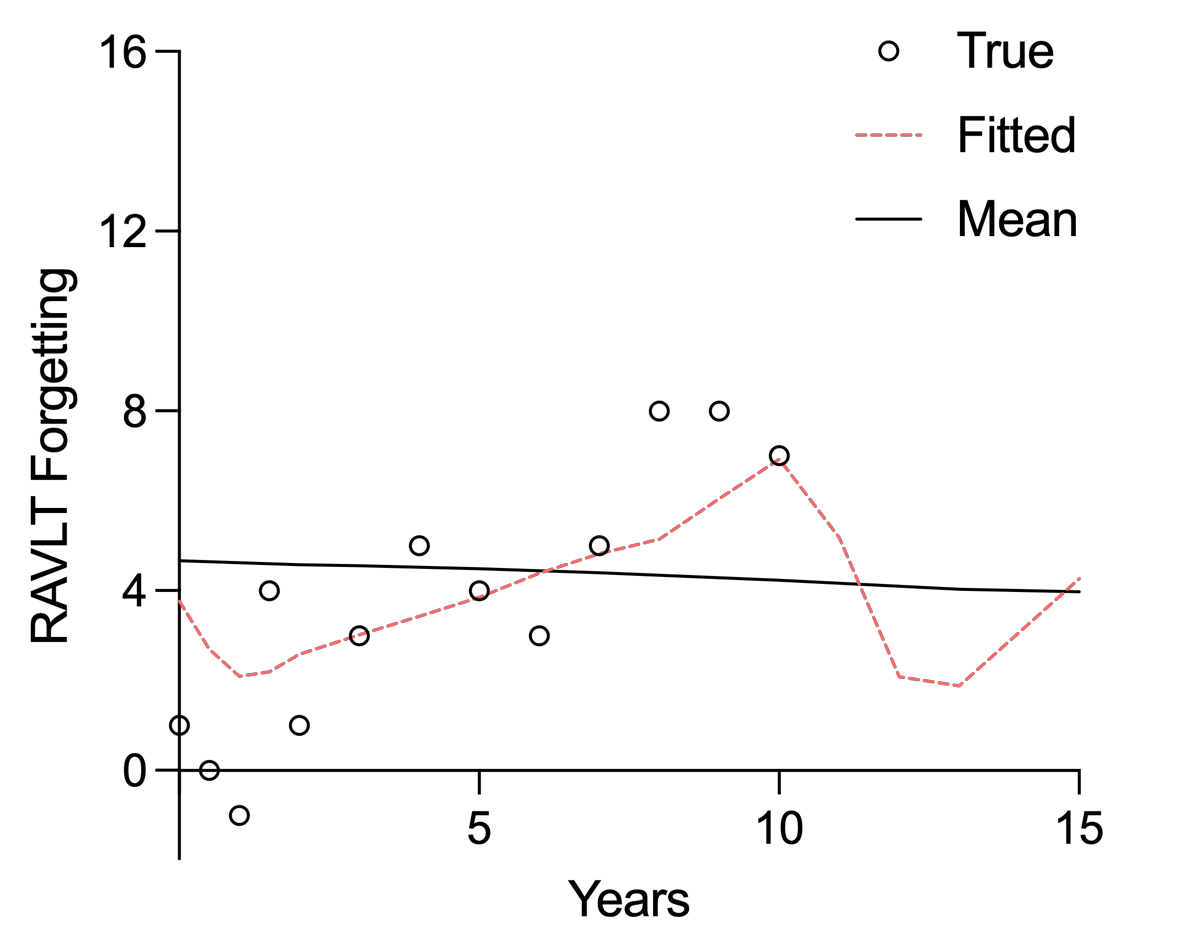


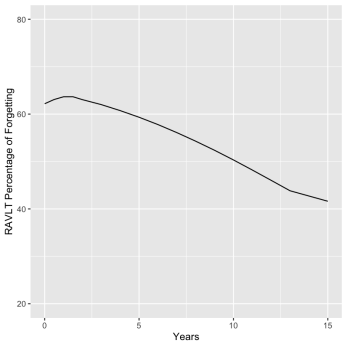

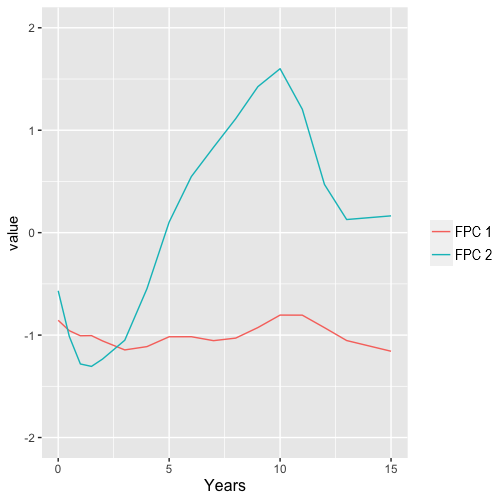

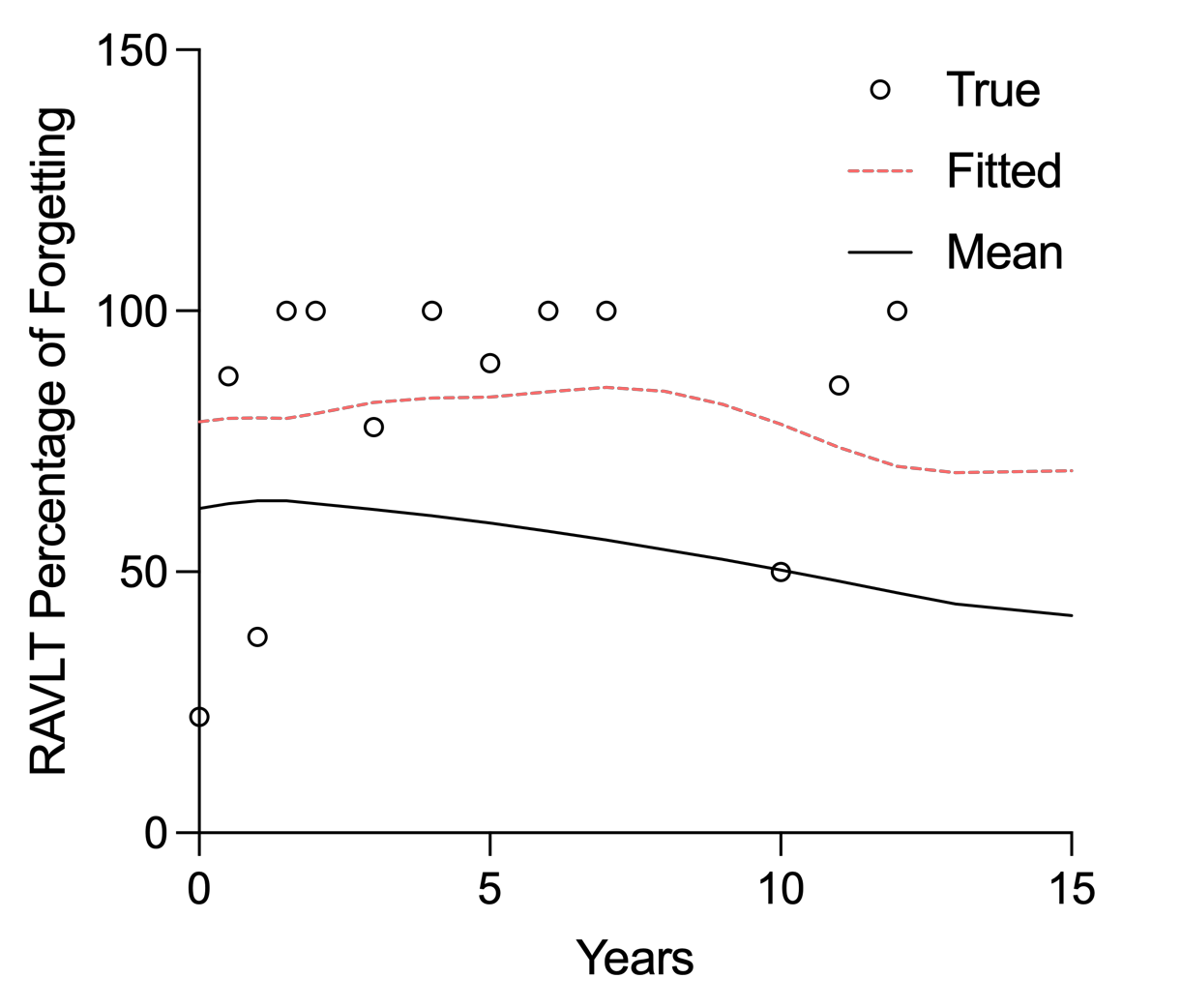

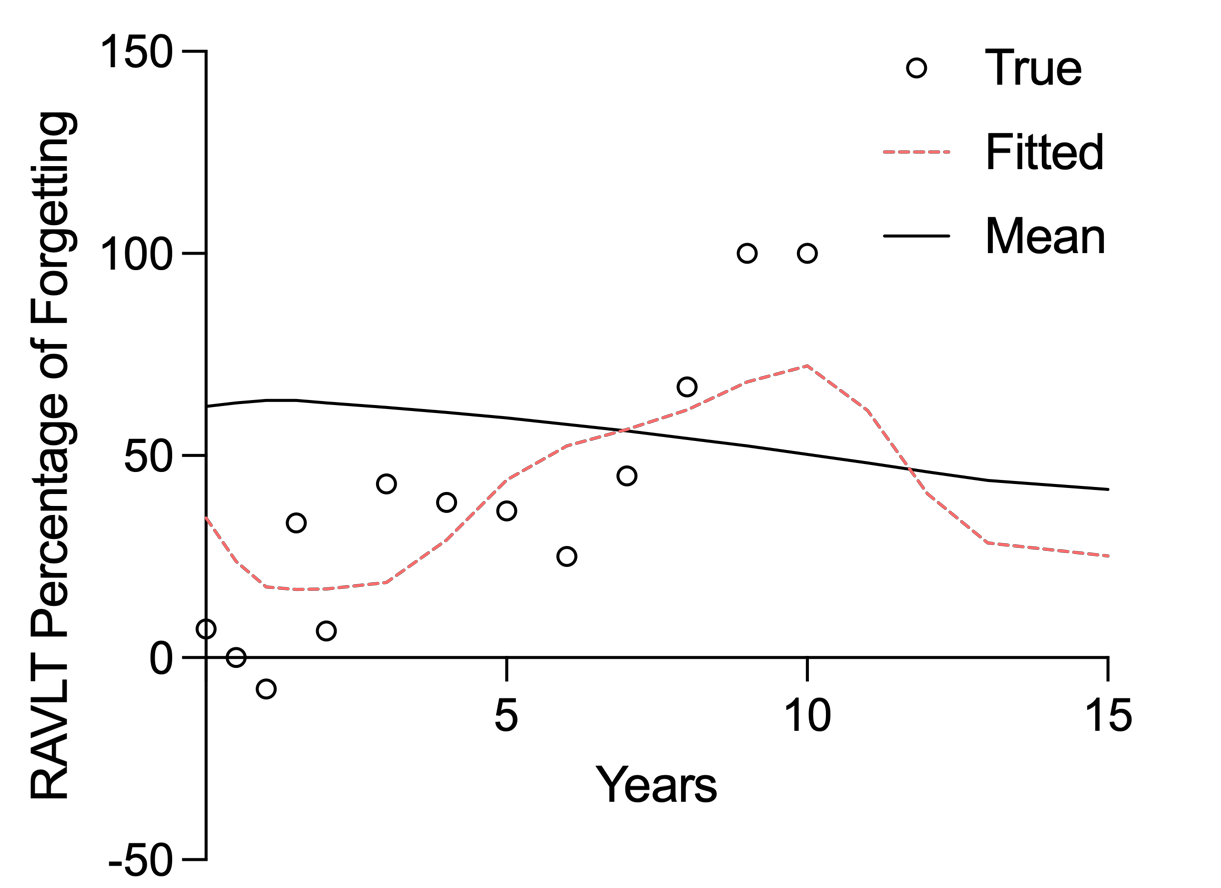


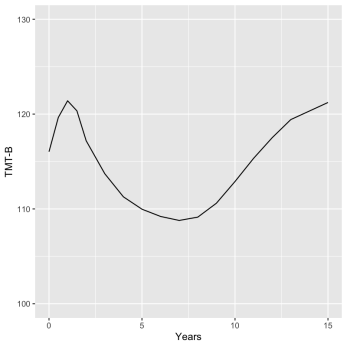

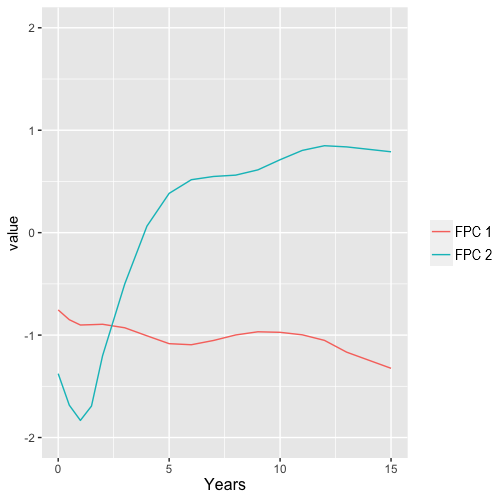

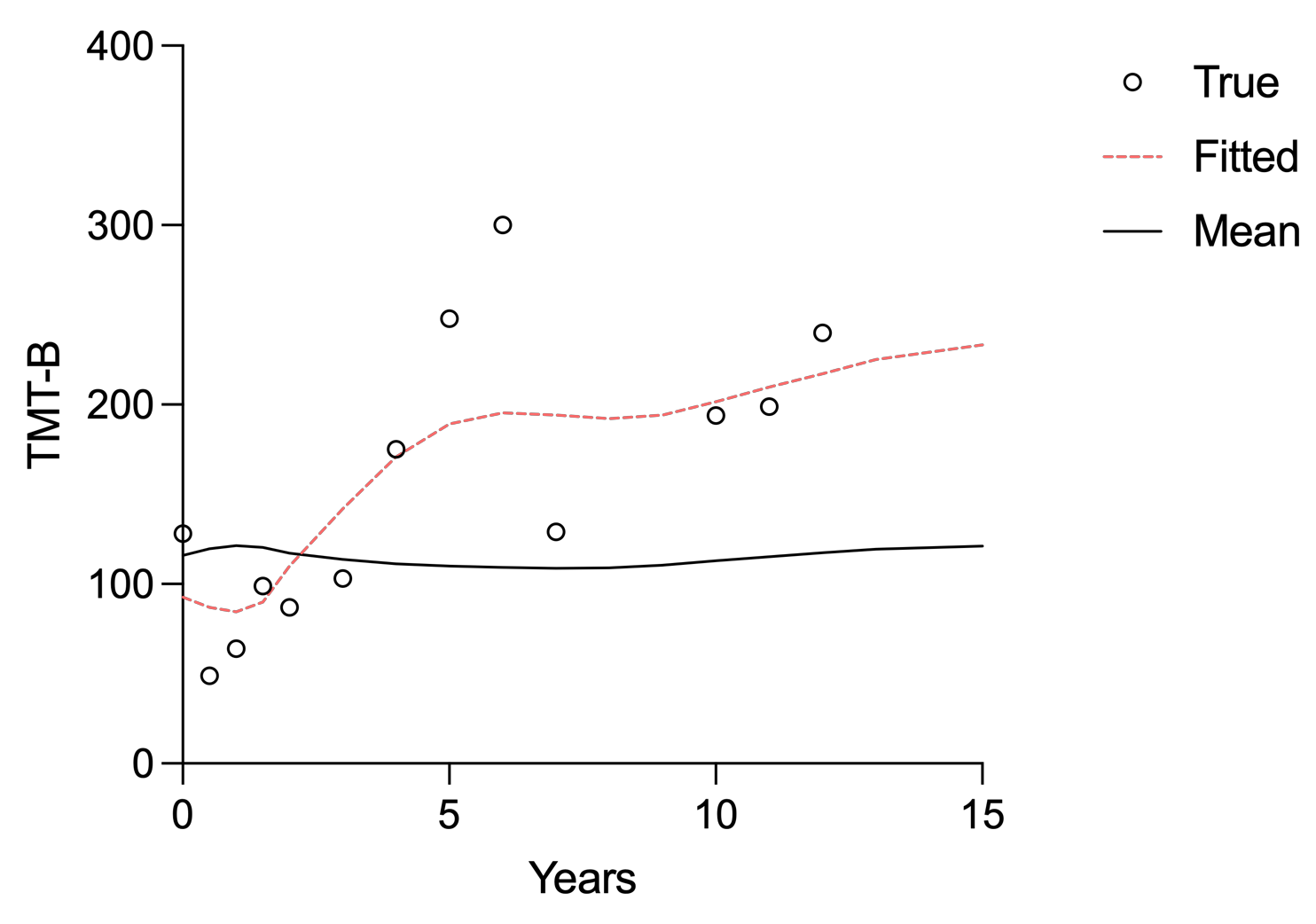

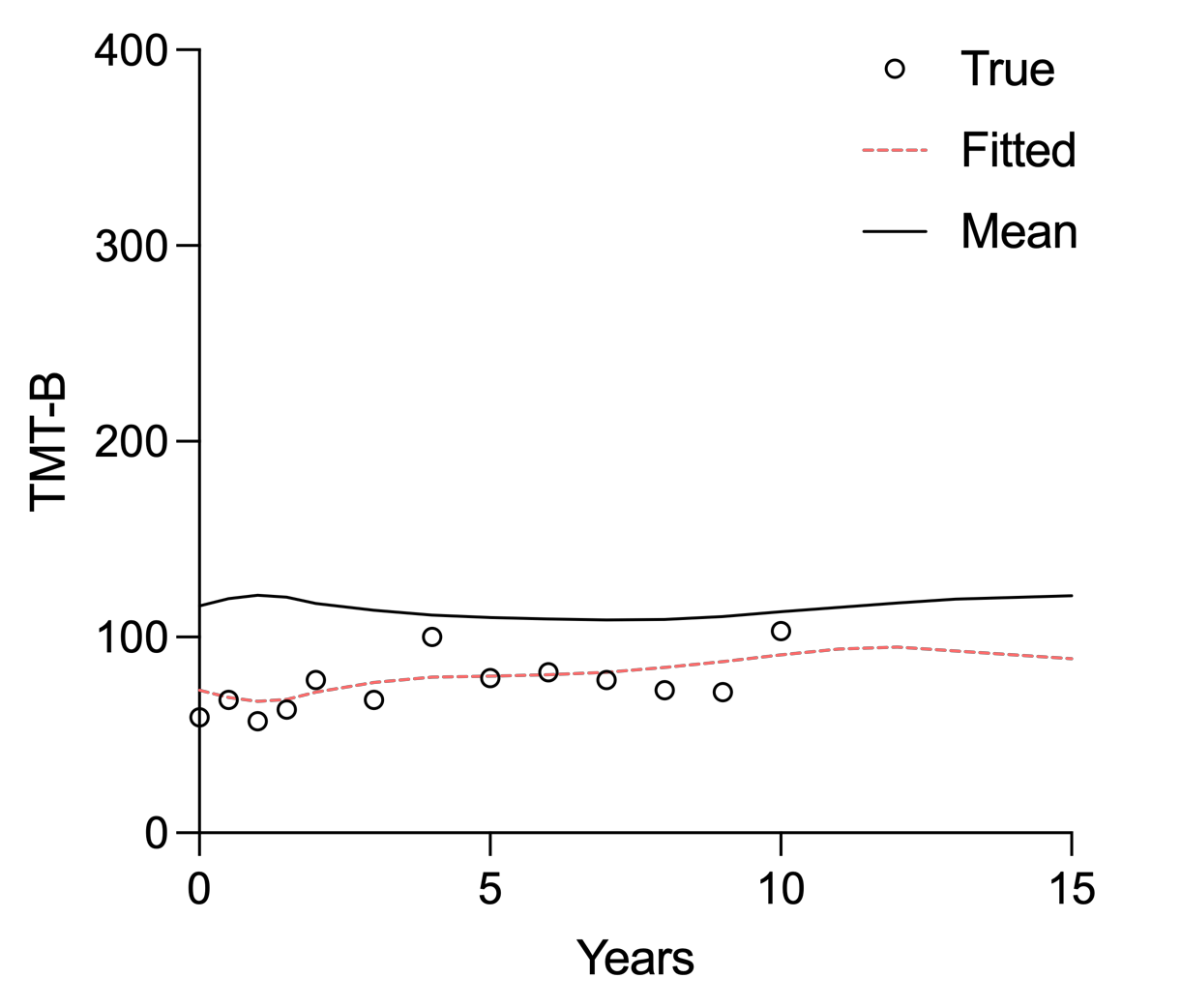


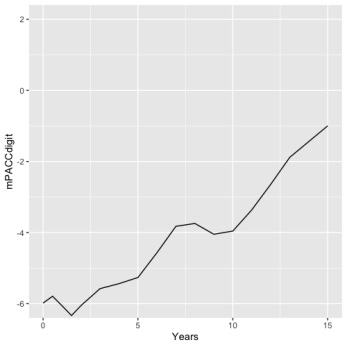

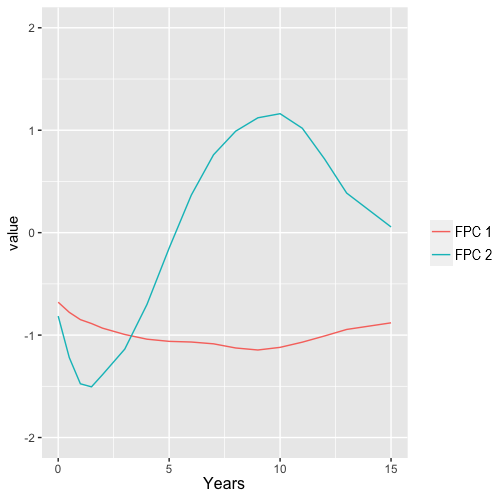

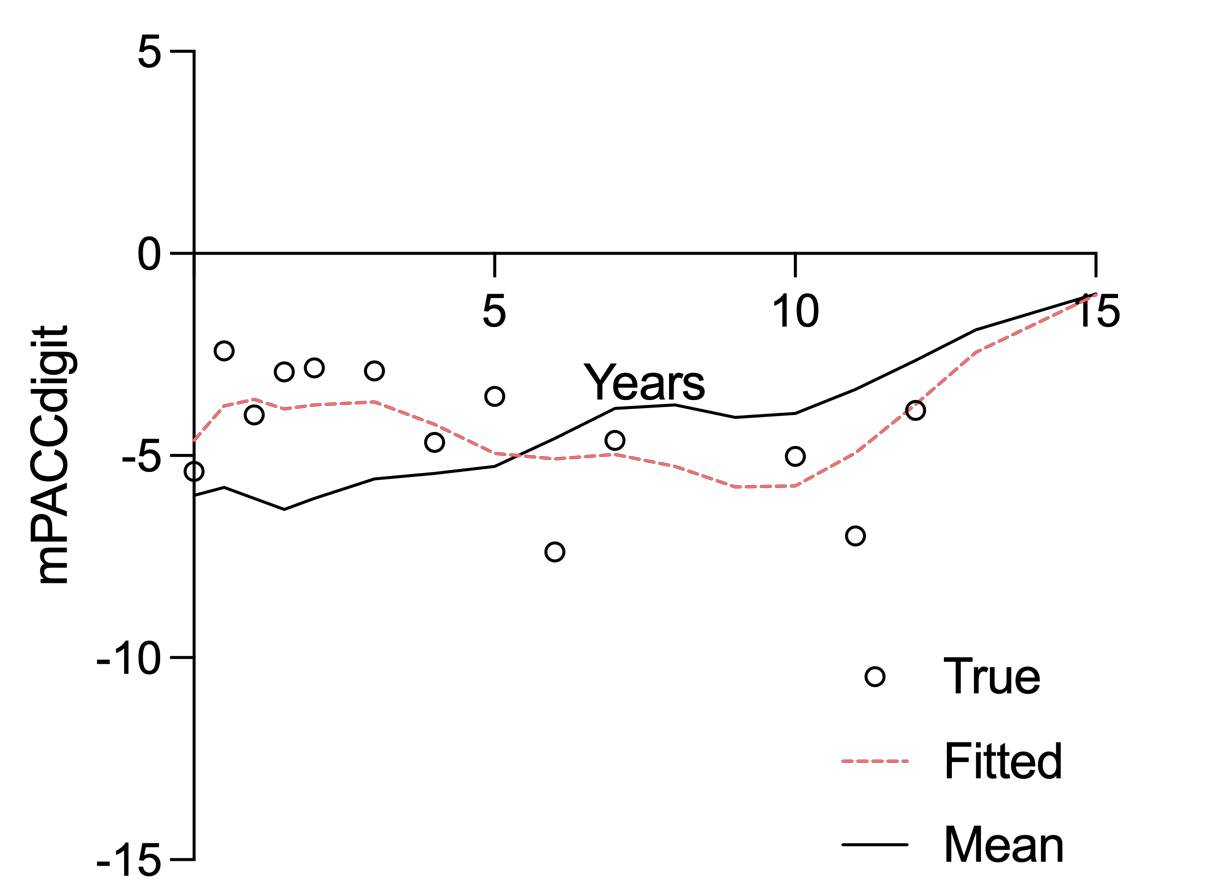

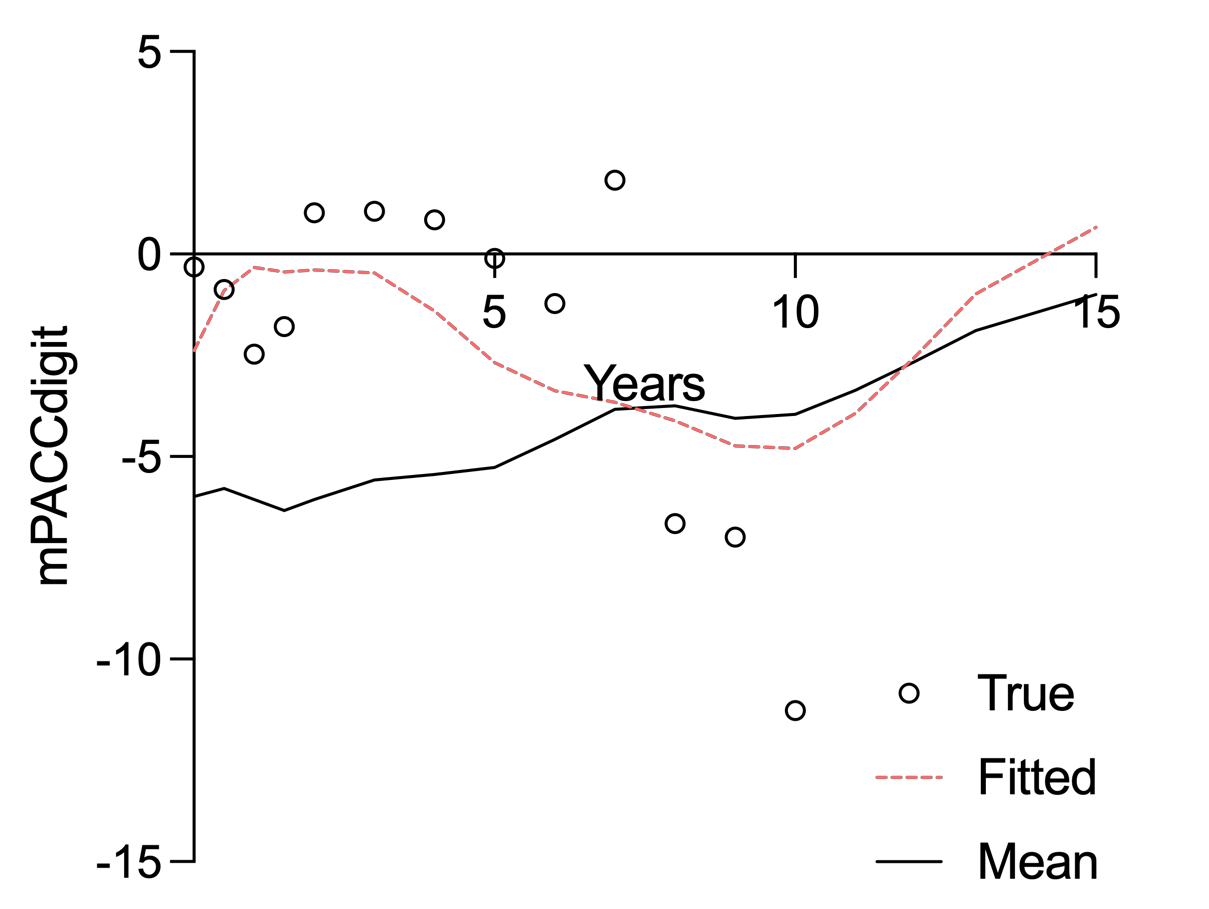


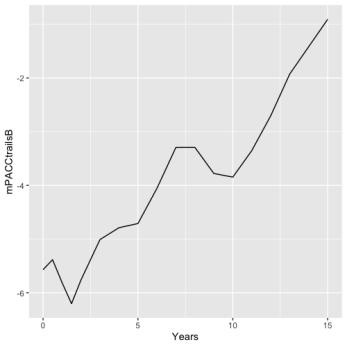

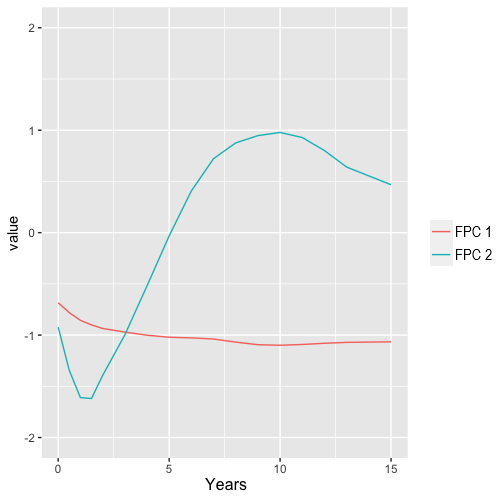

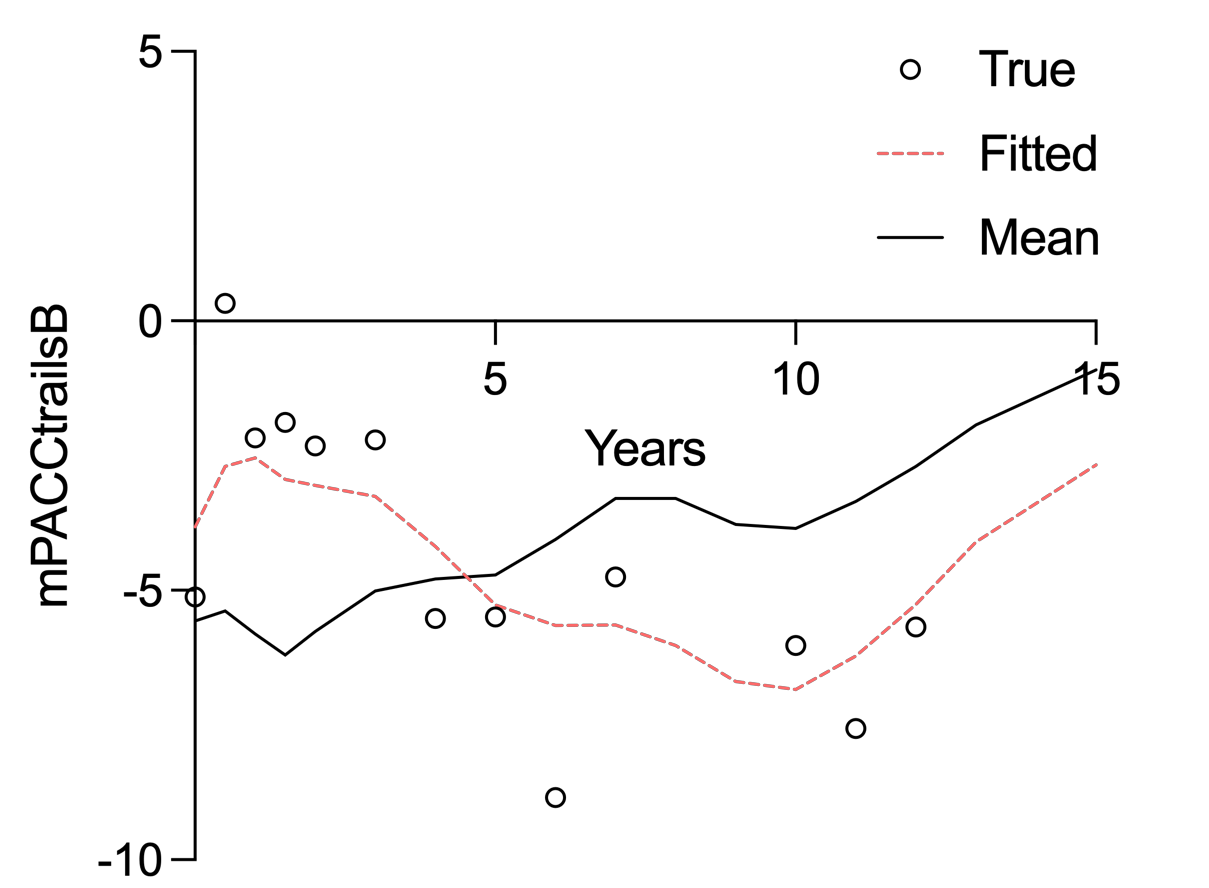

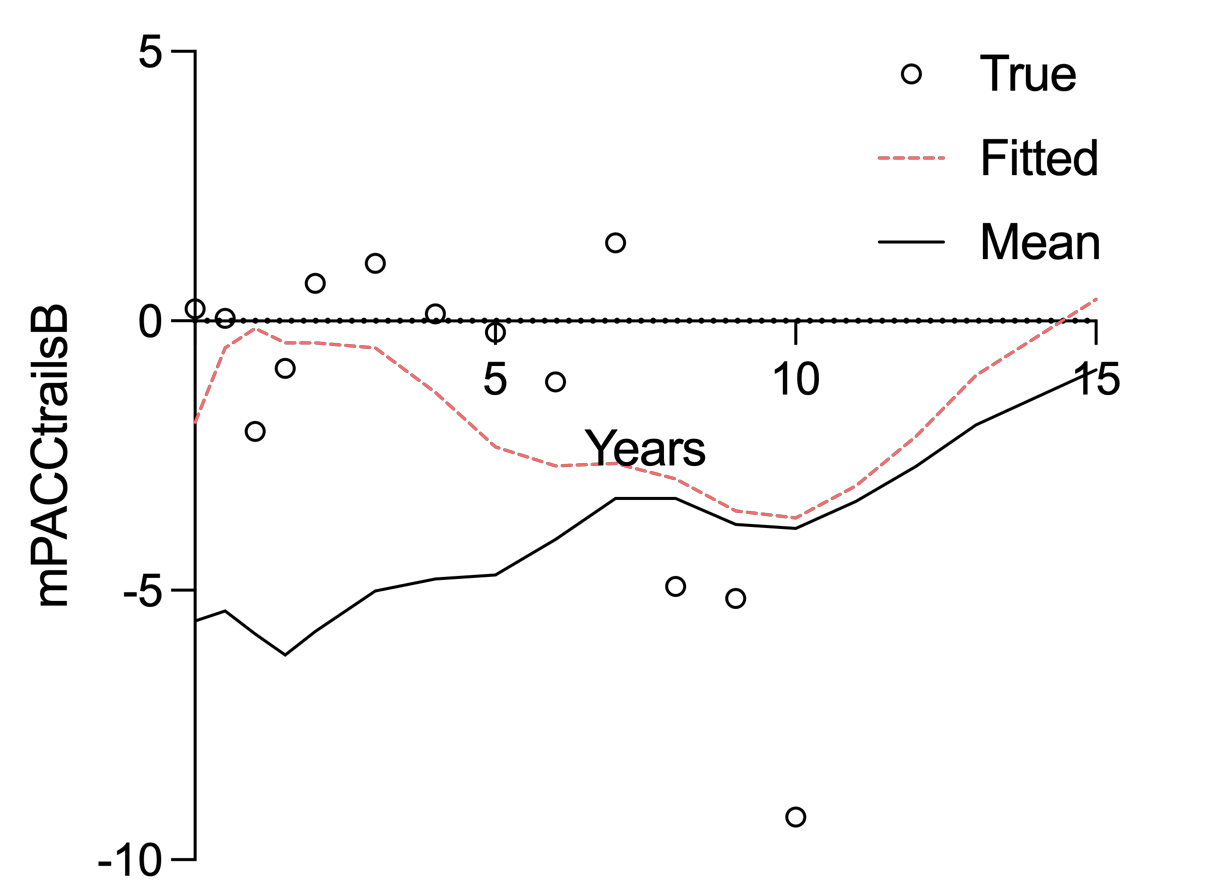


**Figure 4 Distribution of FPCs for each longitudinal cognitive assessment extracted from univariate FPCA across different MCI types.**


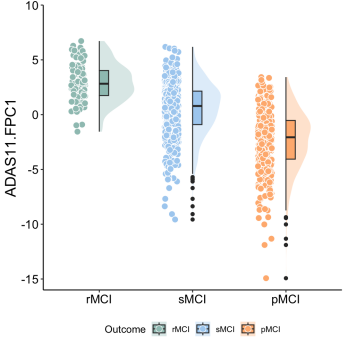

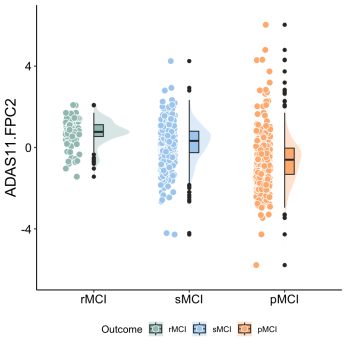

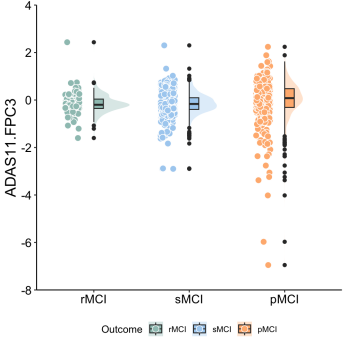

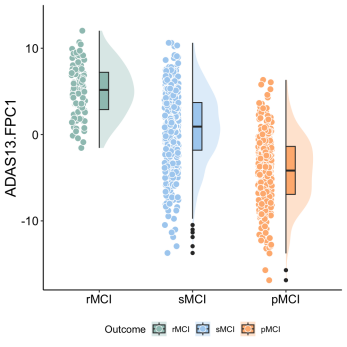

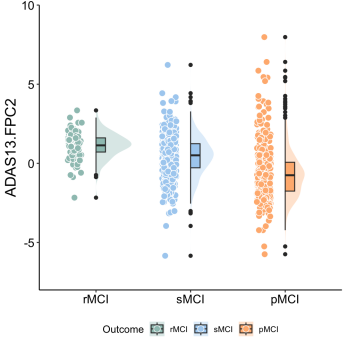


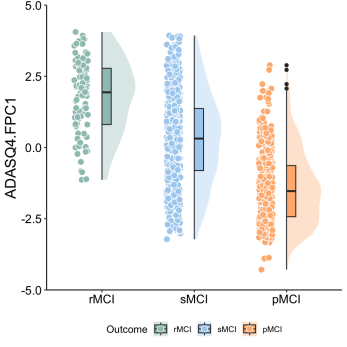

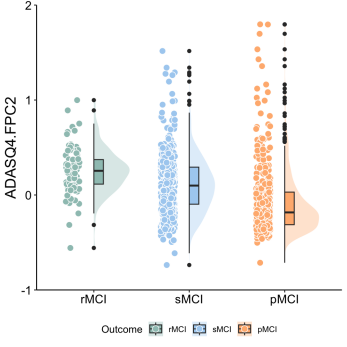

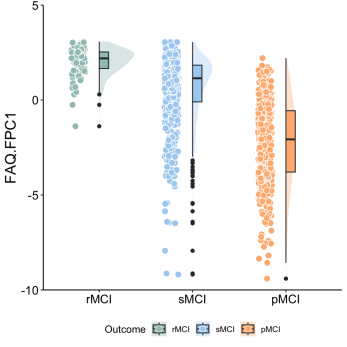

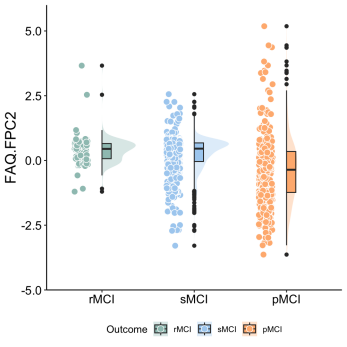

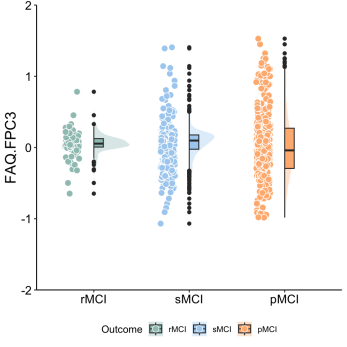


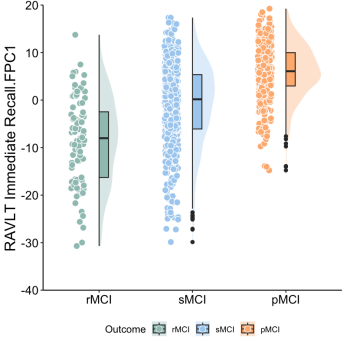

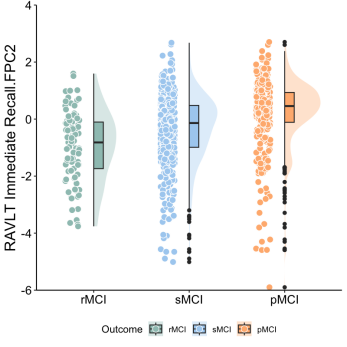


**Figure 5 The mean perturbation curve of MVFPC1, fully showing the changing trend of multiple cognitive assessments.**

Solid black lines were mean function curves, and “+” and “-” were perturbations with appropriate multiples of the weight function of the addition or subtraction MVFPC1. The mean function curves of ADAS11, ADAS13 and ADASQ4 were close to each other, showing a stable trend. FAQ and TMT-B showed a fluctuating trend of increasing or decreasing from time to time. RAVLT Immediate Recall, mPACCdigit and mPACCtrailsB showed a fluctuating trend of increasing year by year. RAVLT Learning Across Trials increased first and then flattened, while RAVLT Forgetting and RAVLT Percentage of Forgetting decreased year by year.

**Figure 6 The trajectories curves of five eigenfunctions obtained by the MVFPCA, representing the five main variation patterns in the random trajectories of the multiple cognitive assessments.**

Taking the eigenfunction curves of MVFPC1 and MVFPC2 as an example, the MVFPC1 of ADAS11, ADAS13, ADASQ4, FAQ, RAVLT Forgetting, RAVLT Percentage of Forgetting and TMT-B were below zero, and the MVFPC1 of RAVLT Immediate Recall, RAVLT Learning Across Trials, mPACCdigit and mPACCtrailsB were above zero. The MVFPC2 of ADAS11, ADAS13, ADASQ4 and FAQ showed a fluctuating upward trend, and the overall value was below zero. The MVFPC2 of RAVLT Immediate Recall and RAVLT Learning Across Trials showed a stable and fluctuating downward trend, respectively, and the overall value was below zero. The MVFPC2 of RAVLT Forgetting and RAVLT Percentage of Forgetting showed a trend of rising and falling from time to time, and the overall value was below zero. MVFPC2 of TMT-B, mPACCdigit, and mPACCtrailsB showed a stable trend, and the overall value was above zero.

**Figure 7 Based on FPCA implementation in the brain space continuous domain, a cubic B-spline function with 116 basis functions and the relationship between smoothing parameters and generalized cross-validation (GCV).**

The optimal smoothing parameter log_10_λ=1.2 was selected to achieve the balance between the smoothness of the function curve and the data fitting, wiht the minimum of generalized cross-validation of 3562.886.

**Figure 8 The eigenfunctions curves of one tMLFPCs from the temporal continuous domain, and two tMLFPCs from the brain spatial continuous domain.**

**Figure 9 Scree plot of PCs extracted from multiple longitudinal cognitive assessments. By setting the cumulative proportion of variance explained (PVE) at a threshold of 90%, we extracted the first five PCs to be included in the subsequent multi-state Markov model.**

**Figure 10 Scree plot of PCs extracted from longitudinal neuroimaging. By setting the cumulative PVE at a threshold of 90%, we extracted the first five PCs to be included in the subsequent multi-state Markov model.**

**Figure 11 The observed (solid blue line) and expected (red dashed line) percentages at one-yearly intervals up to 15 years in the FMSM 1, incorporated longitudinal neuroimaging data through FPC scores in the spatial domain.**

**Figure 12 The Kaplan-Meier estimate of the survival probability and the fitted survival probability from the FMSM 1, incorporated longitudinal neuroimaging data through FPC scores in the spatial domain.**

**Figure 13 The observed (solid blue line) and expected (red dashed line) percentages at one-yearly intervals up to 15 years in the FMSM 2, incorporated longitudinal neuroimaging data through FPC scores in the spatial domain.**

**Figure 14 The Kaplan-Meier estimate of the survival probability and the fitted survival probability from the FMSM 2, incorporated longitudinal neuroimaging data through FPC scores in the spatial domain.**

**Figure 15 The observed (solid blue line) and expected (red dashed line) percentages at one-yearly intervals up to 15 years in the FMSM 3, incorporated longitudinal neuroimaging data through fMLFPC scores in the spatial-temporal two-dimensional domain.**

**Figure 16 The Kaplan-Meier estimate of the survival probability and the fitted survival probability from the FMSM 3, incorporated longitudinal neuroimaging data through fMLFPC scores in the spatial-temporal two-dimensional domain.**

**Figure 17 The observed (solid blue line) and expected (red dashed line) percentages at one-yearly intervals up to 15 years in the FMSM 4, incorporated MVFPC scores derived from eleven longitudinal neuropsychological scales and longitudinal neuroimaging data through FPC scores in the spatial domain.**

**Figure 18 The Kaplan-Meier estimate of the survival probability and the fitted survival probability from the FMSM 4, incorporated MVFPC scores derived from eleven longitudinal neuropsychological scales and longitudinal neuroimaging data through FPC scores in the spatial domain.**

**Figure 19 The observed (solid blue line) and expected (red dashed line) percentages at one-yearly intervals up to 15 years in the FMSM 5, incorporated MVFPC scores derived from eleven longitudinal neuropsychological scales and longitudinal neuroimaging data through fMLFPC scores in the spatial-temporal two-dimensional domain.**

**Figure 20 The Kaplan-Meier estimate of the survival probability and the fitted survival probability from the FMSM 5, incorporated MVFPC scores derived from eleven longitudinal neuropsychological scales and longitudinal neuroimaging data through fMLFPC scores in the spatial-temporal two-dimensional domain.**

**Figure 21 The observed (solid blue line) and expected (red dashed line) percentages at one-yearly intervals up to 15 years in the MSM 1, incorporated longitudinal cognitive information by including the PC scores derived from the eleven cognitive assessments.**

**Figure 22 The Kaplan-Meier estimate of the survival probability and the fitted survival probability from the MSM 1, incorporated longitudinal cognitive information by including the PC scores derived from the eleven cognitive assessments.**

**Figure 23 The observed (solid blue line) and expected (red dashed line) percentages at one-yearly intervals up to 15 years in the MSM 2, incorporated longitudinal neuroimaging information by including the PC scores.**

**Figure 24 The Kaplan-Meier estimate of the survival probability and the fitted survival probability from the MSM 2, incorporated longitudinal neuroimaging information by including the PC scores.**

**Figure 25 The observed (solid blue line) and expected (red dashed line) percentages at one-yearly intervals up to 15 years in the MSM 3, combining longitudinal markers from MSMs 1 and 2.**

**Figure 26 The Kaplan-Meier estimate of the survival probability and the fitted survival probability from the MSM 3, combining longitudinal markers from MSMs 1 and 2.**

**Figure 27 Follow-up duration and total visits for different MCI types.**

The total follow-up duration for rMCI was 6.24±3.36 years, with a median of 6 years (IQR: 3, 9); for sMCI, it was 3.14±2.69 years, with a median of 2 years (IQR: 1, 4); and for pMCI, it was 2.51±2.08 years, with a median of 2 years (IQR: 1, 3). Additionally, we observed that the total duration of follow-up visits for rMCI was 6.03±2.66, with a median of 6 (IQR: 4, 7.5); for sMCI, it was 4.52±2.36, with a median of 3 (IQR: 4, 5); and for pMCI, it was 4.26±2.01, with a median of 3 (IQR: 4, 5). These findings suggest that rMCI individuals had longer follow-up durations and more follow-up visits compared to sMCI and pMCI, potentially due to their better cognitive function and lower likelihood of progressing to AD.

**Figure 28 Lowess curves for 11 cognitive assessments across these six different follow-up duration groups.**

The total follow-up duration was stratified into six groups: 0-2 years (564, 55.35%), 3-4 years (251, 24.63%), 5-6 years (68, 6.67%), 7-8 years (60, 5.89%), 9-10 years (46, 4.51%), and >10 years (30, 2.94%). The results reveal a trend of decreasing cognitive function with shorter total follow-up durations.

**Figure 29 Transition intensity across different cognitive states estimated by Bootstrapping with 1000 resamplings.**

The violin plot displays the distribution of intensity across three cognitive states transitions: MCI→AD, MCI→rNC, and rNC→MCI. The width of each violin reflects the density of data points at different intensity levels. The MCI→AD and MCI→rNC groups show narrow distributions with low variability, while the rNC-MCI group has a much wider distribution, indicating higher variability in intensity. The boxplots within the violins highlight the median and interquartile ranges for each group, providing a visual comparison of central tendency and spread.
